# Design and biological evaluation of 3-substituted quinazoline-2,4(1*H*,3*H*)-dione derivatives as dual c-Met/VEGFR-2-TK inhibitors

**DOI:** 10.1080/14756366.2023.2189578

**Published:** 2023-03-15

**Authors:** Abdelfattah Hassan, Fawzy A. F. Mubarak, Ihsan A. Shehadi, Ahmed M. Mosallam, Hussain Temairk, Mohamed Badr, Aboubakr H. Abdelmonsef

**Affiliations:** a Department of Medicinal Chemistry, Faculty of Pharmacy, South Valley University, Qena, Egypt; b Department of Chemistry, Faculty of Science, South Valley University, Qena, Egypt; c Department of Chemistry, College of Sciences, Pure and Applied Chemistry Research Group, University of Sharjah, Sharjah, United Arab Emirates; d Department of Biochemistry, Faculty of Pharmacy, Menoufia University, Menoufia, Egypt

**Keywords:** c-Met, VEGFR-2, colorectal cancer, quinazoline-24(1*H*3*H*)-dione, *N*-acylhydrazone

## Abstract

The dual c-Met/vascular endothelial growth factor receptor 2 (VEGFR-2) TK inhibition is a good strategy to overcome therapeutic resistance to small molecules VEGFR-2 inhibitors. In this study, we designed 3-substituted quinazoline-2,4(1*H*,3*H*)-dione derivatives as dual c-Met/VEGFR-2 TK inhibitors. We introduced new synthetic methods for reported derivatives of 3-substituted quinazoline-2,4(1*H*,3*H*)-dione **2a**–**g**, in addition to the preparation of some new derivatives namely, **3** and **4a**–**j**. Three compounds namely, **2c**, **4b**, and **4e** showed substantial amount of inhibition for both c-Met and VEGFR-2 TK (IC_50_ range 0.052–0.084 µM). Both compounds **4b**, **4e** showed HB with highly conserved residue Asp1222 in the HB region of c-Met TK. For VEGFR-2 TK, compound **4b** showed HB with a highly conserved residue Asp1046 in the HB region. Compound **4e** showed HB with Glu885 and Asp1046. Moreover, *in silico* prediction of pharmacokinetic and physicochemical parameters of target compounds was carried out using SwissADME website. The quinazoline-2,4(1*H*,3*H*)-dione derivatives are promising antiproliferative candidates that require further optimisation.

**Highlights**
- New 3-substituted quinazoline-2,4(1*H*,3*H*)-dione derivatives were synthesised and characterised.
- Compounds **4b** and **4e** showed higher cytotoxic activity than cabozantinib against HCT-116 colorectal cell lines.
- Both compounds **4b** and **4e** showed less toxicity to WI38 normal cell line compared to HCT 116 colon cancer cell line.
- Compound **4b** was superior to cabozantinib in VEGFR-2 inhibition while compound **2c** was equipotent to cabozantinib.
- Compounds **4b** and **4e** showed remarkable c-Met inhibitory activity.
- Compounds **4b** and **4e** arrested cell cycle and induced significant levels of apoptosis.
- *In silico* ADME prediction revealed high oral bioavailability and enhanced water solubility of target compounds as compared to cabozantinib.
- Target compounds interacted with both c-Met and VEGFR-2 active site in similar way to cabozantinib.

## Introduction

Globally, cancer kills about 10 million people every year and its mortality rate will reach 16.3 million deaths in 2040^1^. Conventional chemotherapeutic drugs can raise the survival rate of cancer patients but they have negative impact on patients’ quality of life because of their severe adverse effects^2^. With growing knowledge of chemistry and function of cellular molecular targets, tyrosine kinase (TK) inhibitors become the main arm of chemotherapeutic arsenal^3^. Compared to conventional chemotherapy, TK inhibitors afford good anticancer activity with few and tolerable adverse effects^2^.

The activation of c-Met TK receptor is achieved by binding of HGF/SF, that binding promotes different cellular signalling pathways and results finally in cell proliferation, motility, migration, and survival^4^. These cellular activities are fundamental in normal physiological processes like wound healing but can lead to tumorigenesis when these pathways are deregulated^5^. c-Met TK was found to be overexpressed or mutated in human tumours and it was related to poor prognosis^4^. Consequently, c-Met was considered as a potential molecular target for cancer therapy^6^.

Another TK is a vascular endothelial growth factor receptor 2 (VEGFR-2), which is expressed in endothelial cells, mediated by VEGF, and fundamentally regulates the angiogenesis of tumours^7^^,^^8^. Albeit VEGF/VEGFR pathway is overexpressed in cancer cells and it is considered as important drug target, many antiangiogenic drugs suffering from humble efficacy^9^. Many neoplasms acquired therapeutic resistance against bevacizumab as well as small molecules inhibitors that target catalytic domain of VEGFR-2^10–13^. There are several mechanisms beyond this resistance like expression of Tie2 by macrophage^9^, tumour hypoxia^14^, upregulation of SDF,1^15^ and overexpression of pERK^16^. It is found that HGF/c-Met pathway drives this resistance and dual inhibition of c-Met/VEGFR-2 was found to be effective against tumours that are resistant to pan-VEGFR inhibition^14^^,^^17^. Single-target VEGFR-2 inhibitors does increase hypoxia which leads to c-Met mediated invasion and metastasis of cancer cells. On the contrary, the combination of VEGFR-2 and c-Met inhibitors, like sunitinib and PF-04217903 remarkably lowers tumour invasion and metastasis.^18^ Hence regarding drug resistance, multitarget TK inhibitors are more superior than single target inhibitors^19^.

**Figure 1.**
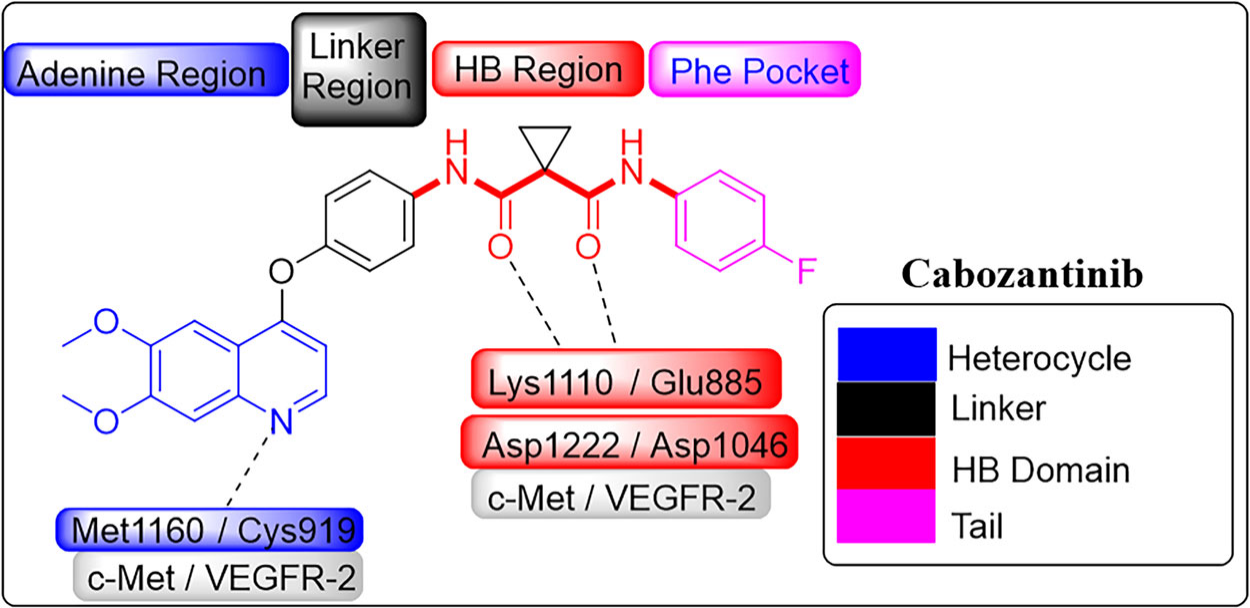
Pharmacophoric elements for dual inhibition of c-Met/VEGFR-2 TKs.

**Figure 2.**
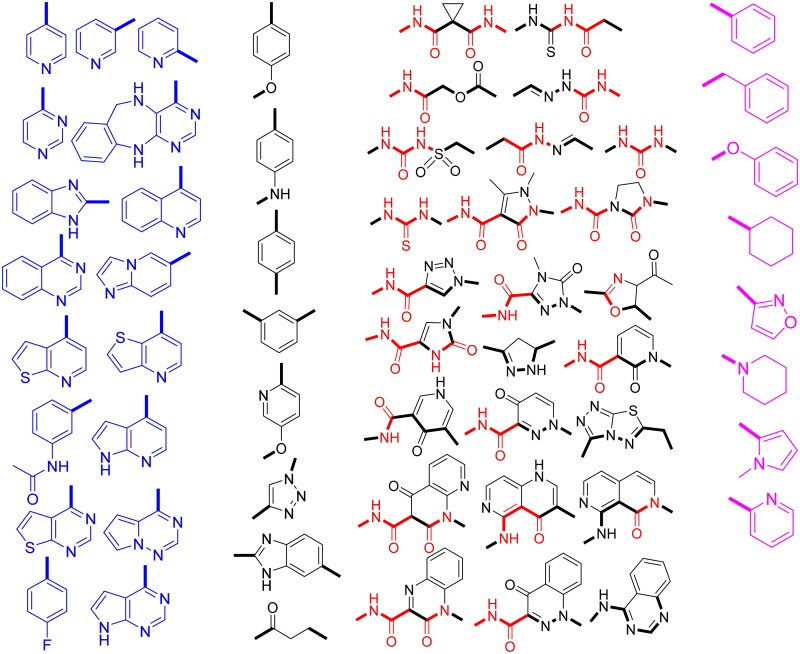
Four structural elements of reported dual c-Met/VEGFR-2 inhibitors.

Four structural requirements for dual c-Met/VEGFR-2 inhibition are heterocycle, linker, HB domain, and hydrophobic tail (Figure 1)^19^. Aza-heterocycle occupies adenine site in hinge region and forms from 0 to 3 HBs with highly conserved residues Met1160 and Cys919 in c-Met and VEGFR-2, respectively. Heterocycle ring can be surrogated by phenyl ring with HB groups like halogens and carboxamide (Figure 2)^18^^,^^20^. Linker occupied the region adjacent to gatekeeper. It has a little role in protein binding, but it is important to fix other pharmacophoric elements in the corresponding binding sites^8^. It may be (hetero)aromatic or aliphatic chain (Figure 2)^6^^,^^7^^,^^18^^,^^20–22^.

HB domain can form HB with Asp1046 and Asp1222 of the DFG motif in c-Met and VEGFR-2, respectively. For c-Met inhibition, HB domain acts as HBA by forming two HB by two carbonyl oxygen with Asp1222 and Lys1110 while for VEGFR-2 inhibition, HB domain acts as dual HBA/HBD by forming two HB by carbonyl oxygen and amidic NH with Asp1046 and Glu885^8^^,^^23^. HB domain can be dicarboxamide, 3-sulfonylacrylamide, (thio)urea, *N*-acyl(thio)urea, carbamoylmethyl carboxylate, *N*-acylhydrazone, and semicarbazone^3^^,^^18^^,^^22^^,^^24–27^. It also can be incorporated in nitrogenous five or six membered ring system with carboxamide substituent like pyrazole, imidazole, 1,2,3-triazole, pyridine, pyrimidine, pyridazine, quinoline, cinnoline, and naphthyridine^3^^,^^4^^,^^6^^,^^7^^,^^20^^,^^21^^,^^24^^,^^25^^,^^28–33^. Hydrophobic tail occupies the phenyl pocket in the allosteric site of TK and it may be phenyl, (non)aromatic heterocyclic, or alicyclic ring (Figure 2)^3^^,^^19^^,^^34^.

**Figure 3.**
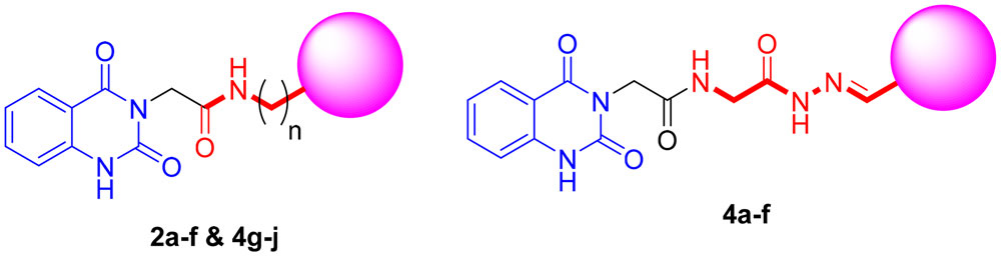
General structures of target compounds.

Therefore, encouraged by the abovementioned rationale, we designed and synthesised a series of 3-substituted quinazoline-2,4(1*H*,3*H*)-dione analogues via a multi component reaction (Figure 3). The antiproliferative activity of target compounds was performed against c-Met/VEGFR-2 overexpressing HCT-116 colon cancer cell line. In addition, cytotoxicity of selected compounds against WI38 normal cells was carried out to assess safety profile of new series of compounds. *In vitro* screening of inhibitory activity against both c-Met and VEGFR-2 was carried out using cabozantinib as a positive control. Cell cycle analysis and apoptosis assay were carried out. Furthermore, the molecular docking studies were performed to investigate the interactions of the target molecules with c-Met and VEGFR-2 TKs. Finally, *in silico* pharmacokinetic profile and druglikeness of the synthesised molecules were investigated by SwissADME.

## Results and discussion

### Chemistry

**Scheme 1.**
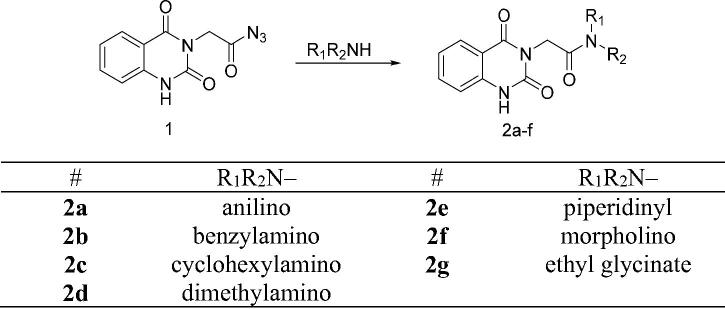
Synthesis of target compounds **2a**–**g**.

**Scheme 2.**
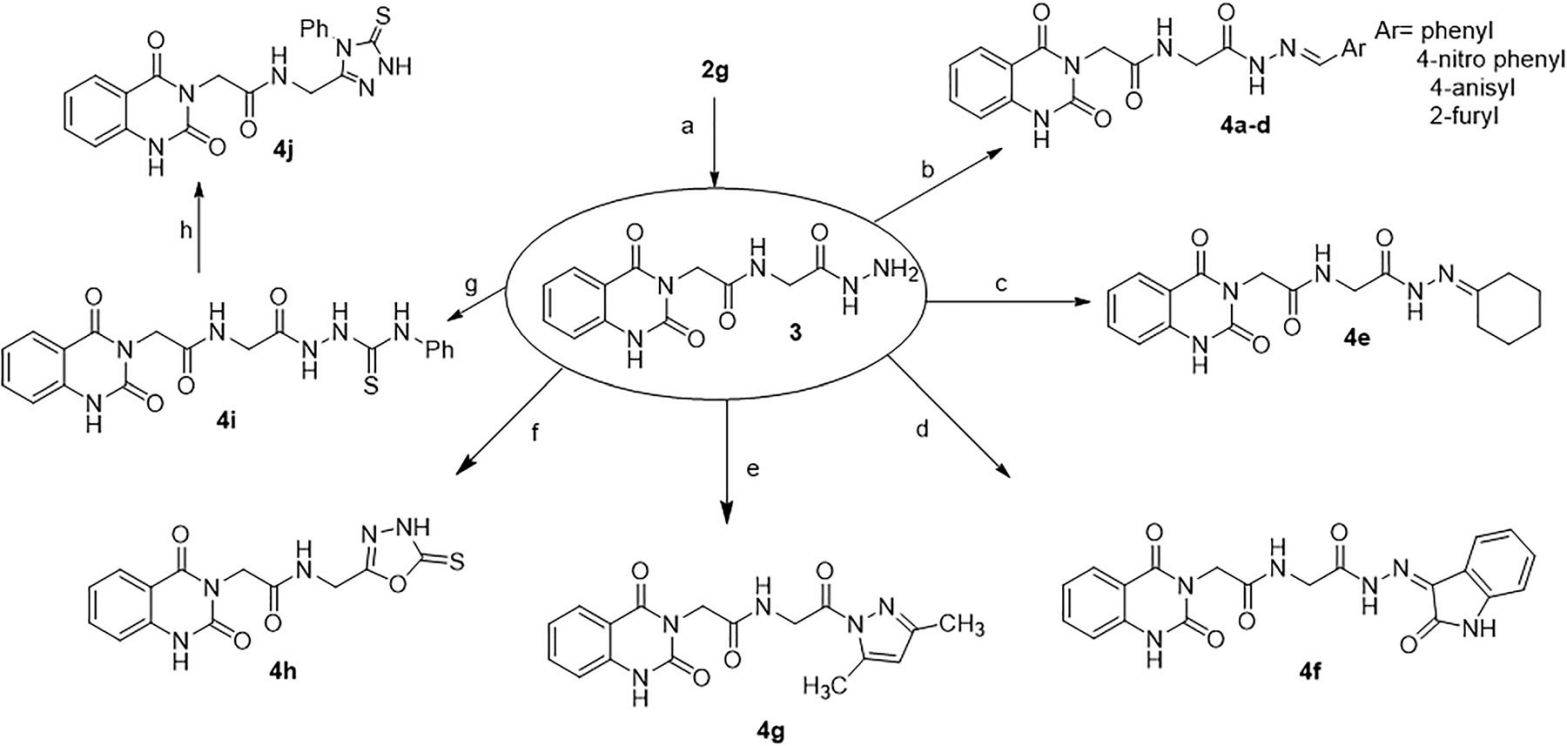
Synthesis of target compounds **3** and **4a**–**j**; reagents and conditions: (a) N_2_H_4_·H_2_O, EtOH, reflux. (b) Ar-CHO, piperidine, EtOH, reflux. (c) Cyclohexanone, EtOH, reflux. (d) Isatin, AcOH, reflux. (e) Acetylacetone, EtOH, reflux. (f) CS_2_, pyridine, reflux. (g) PhNCS, EtOH, reflux. (h) NaOH.

**Figure 4.**
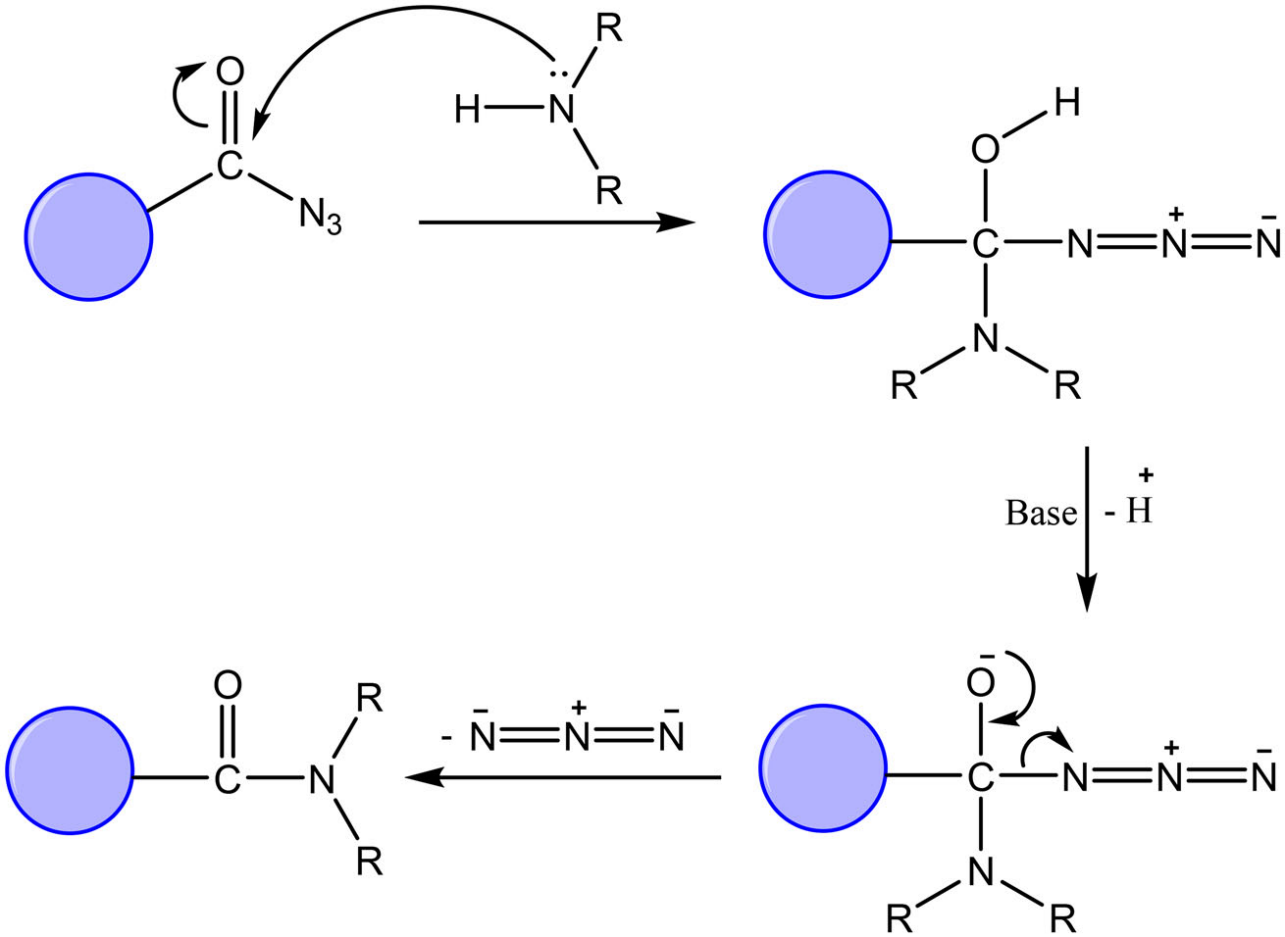
Suggested mechanism of reaction of acyl azide with primary and secondary amines.

The synthetic protocol adopted in this study is illustrated in Schemes 1 and 2. The azide **1** was first prepared by Younes et al. by treatment of a suspension of 2-(2,4-dioxo-1,4-dihydroquinazolin-3(2*H*)-yl)acetohydrazide in hydrochloric acid with a solution of sodium nitrite in water at 0–5 °C^35^. Treatment of a cold solution of azide **1** with different primary and secondary amines afforded a new method to synthesise reported amides **2a**–**g**^36–40^. In these reactions, a low temperature (around 0 °C) is required to prevent decomposition of the acyl azide which releases nitrogen gas. The coupling step was performed under basic conditions. In our study, the amines were used as reactants as well as basic catalysts. For the synthesis of compound **2g**, we used triethylamine as a catalyst. Another method was also used for the synthesis of compounds **2b**, **2c**, **2g** in which it was observed that the coupling reaction can be performed under reflux as it is finished in a time less than that needed for decomposition of the azide. The reflux was carried out in dry benzene for compounds **2b** and **2c**, while dry pyridine was utilised as a solvent and basic catalyst for the synthesis of compound **2g** (Figure 4)^41^.

The reaction of azide **1** with glycine ethyl ester hydrochloride gave **2g**. Then, treatment of compound **2g** with hydrazine hydrate yielded hydrazide **3**. IR spectrum of **3** showed characteristic absorption bands for NH_2_ and NH’s at 3291 and 3175, and for C═O’s at 1704 and 1644. Besides, the ^1^H NMR spectrum revealed the disappearance of the triplet and quartet signals related to the ethyl group of the starting ester **2g**, and the appearance of new characteristic singlet signals for NH and NH_2_ groups of the resulting hydrazide at *δ* 9.02 and 4.26 ppm, respectively. Compound **3** is a useful precursor in the synthesis of target compounds **4a**–**j**.

**Figure 5.**
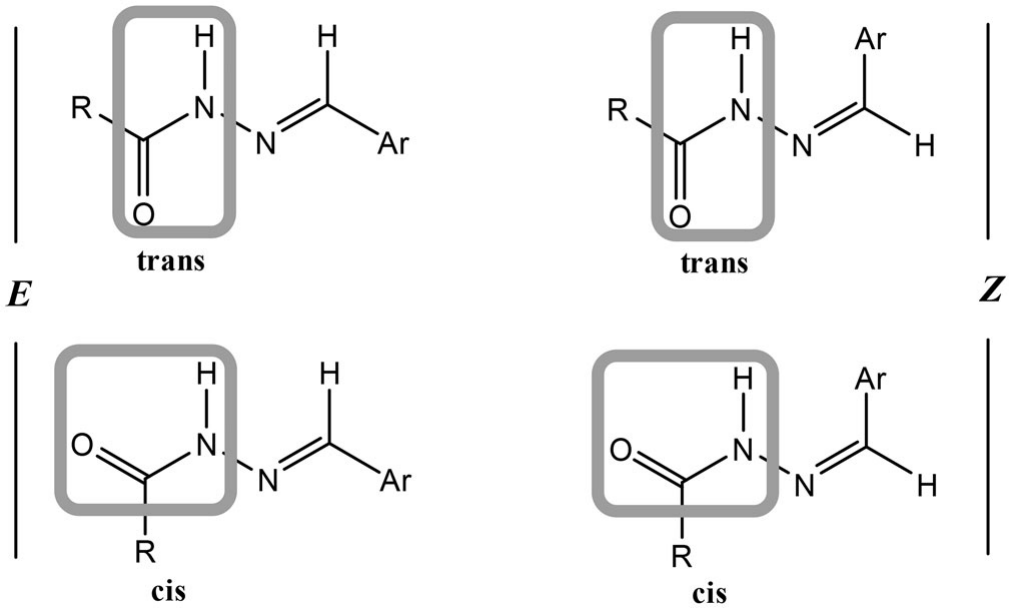
Isomers of *N*-acylhydrazones.

Condensation of hydrazide **3** with various aromatic aldehydes namely, benzaldehyde, 4-nitrobenzaldehyde, anisaldehyde, and furfural afforded a series of hydrazone derivatives **4a**–**d**, respectively in good yields. It is known that *N*-acylhydrazones may exist as *E*/*Z* geometrical isomers with respect to the imine C═N double bond, and *cis*/*trans* amide conformers due to rotation across the amide C(O)–NH single bond (Figure 5)^42^^,^^43^. According to the literature, *N*-acylhydrazones derived from aryl and heteroaryl aldehydes tend to exist in the form of the less hindered *E* geometrical isomer both in the solid state and in DMSO solution; however, in less polar solvents, the *Z* isomer can be detected due to its stabilisation with intramolecular hydrogen bonds.^42–48^

From the spectroscopic studies of the obtained hydrazones **4a**–**d**, IR spectra showed characteristic stretching bands for NH’s in the range of 3249–3127 cm^−1^, for C═O’s in the range of 1736–1644 cm^−1^ and for C═N in the range of 1570–1518 cm^−1^. The ^1^H NMR spectra of these hydrazones exhibited two sets of signals for certain protons (NCH_2_C(O)NH, α-CH_2_, C(O)–NH–N, N═CH), which revealed their existence as an equilibrium mixture from *cis*/*trans* conformers of the *E* geometrical isomer. In addition to the abovementioned studies, this conclusion is supported by previous studies on similar hydrazones.^49^^,^^50^ It was found that the *cis*-conformer predominates in DMSO solution.^42^ For α-CH_2_ protons, the smaller upfield signal is assigned to the *trans*-conformer while the larger downfield signal to the *cis*-conformer. On the contrary, for N═CH proton the larger upfield signal is attributed to the *cis*-conformer while the smaller downfield signal to the *trans*-conformer. These data are in accordance with literature results.^51^ The signals of NCH_2_C(O)NH and C(O)–NH–N protons of *cis*- and *trans*-conformers were recognised from the integration of their signals relative to those of α-CH_2_ and N═CH protons (Table 1). The ratio of *cis*- to *trans*-conformers for every compound was calculated from the ratio of integrations of the paired peaks (see Supplementary Data). ^13^C NMR spectra also displayed duplicated signals for certain carbon atoms, which confirmed the presence of a mixture of conformational isomers.

**Table 1.** *In vitro* cytotoxicity of compounds **2a**–**g**, **3**, **4a**–**j**, and cabozantinib against HCT116 cell line.

Compound	IC_50_ (µM)	Compound	IC_50_ (µM)
2a	15.11 ± 0.80	4b	0.734 ± 0.04
2b	16.02 ± 0.85	4c	31.82 ± 1.68
2c	4.692 ± 0.25	4d	10.83 ± 0.57
2d	47.52 ± 2.51	4e	1.408 ± 0.07
2e	37.99 ± 2.01	4f	44.1 ± 2.33
2f	5.401 ± 0.29	4g	3.697 ± 0.20
2g	12.5 ± 0.66	4h	6.989 ± 0.37
3	24.29 ± 1.28	4i	9.204 ± 0.49
4a	36.94 ± 1.95	4j	18.89 ± 1.00
Cabozantinib	16.350 ± 0.86		

**Figure 6.**
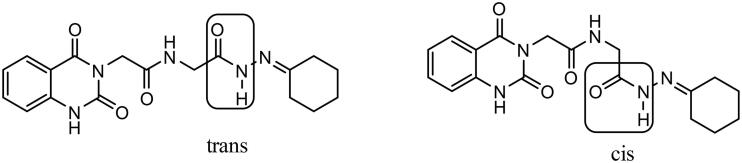
*Cis*/*trans* amide conformers of hydrazone **4e**.

The reaction of carbohydrazide **3** with cyclohexanone afforded **4e**. IR spectrum of **4e** showed characteristic stretching bands for NH’s at 3256 and 3210 cm^−1^, for C═O’s at 1738 and 1647 cm^−1^ and for C═N at 1540 cm^−1^. In ^1^H NMR spectrum of hydrazone **4e** in DMSO-*d*_6_, the NHCH_2_ appeared as two doublets with similar intensities at *δ* 3.84 and 4.15 ppm for CH_2_ protons and two triplets with similar intensities at *δ* 8.32 and 8.51 ppm for NH proton. Furthermore, C(O)–NH–N proton appeared as two singlets at *δ* 10.14 and 10.46 ppm also with similar intensities. We might conclude that the hydrazone **4e** solution in DMSO is present in the form of *cis*/*trans* amide conformers in 1:1 ratio due to rotation across the amide C(O)–NH single bond (Figure 6).

Refluxing of carbohydrazide **3** with isatin resulted in the formation of hydrazone **4f**. The ^1^H NMR spectrum of this hydrazone in DMSO-*d*_6_ showed that some protons exhibit two sets of signals for each proton (NCH_2_C(O)NH, α CH_2_, C(O)–NH–N). The downfield shift of the two peaks related to the NH–N proton (*δ* 12.55 and 13.26 ppm) indicates the presence of intramolecular hydrogen bonding stabilising the *Z* geometrical isomer. Thus, it is more likely that compound **4f** is found as an equilibrium mixture of *cis*/*trans* conformers of the *Z* geometrical isomer due to rotation across the amide CO–NH single bond, and the ratio of *cis* to *trans* conformers is 7:4, respectively.^52^^,^^53^

The compound **4g** was prepared via the Knorr pyrazole synthesis by treatment of hydrazide **3** with acetylacetone as a β-diketone. The ^1^HNMR spectrum displayed singlet signals at *δ* 1.77, 1.96, and 6.40 ppm indicating the presence of two methyl groups and one methine group, beside the disappearance of NH and NH_2_ signals related to the hydrazide **3**. ^13^C NMR spectrum was also used to confirm the structure where the two CH_3_ carbons appeared at 16.32 and 26.38 ppm.

The compound **4h** was prepared by heating carbohydrazide **3** with carbon disulphide in pyridine. IR spectrum showed absorption bands at 3304 and 3214 attributed to NH’s, at 1714 and 1661 attributed to C═O’s, and at 1140 attributed to C═S. The ^1^H NMR spectrum showed the disappearance of NH_2_ signal of hydrazide **3** and the appearance of a new characteristic signal at *δ* 10.98 ppm related to NHCS proton of compound **4h**.

The reaction of hydrazide **3** with phenyl isothiocyanate yielded **4i**. IR spectrum showed bands for NH’s at 3444, 3285, and 3201 cm^–^,^1^ for C═O’s at 1735, 1704, and 1668 cm^–^,^1^ and for C═S at 1267 cm^−1^. The ^1^H NMR spectrum indicated a multiplet for aromatic protons (9H) at *δ* 7.06–7.95 ppm, besides the disappearance of NH_2_ signal of hydrazide **3** and the appearance of new characteristic signals at *δ* 9.31, 9.74, and 10.21 ppm related to the new NH groups.

Heating the thiosemicarbazide **4i** in NaOH solution under gentle reflux leads to the formation of **4j**. IR spectrum showed bands for NH’s at 3272 and 3200 cm^−1^, for C═O’s at 1718 and 1658 cm^−1^, and for C═S at 1210 cm^−1^. The ^1^H NMR spectrum showed the disappearance of the signals of NH groups of the thiosemicarbazide and the appearance of a new characteristic signal at *δ* 13.86 ppm related to the NH proton of the triazole ring.

### Biology

#### *In vitro* antiproliferative activity against HCT-116

The target compounds were evaluated for their anticancer activity against HCT-116 colon cancer cell line in which both c-Met and VEGFR-2 are overexpressed.^54^^,^^55^ MTT assay was carried out to test the effect of different concentrations of the target compounds on HCT-116 cell line using DMSO as a negative control whereas cabozantinib was used as positive control. The half maximal inhibitory concentration (IC_50_) was calculated for each compound (Table 1). Most target compounds exhibited significant anticancer activities. Amongst all compounds, compound **4b** exhibited promising inhibition activity with IC_50_ less than 1 µM. Eleven out of 18 compounds namely, **2a**, **2b**, **2c**, **2f**, **2g**, **4b**, **4d**, **4e**, **4g**, **4h**, and **4i** displayed remarkable anticancer activity (IC_50_=0.734–16.02 µM) and they were more superior than cabozantinib. On the other hand, compound **4j** (IC_50_=18.89 µM) showed comparable anticancer activities to cabozantinib. Compounds **2d**, **2e**, **3**, **4a**, **4c**, and **4f** showed noticeable anticancer activity (IC_50_=24.29–47.52 µM).

#### *In vitro* toxicity against normal cells

To scrutinise the safety of target compounds on normal cells, compounds **4b** and **4e** were selected to evaluate their cytotoxic activity against WI38 normal cell line (Table 2). Both compounds showed less toxicity to WI38 normal cell line compared to HCT 116 colon cancer cell line. Compounds **4b** and **4e** exhibited cytotoxic activity against HCT-116 colon cancer cells 25 and 7.5 times more than WI38 normal cells, respectively.

**Table 2.** *In vitro* cytotoxicity of compounds **4b** and **4e** against WI38 normal cell line.

Compound	IC_50_ (µM)
**4b**	18.45 ± 1.09
**4e**	10.55 ± 0.62
Cabozantinib	44.71 ± 2.65

#### *In vitro* activity against c-Met and VEGFR-2 tyrosine kinases

**Figure 7.**
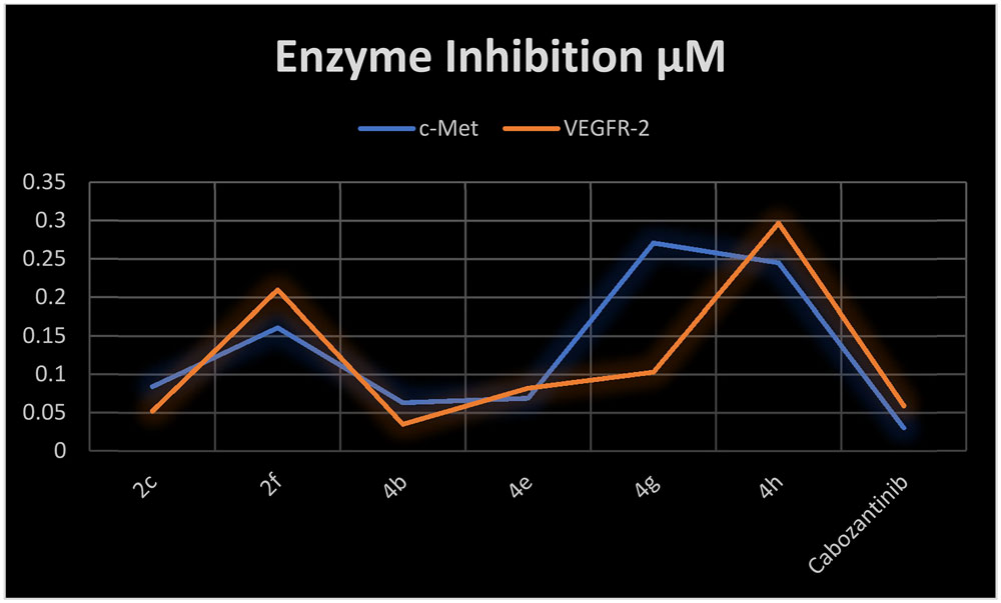
Correlation of the inhibitory activity of c-Met enzyme and VEGFR-2 enzyme.

As our rationale is to target c-Met/VEGFR-2 TKs, herein, we selected six 3-substituted quinazoline-2,4(1*H*,3*H*)-dione derivatives for evaluation of their inhibitory activity against both c-Met and VEGFR-2 enzymes.^8^ Cabozantinib was used as a positive control. The results in Table 3 showed that all scrutinised compounds have dual c-Met/VEGFR-2 inhibitory activity in the nanomolar range (IC_50_ range 0.035–0.297 µM). Three compounds namely, **2c**, **4b**, and **4e** showed substantial amount of inhibition for c-Met enzyme (IC_50_ range 0.063–0.084 µM). Interestingly, to agree with the c-Met inhibition results, compounds **2c**, **4b**, and **4e** were found to be the most potent agents against VEGFR-2 enzyme (IC_50_ range 0.035–0.082 µM) (Figure 7). This correlation between the inhibitory activity of target compounds against c-Met and VEGFR-2 TKs is in consonance with the shared pharmacophoric requirements for inhibition of the two TKs. Compared to cabozantinib, all target compounds showed less inhibitory activity against c-Met enzyme. On the other hand, compound **4b** (IC_50_=0.035 µM) exhibited superior inhibition than cabozantinib against VEFGR-2. Additionally, compound **2c** (IC_50_=0.052 µM) was equipotent to cabozantinib.

**Table 3.** Inhibitory activity of selected compounds against c-Met and VEGFR-2.

Compound	c-Met IC_50_ (μM)	VEGFR-2 IC_50_ (μM)
**2c**	0.084 ± 0.004	0.052 ± 0.002
**2f**	0.161 ± 0.007	0.210 ± 0.009
**4b**	0.063 ± 0.003	0.035 ± 0.002
**4e**	0.069 ± 0.003	0.082 ± 0.004
**4g**	0.271 ± 0.012	0.103 ± 0.005
**4h**	0.245 ± 0.011	0.297 ± 0.013
Cabozantinib	0.03 ± 0.002	0.059 ± 0.003

#### Apoptosis assay

**Figure 8.**
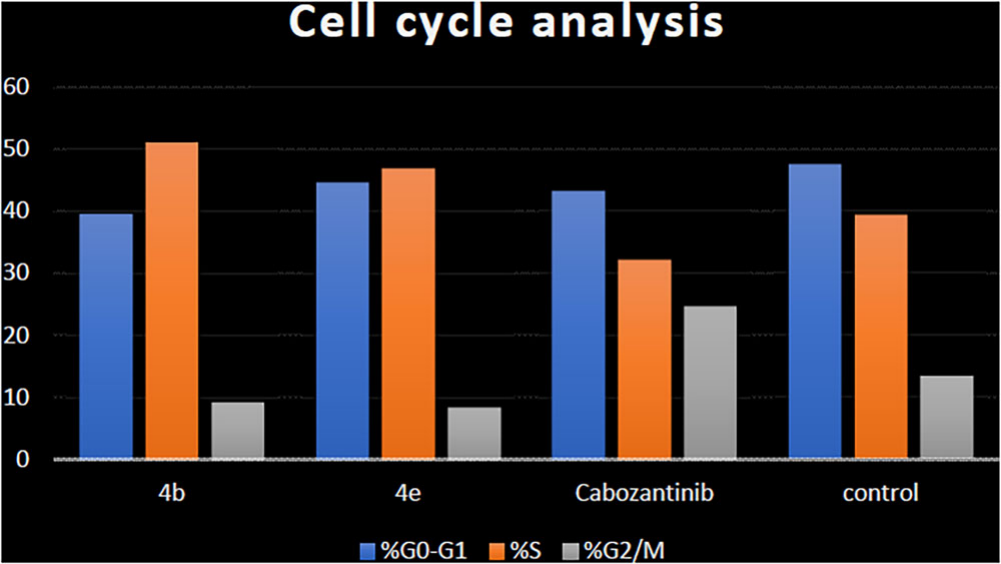
Cell cycle analysis in HCT-116 colon cancer cell line treated with compounds **4b** and **4e**.

Aiming to further study the mechanism of the target compounds, the cell cycle arrest experiment of **4b** and **4e** blocking the cell cycle of HCT-116 was carried out using Annexin V-FITC/PI staining (Table 4 and Figure 8).^56^ HCT-116 colon cancer cells were treated with target compounds **4b**, **4e**, and cabozantinib at their IC_50_ concentrations. Compared to the control, the percentage of HCT-116 cells in the S process increased to 51.07% and 46.89% after treatment with **4b** and **4e**, respectively.

**Table 4.** Cell cycle analysis in HCT-116 colon cancer cell line treated with compounds **4b** and **4e**.

Compound	%G0–G1	%S	%G2/M
**4b**	39.59	51.07	9.34
**4e**	44.60	46.89	8.51
Cabozantinib	43.12	32.16	24.72
Control	47.41	39.28	13.31

**Figure 9.**
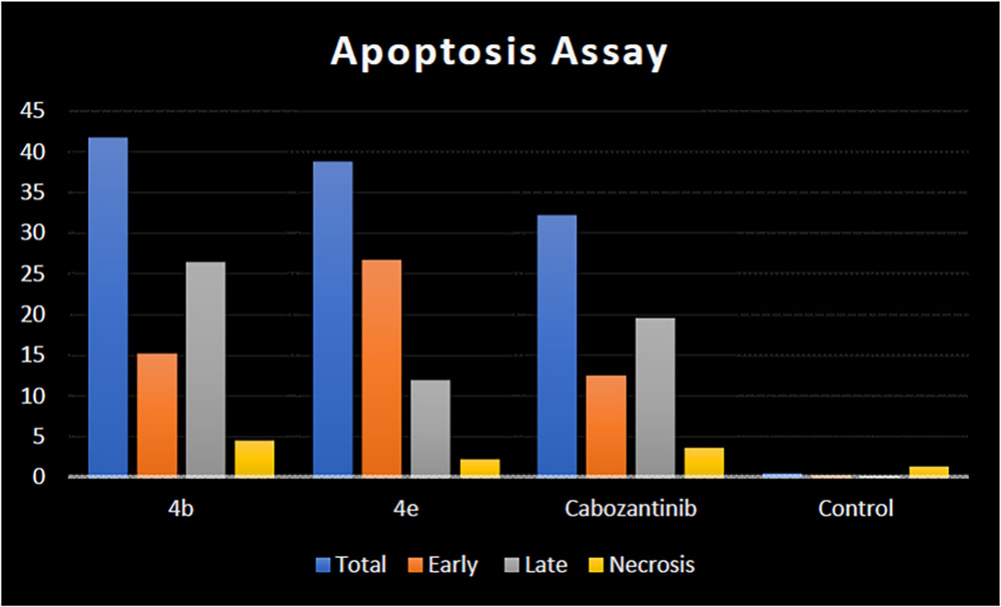
Apoptosis induction analysis using Annexin-V/propidium iodide (PI) staining assay.

**Figure 10.**
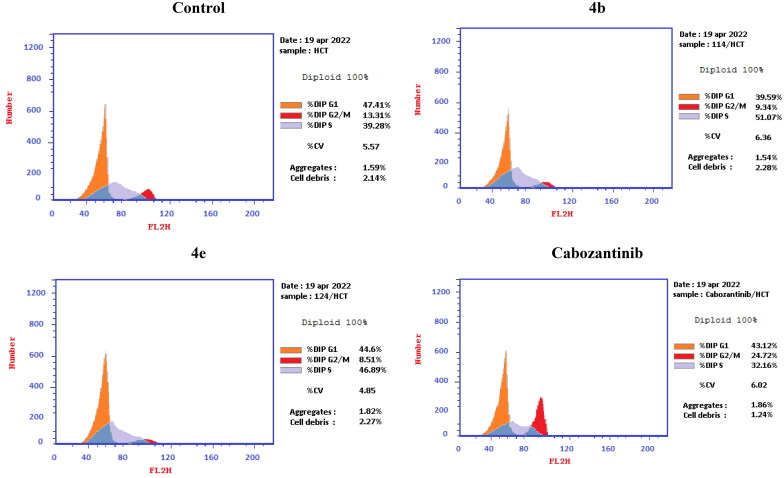
Representative cytograms of apoptotic HCT-116 cells induced by **4b** and **4e** compared to cabozantinib for 24 h.

**Figure 11.**
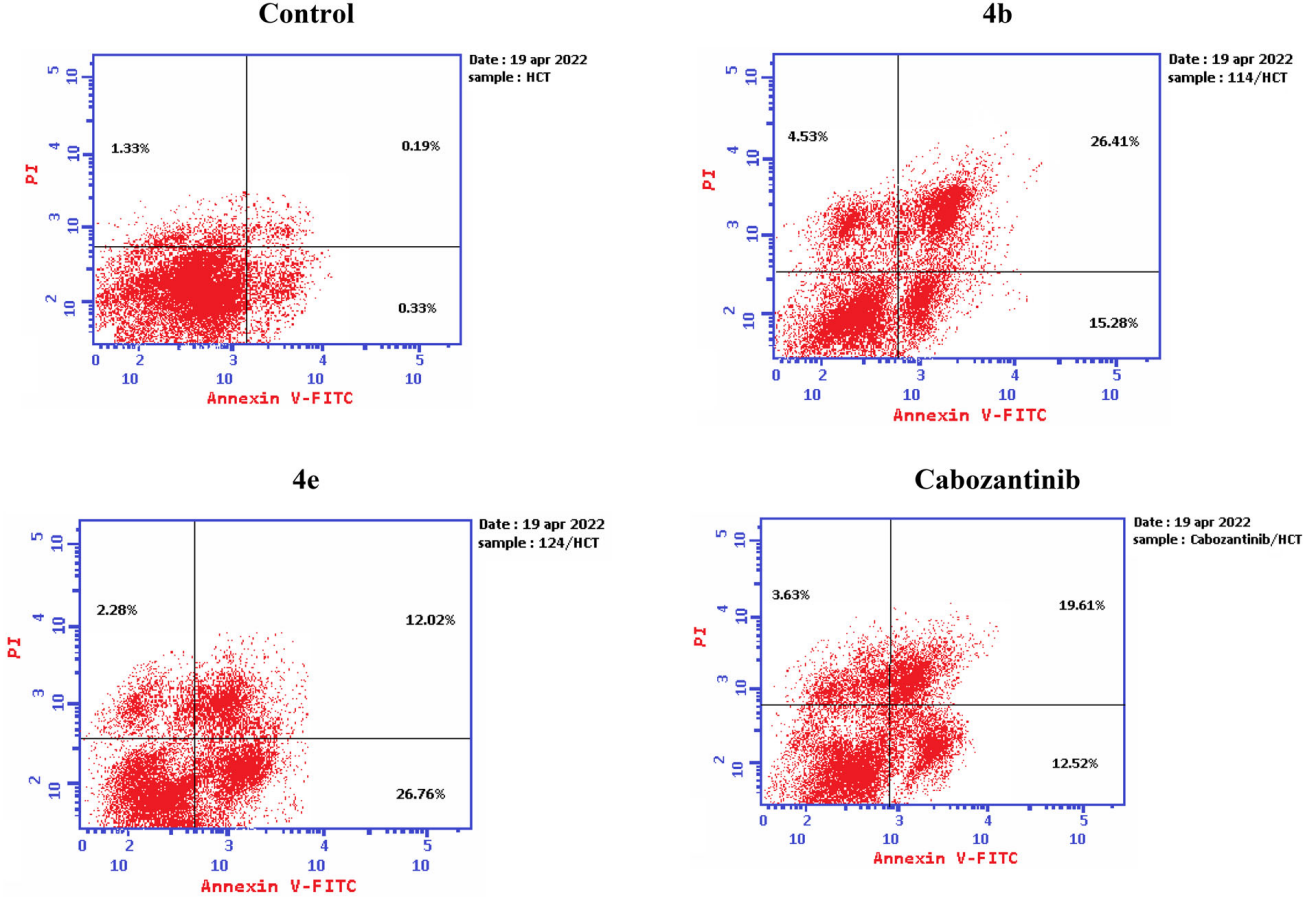
Representative cytograms of apoptotic HCT-116 cells induced by **4b** and **4e** compared to cabozantinib for 24 h.

The mechanism of HCT-116 apoptosis induced by compounds **4b**, **4e**, and cabozantinib was further scrutinised by annexin V/PI staining (Table 5).^56^ Compared to the control group, the number of both early and late apoptotic cells of target compounds **4b** and **4e** was increased. The number of early apoptotic cells of **4b** and **4e** (15.28 and 26.76%, respectively) was higher than that of cabozantinib. In addition, the number of late apoptotic and death cells of **4b** (26.41%) was higher than that of cabozantinib. It can be concluded that the synthesised compounds **4b** and **4e** can induce apoptosis of HCT-116 cells more efficiently than cabozantinib (Figures 9–11).

**Table 5.** Apoptosis induction analysis for compounds **4b**, **4e**, and cabozantinib.

Compound	Apoptosis			%Necrosis
	%Total	%Early	%Late	
**4b**	41.69	15.28	26.41	4.53
**4e**	38.78	26.76	12.02	2.28
Cabozantinib	32.13	12.52	19.61	3.63
Control HCT-116	0.52	0.33	0.19	1.33

### *In silico* studies

#### Molecular modelling studies

**Figure 12.**
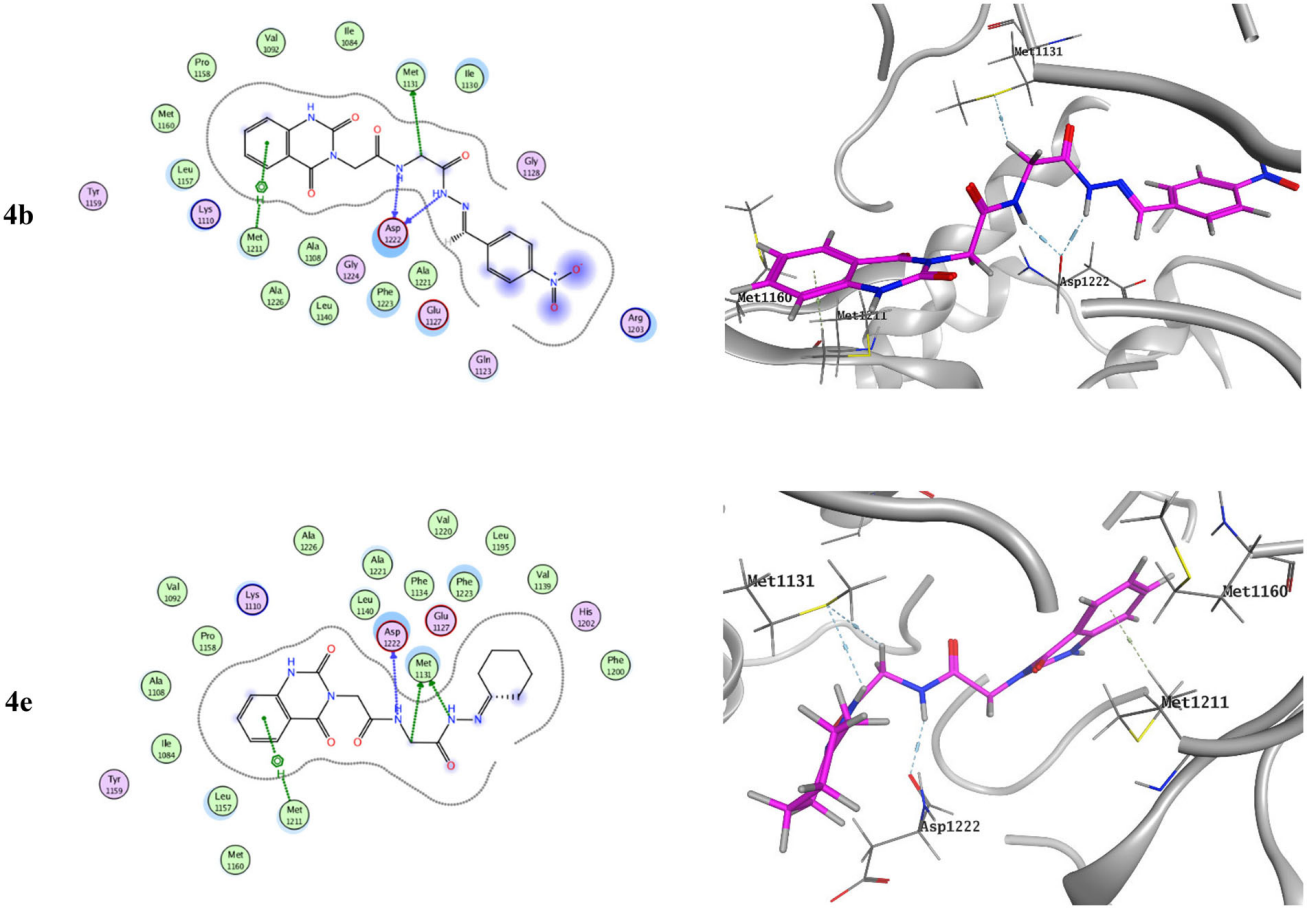
Docking pose of compound **4b** and **4e** with c-Met TK (PDB: 3lq8).

**Figure 13.**
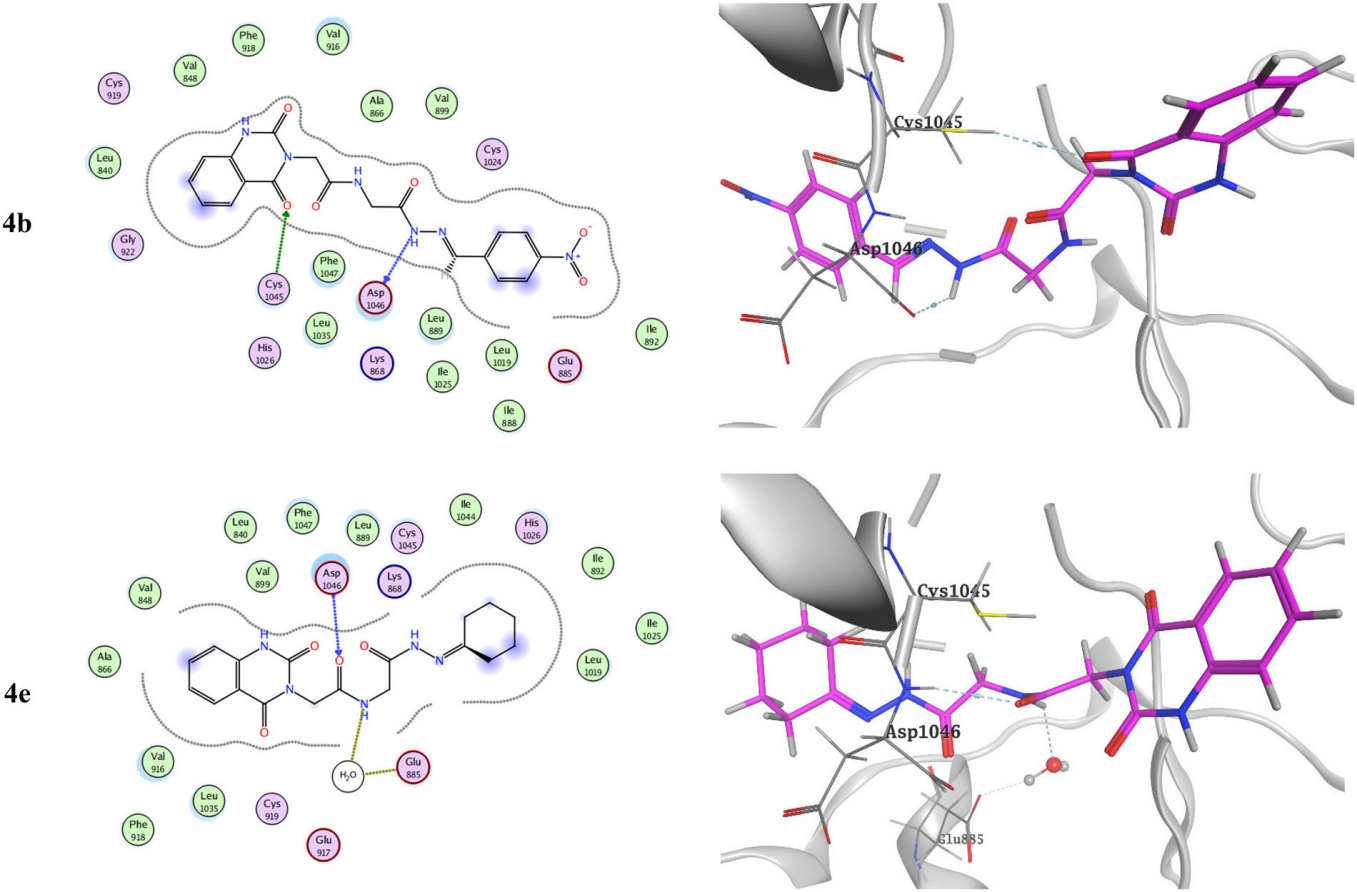
Docking pose of compound **4b** and **4e** with VEGFR-2 TK (PDB: 4asd).

Molecular docking of the target compounds was carried out in the active site of both c-Met (PDB: 3lq8) and VEGFR-2 (PDB: 4asd) TKs^8^^,^^19^. Figure 12 represents docking styles of compounds **4b** and **4e** in the active site of c-Met TK. Compound **4b** showed dual HB with highly conserved residue Asp1222 (2.64 and 2.84 Å) in the HB region as well as hydrophobic interactions with Met1131 and with Met1211 in the hinge region. Compound **4e** showed HB with Asp1222 (2.88 Å) and Met1131 (3.42 Å) in addition to hydrophobic interactions with Met1131 and Met1211. On the other hand, docking poses of compounds **4b** and **4e** in the active site of VEGFR-2 TK are shown in Figure 13. Compound **4b** showed HB with Cys1045 (3.73 Å) and with a highly conserved residue Asp1046 (3.13 Å) in the HB region. Compound **4e** showed HB with Glu885 (H_2_O 3.28 Å) and Asp1046 (3.20 Å).^8^^,^^56^

#### *In silico* prediction of physicochemical and pharmacokinetic properties

Many drug candidates failed to reach the clinic because of their improper pharmacokinetics and physicochemical properties. Consequently, *in silico* prediction of pharmacokinetic and physicochemical parameters of target compounds was carried out using SwissADME website (see Supplementary Data)^8^. SwissADME provides different reported filters to estimate drug likeness of tested compounds namely, Lipinski, Ghose, Veber, Egan, Muegge as well as bioavailability score. All target compounds showed zero or only one violation regarding all applied drug likeness filters. All target compounds have no Lipinski violation except compounds **4b** and **4f** which have only one violation just like cabozantinib. The Abbott Bioavailability Score of all tested compounds is equal to that of cabozantinib.

All target compounds have enhanced water solubility than cabozantinib. Like cabozantinib, most synthesised compounds were predicted to have high GI absorption (see Supplementary Data). Compared to cabozantinib, tested compounds were predicted not to be substrates for CYP2C19, CYP2C9, CYP2D6, and CYP3A4 that decrease the probability of drug–drug interactions.

**Figure 14.**
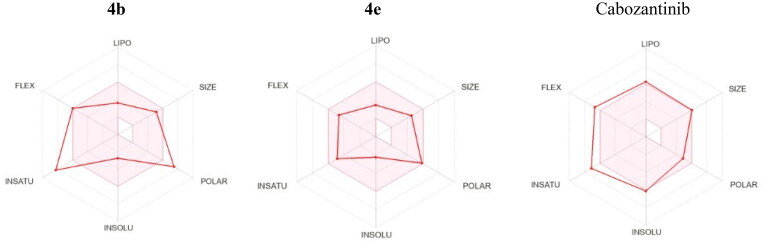
Bioavailability Radar for **4b**, **4e**, and cabozantinib.

Another tool to assess the oral bioavailability of small molecules is the Bioavailability Radar (Figure 14). It is a hexagon with specific physicochemical property at each vertex and the central pink region represents the optimum physicochemical space for good oral bioavailability. Compound **4e** showed good fit to all six physicochemical parameters and it is predicted to have good oral bioavailability. Compound **4b** exhibited deviation in both **(**Fraction Csp3 = 11) and (TPSA = 171.24 Å^2^) which expressed insaturation and polarity. In addition, cabozantinib showed deviation in both flexibility and insaturation as it has (10 rotatable bonds) and (Fraction Csp3 = 0.18).

**Figure 15.**
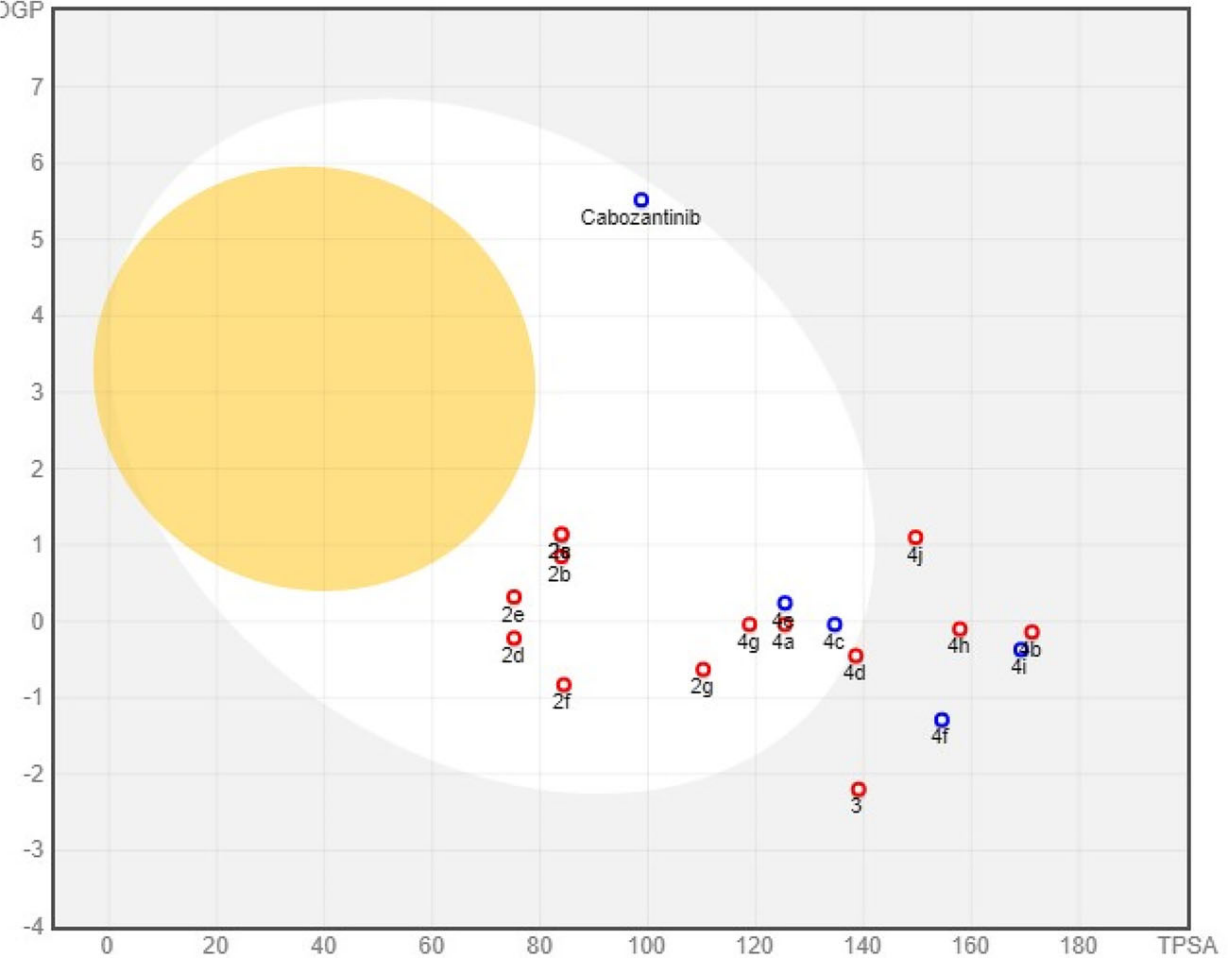
BOILED-Egg model of target compounds and cabozantinib.

BOILED-Egg (The Brain Or IntestinaL EstimateD permeation) model is a robust method that carefully prognosticates both GI absorption and BBB accessibility by estimating both the lipophilicity (expressed in WLOGP) and polarity (expressed in TPSA) of target compounds (Figure 15). Like cabozantinib, compounds **2a**–**g**, **4a**, **4c**–**e**, and **4g** showed a high GI absorption while compounds **3**, **4b**, **4f**, and **4h**–**j** showed low gastrointestinal absorption. The high GI absorption of some synthesised compounds is due to reasonable balance between their lipophilicity (WLOGP –0.83 to 1.14) and their polarity (TPSA 75.17–138.56 Å^2^). Moreover, all tested compounds as well as cabozantinib do not pass BBB confirming their good CNS safety profile.

## Conclusions

The current study was aimed at identifying novel drug-like molecules as anticancer drug candidates using *in silico* and *in vitro* techniques. Novel series of 3-substituted quinazoline-2,4(1*H*,3*H*)-dione derivatives of quinazolinone scaffold were synthesised and their chemical structures were confirmed by elemental and spectroscopic analyses. New synthetic methods were introduced for reported derivatives of 3-substituted quinazoline-2,4(1*H*,3*H*)-dione **2a**–**g**, in addition to the preparation of some new derivatives namely, **3** and **4a**–**j**. Compounds **2c**, **4b**, and **4e** showed remarkable dual inhibition for both c-Met and VEGFR-2 TKs. Designed compounds exhibited binding mode with target proteins like that of cabozantinib. Compounds **4b** and **4e** showed HB with highly conserved residue Asp1222 in the HB region of c-Met TK. For VEGFR-2 TK, compound **4b** showed HB with a highly conserved residue Asp1046 in the HB region. Compound **4e** showed HB with Glu885 and Asp1046. Moreover, *in silico* prediction of pharmacokinetic and physicochemical parameters of target compounds was carried out using SwissADME website. The quinazoline-2,4(1*H*,3*H*)-dione derivatives are promising antiproliferative candidates that require further optimisation.

## Experimental

### Chemistry

All reactions were followed by thin layer chromatography (TLC). Melting points were measured using an electrothermal melting apparatus and are uncorrected. IR spectra were recorded on a Shimadzu FT-IR 8101 PC infra-red spectrophotometer (Kyoto, Japan). ^1^H and ^13^C NMR spectra were recorded on a BRUKER 400 MHz instrument (400.2 MHz for ^1^H and 100.64 MHz for ^13^C). Chemical shifts are expressed in parts per million (ppm) units using tetramethylsilane (TMS) as an internal reference. Mass spectra were recorded on a Shimadzu GC-MS QP-2010 Plus EX mass spectrometer (Kyoto, Japan) at 70 eV. Elemental analyses were carried out at the Microanalytical Centre of Cairo University, Giza, Egypt.

#### General procedure for synthesis of amides 2a–f

To a cold solution of azide 1 (0.49 g, 2 mmol) in ethyl acetate (15 ml), a solution of the corresponding amine derivative (2 mmol) in ethyl acetate (5 ml) was added dropwise with stirring. The reaction mixture was stirred at 0–5 °C for 6–14 h, then kept at room temperature overnight. The solid product was collected by filtration and crystallised from the appropriate solvent.

##### 2-(2,4-Dioxo-1,4-dihydroquinazolin-3(2H)-yl)-N-phenylacetamide (2a)

Crystallisation from acetic acid yielded 0.52 g (88%) of compound **2a** as white crystals; m.p. over 300 °C (reported 294–296 °C)^36^. FT-IR (KBr, *υ*, cm^−1^): 3204, 3136 (NH’s), 1737, 1636 (C═O’s). ^1^H NMR (DMSO-*d*_6_), *δ*, ppm: 4.72 (s, 2H, NCH_2_), 7.06 (t, 1H, ArH), 7.25 (m, 2H, ArH_quinazoline_), 7.32 (m, 2H, ArH), 7.57 (m, 2H, ArH), 7.71 (t, 1H, ArH_quinazoline_), 7.96 (d, 1H, ArH_quinazoline_), 10.31 (s, 1H, CONH), and 11.61 (s, 1H, quinazoline NH). MS *m/z* (%): 295 [M]^+^ (4.36%). Anal. Calcd. for C_16_H_13_N_3_O_3_: C, 65.08; H, 4.44; N, 14.23%. Found C, 65.16; H, 4.62; N, 14.12%.

##### N-Benzyl-2-(2,4-dioxo-1,4-dihydroquinazolin-3(2H)-yl)acetamide (2b)

Crystallisation from acetic acid yielded 0.56 g (90.6%) of compound **2b** as white crystals; m.p. 298–299 °C (reported 294–296 °C)^36^. FT-IR (KBr, *υ*, cm^−1^): 3199 (NH’s), 2954, 2874 (aliphatic CH’s), 1735, 1640 (C═O’s). ^1^H NMR (DMSO-*d*_6_), *δ*, ppm: 4.31 (d, *J*= 6 Hz, 2H, NHCH_2_), 4.56 (s, 2H, NCH_2_), 7.20–7.35 (m, 7H, ArH), 7.69 (t, *J*= 7.6 Hz, 1H, ArH_quinazoline_), 7.96 (d, *J*= 7.6 Hz, 1H, ArH_quinazoline_), 8.66 (t, *J*= 6 Hz, 1H, NHCH_2_), and 11.54 (s, 1H, quinazoline NH). MS *m/z* (%): 309 [M]^+^ (9.03%) and 310 [M + 1]^+^ (2.06%). Anal. Calcd. for C_17_H_15_N_3_O_3_: C, 66.01; H, 4.89; N, 13.58%. Found C, 66.23; H, 5.05; N, 13.44%.

#### Another method for synthesis of compound 2b

A mixture of azide **1** (0.49 g, 2 mmol) and benzylamine (0.22 ml, 2 mmol) in dry benzene (15 ml) was refluxed for 4 h, then allowed to cool. The solid product was filtered off, dried, and crystallised from acetic acid to give 0.51 g (82.5%) of the target product **2b** as white crystals.

##### N-Cyclohexyl-2-(2,4-dioxo-1,4-dihydroquinazolin-3(2H)-yl)acetamide (2c)

Crystallisation from AcOH/H2O yielded 0.56 g (93%) of compound **2c** as white crystals; m.p. over 300 °C (reported 308–310 °C)^36^. FT-IR (KBr, *υ*, cm^−1^): 3252, 3202 (NH’s), 2929, 2855 (aliphatic CH’s), 1734, 1642 (C═O’s). ^1^H NMR (DMSO-*d*_6_), *δ*, ppm: 1.08–1.75 (m, 10H, cyclohexyl ring 5CH_2_), 3.52 (m, 1H, cyclohexyl ring CH), 4.46 (s, 2H, NCH_2_), 7.20–7.25 (m, 2H, ArH), 7.68 (t, *J*= 7.6 Hz, 1H, ArH), 7.94 (d, *J*= 7.6 Hz, 1H, ArH), 8.01 (d, *J*= 7.6 Hz, 1H, NH), and 11.48 (s, 1H, quinazoline NH). MS *m/z* (%): 301 [M]^+^ (6.80%). Anal. Calcd. for C_16_H_19_N_3_O_3_: C, 63.77; H, 6.36; N, 13.94%. Found C, 63.95; H, 6.48; N, 14.02%.

#### Another method for synthesis of compound 2c

A mixture of azide **1** (0.49 g, 2 mmol) and cyclohexylamine (0.23 ml, 2 mmol) in dry benzene (15 ml) was refluxed for 5 h, then allowed to cool. The solid product was filtered off, dried, and crystallised from AcOH/H_2_O to give 0.47 g (78%) of the target product as white crystals.

##### 2-(2,4-Dioxo-1,4-dihydroquinazolin-3(2H)-yl)-N,N-dimethylacetamide (2d)

The solid product was collected by filtration and dried to give 0.21 g (42.5%) of the desired product; m.p. 218–220 °C. FT-IR (KBr, *υ*, cm^−1^): 3245 (NH), 1730, 1661 (C═O’s).^1^H NMR (DMSO*d*_6_), *δ*, ppm: 2.85, 3.08 (s, 6H, 2CH_3_), 4.73 (s, 2H, NCH_2_), 7.20–7.25 (m, 2H, ArH), 7.69 (t, *J*= 7.6 Hz, 1H, ArH), 7.93 (d, *J*= 6.8 Hz, 1H, ArH), and 11.54 (s, 1H, quinazoline NH). ^13^C NMR (DMSO-*d*_6_), *δ*, ppm: 35.56, 36.21, 41.94, 114.01, 115.66, 123.14, 127.86, 135.69, 139.87, 150.51, 162.22, and 166.35. MS *m/z* (%): 247 [M]^+^ (15.03%) and 248 [M + 1]^+^ (2.30%). Anal. Calcd. for C_12_H_13_N_3_O_3_: C, 58.29; H, 5.30; N, 17.00%. Found C, 58.11; H, 5.19; N, 16.91%^37^.

##### 3-(2-Oxo-2-(piperidin-1-yl)ethyl)quinazoline-2,4(1H,3H)-dione (2e)

Crystallisation from benzene yielded 0.39 g (68%) of compound **2e** as white crystals; m.p. 204 °C. FT-IR (KBr, *υ*, cm^−1^): 3193 (NH), 2934, 2857 (aliphatic CH’s), 1721, 1652 (C═O’s).^1^H NMR (CDCl_3_), *δ*, ppm: 1.61–1.70 (m, 6H, CH_2_-3, 4 and 5), 3.53 and 3.62 (2t, 4H, CH_2_-2 and 6), 4.90 (s, 2H, NCH_2_), 6.97–7.04 (m, 2H, ArH), 7.35–7.40 (td, *J*= 7.6 Hz, 1H, ArH), 7.84–7.86 (dd, *J*= 8 Hz, 1H, ArH), and 10.38 (s, 1H, quinazoline NH). MS *m/z* (%): 287 [M]^+^ (27.44%), 288 [M + 1]^+^ (6.28%). Anal. Calcd. for C_15_H_17_N_3_O_3_: C, 62.71; H, 5.96; N, 14.63%. Found C, 62.87; H, 5.80; N, 14.56%^38^.

##### 3-(2-Morpholino-2-oxoethyl)quinazoline-2,4(1H,3H)-dione (2f)

Crystallisation from benzene/ethanol yielded 0.515 g (89%) of compound **2f** as white crystals; m.p. 254–255 °C. FT-IR (KBr, *υ*, cm^−1^): ∼3430 (NH), 2907, 2874 (aliphatic CH’s), 1715, 1650 (C═O’s). ^1^H NMR (DMSO-*d*_6_), *δ*, ppm: 3.44 (t, 4H, morpholine ring 2 N-linked CH_2_), 3.64 (t, 4H, morpholine ring 2 O-linked CH_2_), 4.77 (s, 2H, NCH_2_), 7.21–7.25 (m, 2H, ArH), 7.69 (t, *J*= 7.6 Hz, 1H, ArH), 7.94 (d, *J*= 7.6 Hz, 1H, ArH), and 11.56 (s, 1H, quinazoline NH). MS *m/z* (%): 289 [M]^+^ (11.29%) and 290 [M + 1]^+^ (1.98%). Anal. Calcd. for C_14_H_15_N_3_O_4_: C, 58.13; H, 5.23; N, 14.53%. Found C, 58.03; H, 5.08; N, 14.45%^39^.

##### Ethyl [2-(2,4-dioxo-1,4-dihydroquinazolin-3(2H)-yl)acetyl]glycinate (2g)

###### Method A

A solution of glycine ethyl ester hydrochloride (0.14 g, 1 mmol) in ethyl acetate (10 ml) containing 0.2 ml of Et_3_N was added to a solution of azide **1** (0.245 g, 1 mmol) in ethyl acetate (10 ml). The mixture was stirred at 0–5 °C for 24 h, then kept at room temperature for another 24 h. The solvent was evaporated, and the residue was crystallised from benzene/ethanol to give the target product as white crystals; yield 0.15 g (49%); m.p. 228–230 °C (reported 223–225 °C)^40^.

###### Method B

A mixture of azide **1** (0.245 g, 1 mmol) and glycine ethyl ester hydrochloride (0.14 g, 1 mmol) in dry pyridine (5 ml) was refluxed for 5 h. The reaction mixture was allowed to cool, then acidified with cold dilute hydrochloric acid (1:1). The resultant precipitate was collected by filtration, dried, and crystallised from benzene/ethanol to give the target product as white crystals; yield 0.235 g (77%). FT-IR (KBr, *υ*, cm^−1^): ∼3255, 3205 (NH’s), ∼2945, 2885 (aliphatic CH’s), 1740, 1645 (C═O’s). ^1^H NMR (DMSO-*d*_6_), *δ*, ppm: 1.19 (t, *J*= 7.2 Hz, 3H, OCH_2_CH_3_), 3.86 (d, *J*= 6 Hz, 2H, NHCH_2_), 4.10 (q, *J*= 7.2 Hz, 2H, OCH_2_CH_3_), 4.56 (s, 2H, NCH_2_), 7.20–7.24 (m, 2H, ArH), 7.68 (t, *J*= 7.2 Hz, 1H, ArH), 7.94 (d, *J*= 8 Hz, 1H, ArH), 8.62 (t, *J* = 6 Hz, 1H, CONHCH_2_), and 11.53 (s, 1H, quinazoline NH). ^13^C NMR (DMSO-*d*_6_), *δ*, ppm: 14.52, 41.13, 42.78, 60.93, 114.16, 115.66, 123.08, 127.88, 135.65, 139.96, 150.47, 162.25, 167.80, and 170.15. MS *m/z* (%): 305 [M]^+^ (6.86%) and 306 [M + 1]^+^ (1.30%). Anal. Calcd. for C_14_H_15_N_3_O_5_: C, 65.08; H, 4.95; N, 13.76%. Found C, 64.90; H, 5.09; N, 13.86%.

##### 2-(2,4-Dioxo-1,4-dihydroquinazolin-3(2H)-yl)-N-(2-hydrazineyl-2-oxo-ethyl)acetamide (3)

To a solution of ethyl [2-(2,4-dioxo-1,4-dihydroquinazolin-3(2H)-yl)acetyl]glycinate **2g** (0.305 g, 1 mmol) in absolute ethanol (10 ml), 0.5 ml of hydrazine hydrate was added. The reaction mixture was refluxed for 5 h, then allowed to cool. The solid product was collected by filtration, washed with boiling ethanol and dried to yield 0.217 g (74.6%) of compound **3** as white powder; m.p. 296–298 °C. FT-IR (KBr, *υ*, cm^−1^): 3291, 3175 (NH_2_, NH’s), 2933, 2894 cm^−1^ (aliphatic CH’s), 1704, 1644 (C═O’s). ^1^H NMR (DMSO-*d*_6_), *δ*, ppm: 3.70 (d, *J*= 6 Hz, 2H, NHCH_2_), 4.26 (s, 2H, NH_2_), 4.57 (s, 2H, NCH_2_), 7.20–7.25 (m, 2H, ArH), 7.69 (t, *J*= 7.8 Hz, 1H, ArH), 7.94 (d, *J*= 7.4 Hz, 1H, ArH), 8.49 (t, *J*= 6 Hz, 1H, CONHCH_2_), 9.02 (s, 1H, NHNH_2_), and 11.55 (s, 1H, quinazoline NH). ^13^C NMR (DMSO-*d*_6_), *δ*, ppm: 41.31, 43.00, 114.18, 115.66, 123.10, 127.89, 135.65, 139.93, 150.52, 162.33, 167.63, and 168.49. MS *m/z* (%): 291 [M]^+^ (4.95%). Anal. Calcd. for C_12_H_13_N_5_O_4_: C, 49.48; H, 4.50; N, 24.04%. Found C, 49.35; H, 4.39; N, 23.94%.

#### General procedure for synthesis of arylidenes 4a–d

To a solution of hydrazide **7** (0.291 g, 1 mmol) in absolute ethanol (15 ml), aldehyde (1 mmol) and few drops of piperidine were added. The reaction mixture was refluxed for 9–14 h (determined by TLC), then allowed to cool. The formed precipitate was filtered off and crystallised from the appropriate solvent.

##### (E)-N-[2-(2-Benzylidenehydrazineyl)-2-oxoethyl]-2-(2,4-dioxo-1,4-dihy-droquinazolin-3(2H)-yl)acetamide (4a)

Crystallisation from DMF/ethanol afforded compound **4a** as white powder; yield 0.36 g (95%); m.p. 325 °C. FT-IR (KBr, *υ*, cm^−1^): 3252, 3202 (NH’s), 1735, 1690, 1644 (C═O’s), 1518 (C═N). ^1^H NMR (DMSO-*d*_6_), *δ*, ppm: 3.86, 4.30 (2d, 2H, NHCH_2_), 4.61 (s, 2H, NCH_2_), 7.21–7.25 (m, 2H, ArH), 7.42–7.44 (m, 3H, ArH), 7.68–7.69 (m, 3H, ArH), 7.95 (d, *J*= 7.6 Hz, 1H, ArH), 7.99, 8.22 (2s, 1H, N═CH), 8.45, 8.63 (2t, 1H, CONHCH_2_), 11.39, 11.52 (2s, 1H, NH–N), and 11.52 (s, 1H, quinazoline NH). ^13^C NMR (DMSO-*d*_6_), *δ*, ppm: 41.92, 42.89, 114.21, 115.66, 123.02, 127.23, 127.52, 127.87, 129.25, 130.30, 130.51, 134.54, 135.56, 139.97, 143.97, 147.37, 150.52, 162.26, 165.66, 167.54, 167.80, and 170.56. MS *m/z* (%): 379 [M]^+^ (22.86%). Anal. Calcd. for C_19_H_17_N_5_O_4_: C, 60.15; H, 4.52; N, 18.46%. Found C, 60.04; H, 4.45; N, 18.42%.

##### (E)-2-(2,4-Dioxo-1,4-dihydroquinazolin-3(2H)-yl)-N-[2-(2-[4-nitrobenz-ylidene]hydrazineyl)-2-oxoethyl]acetamide (4b)

Crystallisation from DMF/H_2_O afforded compound **4b** as yellowish white powder; yield 0.408 g (96%); m.p. over 300 °C. FT-IR (KBr, *υ*, cm^−1^): 3187, 3137 (NH’s), 1706, 1669 (C═O’s), 1563 (C═N). ^1^H NMR (DMSO-*d*_6_), *δ*, ppm: 3.91, 4.34 (2s, 2H, NHCH_2_), 4.60 (s, 2H, NCH_2_), 7.21 (m, 2H, ArH), 7.67 (t, 1H, ArH), 7.93 (m, 3H, ArH), 8.24–8.27 (m, 2H, ArH), 8.06, 8.29 (2s, 1H, N═CH), 8.50, 8.67 (2brs, CONHCH_2_), 11.52 (s, 1H, quinazoline NH), 11.73, 11.80 (2s, 1H, NH–N). ^13^C NMR (DMSO-*d*_6_), *δ*, ppm: 41.96, 42.91, 114.20, 115.66, 123.01, 124.43, 127.86, 128.18, 128.44, 130.03, 135.55, 139.96, 140.84, 141.56, 144.87, 148.18, 148.35, 150.51, 162.26, 166.17, 167.61, 167.86, and 170.99. MS *m/z* (%): 424 [M]^+^ (26.86%). Anal. Calcd. for C_19_H_16_N_6_O_6_: C, 53.78; H, 3.80; N, 19.80%. Found C, 65.67; H, 4.67; N, 14.64%.

##### (E)-2-(2,4-Dioxo-1,4-dihydroquinazolin-3(2H)-yl)-N-[2-(2-[4-methoxy-benzylidene]hydrazineyl)-2-oxoethyl]acetamide (4c)

Crystallisation from ethanol afforded compound **4c** as white powder; yield 0.393 g (96%); m.p. 290–292 °C. FT-IR (KBr, *υ*, cm^−1^): 3294, 3200 (NH’s), 1725, 1656 (C═O’s), 1570 (C═N). ^1^H NMR (DMSO-*d*_6_), *δ*, ppm: 3.80, 3.81 (2s, 3H, OCH_3_), 3.84, 4.27 (2d, 2H, NHCH_2_), 4.61 (s, 2H, NCH_2_), 6.98–7.03 (m, 2H, ArH), 7.21–7.26 (m, 2H, ArH), 7.60–7.72 (m, 3H, ArH), 7.96 (d, 1H, ArH), 7.94, 8.16 (2s, 1H, N═CH), 8.41, 8.60 (2t, CONHCH_2_), 11.22, 11.38 (2s, 1H, NH–N), and 11.52 (s, 1H, quinazoline NH). ^13^C NMR (DMSO-*d*_6_), *δ*, ppm: 41.84, 42.87, 43.02, 55.75, 114.17, 114.78, 115.66, 123.07, 123.12, 127.10, 127.15, 127.89, 128.83, 129.15, 135.62, 135.67, 139.96, 143.85, 147.19, 150.52, 150.61, 161.11, 161.31, 162.28, 162.39, 165.41, 167.53, 167.79, and 170.33. MS *m/z* (%): 409 [M]^+^ (13.29%). Anal. Calcd. for C_20_H_19_N_5_O_5_: C, 58.68; H, 4.68; N, 17.11%. Found C, 58.45; H, 4.53; N, 17.00%.

##### (E)-2-(2,4-Dioxo-1,4-dihydroquinazolin-3(2H)-yl)-N-[2-(2-[furan-2-ylmethylene]hydrazineyl)-2-oxoethyl]acetamide (4d)

Crystallisation from DMF/ethanol afforded compound **4d** as beige powder; yield 0.347 g (94%); m.p. over 300 °C. FT-IR (KBr, *υ*, cm^−1^): 3200, 3127 (NH’s), 1736, 1688, 1644 (C═O’s), and 1526 (C═N). ^1^H NMR (DMSO-*d*_6_), *δ*, ppm: 3.85, 4.23 (2d, 2H, NHCH_2_), 4.61 (s, 2H, NCH_2_), 6.61–6.64 (dd, 1H, furan ring H_X_), 6.88–6.93 (dd, 1H, furan ring H_M_), 7.21–7.26 (m, 2H, ArH), 7.69 (t, 1H, ArH), 7.81–7.84 (dd, 1H, furan ring H_A_), 7.95 (d, 1H, ArH), 7.88, 8.11 (2s, 1H, N═CH), 8.40, 8.61 (2t, CONHCH_2_), 11.32, 11.46 (s, 1H, NH–N), and 11.51 (s, 1H, quinazoline NH). MS *m/z* (%): 369 [M]^+^ (42.26%). Anal. Calcd. for C_17_H_15_N_5_O_5_: C, 55.28; H, 4.09; N, 18.96%. Found C, 55.37; H, 4.06; N, 18.91%.

##### N-[2-(2-Cyclohexylidenehydrazineyl)-2-oxoethyl]-2-(2,4-dioxo-1,4-dihydroquinazolin-3(2H)-yl)acetamide (4e)

To a solution of compound **3** (0.58 g, 2 mmol) in ethanol (20 ml), cyclohexanone (0.21 ml, 2 mmol) was added, and the reaction mixture was refluxed for 10 h. After cooling, the solid product was filtered off and dried to afford pure compound **4e** as a white powder without further purification. Yield 0.64 g (86%); m.p. 264–266 °C. FT-IR (KBr, *υ*, cm^−1^): 3256, 3210 (NH’s), 2942, 2860 (aliphatic CH’s), 1738,1647 (C═O’s), 1540 (C═N). ^1^H NMR (DMSO-*d*_6_), *δ*, ppm: 1.62–1.76 (m, 6H, 3CH_2_), 2.19–2.36 (m, 4H, 2CH_2_), 3.84, 4.15 (2d, 2H, NHCH_2_), 4.60 (s, 2H, NCH_2_), 7.21–7.30 (m, 2H, ArH), 7.69 (t, 1H, ArH), 7.94 (d, 1H, ArH), 8.32, 8.51 (2t, 1H, CONHCH_2_), 10.14, 10.46 (2s, 1H, NH), 11.51 (s, 1H, quinazoline NH). MS *m/z* (%): 371 [M]^+^ (23.14%). Anal. Calcd. for C_18_H_21_N_5_O_4_: C, 58.21; H, 5.70; N, 18.86%. Found C, 58.26; H, 5.72; N, 18.82%.

##### (Z)-2-[2,4-Dioxo-1,4-dihydroquinazolin-3(2H)-yl]-N-[2-oxo-2-(2-(2-oxoindolin-3-ylidene)hydrazineyl)ethyl]acetamide (4f)

A mixture of hydrazide **3** (0.291 g, 1 mmol) and isatin (0.147 g, 1 mmol) in acetic acid (15) was heated under reflux for 8 h, then allowed to cool. The solid product was filtered off and recrystallised from acetic acid to give compound **4f** as yellow crystals; yield 89.2%; m.p over 300 °C. FT-IR (KBr, *υ*, cm^−1^): 3328, 3228 (NH’s), 1724, 1695, 1650 (C═O’s). ^1^H NMR (DMSO-*d*_6_), *δ*, ppm: 4.01, 4.44 (2s, 2H, NHCH_2_), 4.64 (s, 2H, NCH_2_), 6.94 (d, *J*= 8 Hz, 1H, ArH), 7.09 (t, *J*= 8 Hz, 1H, ArH), 7.21–7.25 (m, 2H, ArH_quinazoline_), 7.38 (t, *J*= 8 Hz, 1H, ArH), 7.57 (d, *J*= 8 Hz, 1H, ArH), 7.69 (t, *J*= 8 Hz, 1H, ArH_quinazoline_), 7.95 (d, *J*= 8 Hz, 1H, ArH_quinazoline_), 8.60, 8.92 (2s, 1H, CONHCH_2_), 11.30 (s, 1H, indolinone NH), 11.51 (s, 1H,quinazoline NH), 12.55, 13.26 (2s, 1H, NH–N). MS *m/z* (%): 420.60 [M]^+^ (17.99%).

##### N-[2-(3,5-Dimethyl-1H-pyrazol-1-yl)-2-oxoethyl]-2-(2,4-dioxo-1,4-dihyd-roquinazolin-3(2H)-yl)acetamide (4g)

To a solution of compound **3** (0.58 g, 2 mmol) in ethanol (20 ml), acetyl acetone (0.2 ml, 2 mmol) was added in presence of few drops of piperidine, and the reaction mixture was refluxed for 16 h. After cooling, the solid product was filtered off and recrystallised from ethanol/benzene to afford compound **4g** as white powder; yield 0.62 g (87.3%); m.p. 284–286 °C. FT-IR (KBr, *υ*, cm^−1^): 3241, 3199 (NH’s), 1732, 1646 (C═O’s). ^1^H NMR (DMSO-*d*_6_), *δ*, ppm: 1.77 (s, 3H, CH_3_), 1.96 (s, 3H, CH_3_), 4.10 (d, *J*= 4 Hz, 2H, NHCH_2_), 4.60 (s, 2H, NCH_2_), 6.40 (s, 1H, CH), 7.20–7.24 (m, 2H, ArH), 7.68 (t, *J*= 8 Hz, 1H, ArH), 7.94 (d, *J*= 8 Hz, 1H, ArH), 8.32 (t, *J*= 4 Hz, 1H, CONHCH_2_), and 11.49 (s, 1H, quinazoline NH). ^13^C NMR (DMSO-*d*_6_), *δ*, ppm: 16.32, 26.38, 42.19, 42.85, 52.37, 90.82, 114.13, 115.65, 123.08, 127.88, 135.61, 139.92, 150.50, 155.68, 162.26, 166.30, and 167.46. MS *m/z* (%): 355 [M]^+^ (23.14%). Anal. Calcd. for C_17_H_17_N_5_O_4_: C, 57.46; H, 4.82; N, 19.71%. Found C, 57.34; H, 4.74; N, 19.67%.

##### 2-(2,4-Dioxo-1,4-dihydroquinazolin-3(2H)-yl)-N-[(5-thioxo-4,5-dihydro-1,3,4-oxadiazol-2-yl)methyl]acetamide (4h)

To a solution of compound **3** (0.58 g, 2 mmol) in pyridine (15 ml), 1 ml of carbon disulphide was added. The reaction mixture was refluxed for 10 h, then allowed to cool, acidified with cold dilute hydrochloric acid (1:1), and the solid formed was collected by filtration and dried to obtain the target product as grey powder without further purification; yield 0.24 g (36%); m.p. over 300 °C. FT-IR (KBr, *υ*, cm^−1^): 3304, 3214 (NH’s), 1714, 1661 (C═O’s), and 1140 (C═S). 1H NMR (DMSO-*d*_6_), *δ*, ppm: 3.86 (d, 2H, NHCH_2_), 4.59 (s, 2H, NCH2), 7.22–7.26 (m, 2H, ArH), 7.70 (t, 1H, ArH), 7.95 (d, 1H, ArH), 8.65 (t, 1H, CONHCH_2_), 10.98 (br s, 1H, NHCS), and 11.56 (s, 1H, quinazoline NH). ^13^C NMR (DMSO-*d*_6_), *δ*, ppm: 40.87, 42.89, 114.15, 115.71, 123.08, 127.86, 135.64, 139.97, 150.49, 162.29, 167.91, and 168.78. MS *m/z* (%): 333 [M]+ (20.79%). Anal. Calcd. for C_13_H_11_N_5_O_4_S: C, 46.84; H, 3.33; N, 21.01; S, 9.62%. Found C, 46.75; H, 3.28; N, 20.96; S, 9.66%.

##### 2-(2,4-Dioxo-1,4-dihydroquinazolin-3(2H)-yl)-N-[2-oxo-2-(2-[phenylcar-bamothioyl]hydrazineyl)ethyl]acetamide (4i)

To a solution of hydrazide **3** (0.58 g, 2 mmol) in absolute ethanol (20 ml), phenyl isothiocyanate (0.24 ml, 2 mmol) was added and the reaction mixture was refluxed for 15 h, then allowed to cool. The solid product was collected by filtration and crystallised from ethanol to afford compound **4i** as white powder; yield 0.71 g (83.3%); m.p. 287–290 °C. FT-IR (KBr, *υ*, cm^−1^): 3444, 3285, 3201 (NH’s), 1735, 17.0, 1668 (C═O’s), and 1267 (C═S). ^1^H NMR (DMSO-*d*_6_), *δ*, ppm: 3.82 (d, 2H, NHCH_2_), 4.64 (s, 2H, NCH_2_), 7.06–7.95 (m, 9H, ArH), 8.75 (br s, 1H, CONHCH_2_), 9.31 (br s, 1H, NH), 9.74 (br s, 1H, NH), 10.21 (s, 1H, NH), and 11.56 (s, 1H, quinazoline NH). MS *m/z* (%): 426.56 [M]^+^ (60.16%). Anal. Calcd. for C_19_H_18_N_6_O_4_S: C, 53.51; H, 4.25; N, 19.71; S, 7.52%. Found C, 53.38; H, 4.18; N, 19.62; S, 7.61%.

##### 2-(2,4-Dioxo-1,4-dihydroquinazolin-3(2H)-yl)-N-[(4-phenyl-5-thioxo-4,5-dihydro-1H-1,2,4-triazol-3-yl)methyl]acetamide (4j)

The thiosemicarbazide **4i** (0.426 g, 1 mmol) was suspended in 2% solution of NaOH (15 ml) and heated under gentle reflux for 6 h. after cooling, the solution was neutralised with dil. HCl. The resulting precipitate was filtered off, washed with distilled water, and recrystallised from ethanol to afford compound **4j** as white crystals. Yield 0.29 g (71%); m.p. over 300 °C. FT-IR (KBr, *υ*, cm^−1^): 3272, 3200 (NH’s), 1718, 1658 (C═O’s), and 1210 (C═S). ^1^H NMR (DMSO-*d*_6_), *δ*, ppm: 4.12 (d, *J*= 4 Hz, 2H, NHCH_2_), 4.42 (s, 2H, NCH_2_), 7.19–7.24 (m, 2H, ArH_quinazoline_), 7.40–7.57 (m, 5H, ArH), 7.68 (t, *J*= 8 Hz, 1H, ArH_quinazoline_), 7.94 (d, *J*= 8 Hz, 1H, ArH_quinazoline_), 8.61 (t, *J*= 4 Hz, 1H, CONHCH2), 11.50 (s, 1H, quinazoline NH), and 13.86 (s, 1H, NHCS). MS *m/z* (%): 408 [M]^+^ (39.11%). Anal. Calcd. for C_19_H_16_N_6_O_3_S: C, 55.87; H, 3.95; N, 20.58; S, 7.85%. Found C, 55.74; H, 3.78; N, 20.52; S, 7.93%.

### Biological evaluation

#### *In vitro* antiproliferative activity against normal and cancer cells

To scrutinise the cytotoxicity of the target compounds on colorectal cancer and normal cells, MTT assay was carried out against HCT-116 and WI38 cell lines, respectively.

#### *In vitro* activity against c-Met and VEGFR-2

Both c-Met and VEGFR-2 assay of the target compounds **2c**, **2f**, **4b**, **4e**, **4g**, and **4h** was performed by a well-known methodology of BPS KDR Assay Kit Catalog # 40325 (BPS Bioscience, San Diego, CA) (see Supplementary Data)^8^.

#### Cell cycle analysis and apoptosis assay

The effects of both **4b** and **4e** on progress of cell cycle and induction of apoptosis in HCT-166 cells were carried out using Annexin V-FITC Apoptosis Detection Kit (BioVision Research Products, Mountain View, CA) (see Supplementary Data)^8^.

### *In silico* studies

#### Molecular modelling study

Molecular modelling of compounds **4b** and **4e** and 2D and 3D visualisation processes were carried out within c-Met and VEFGR-2 TK active sites using MOE 2014.0901 software. The co-crystal structures of c-Met (PDB: 3lq8) and VEGFR-2 (PDB: 4asd) proteins were retrieved from the Protein Data Bank (RCSB). At first, **4b** and **4e** were prepared with the standard protocol of MOE 2014. After that the structures of the two proteins were prepared by using the MOE structure preparation protocol. Validation of the molecular docking study at the active sites was carried out by redocking of the original ligands into the active sites. The RMSD values of the most stable poses were 1.0648 (foretinib) and 0.9797 (sorafenib) in c-Met and VEGFR-2 active sites, respectively. Then, compounds **4b** and **4e** were docked into the active sites using the Alpha Triangle placement method. Refinement was carried out using Forcefield and scored by London dG scoring system.

#### *In silico* prediction of physicochemical and pharmacokinetic properties

The Swiss Institute of Bioinformatics provides a freely available tools for prediction of pharmacokinetics and physicochemical properties of small molecules via URL: http://www.swissadme.ch/index.php#. Different filters were used to compute druglikeness as follows: Pfizer Lipinski (MW ≤ 500, MLOGP ≤ 4.15, N or O ≤ 10, NH or OH ≤ 5); Ghose (160 ≤ MW ≤ 480, −0.4 ≤ WLOGP ≤ 5.6, 40 ≤ MR ≤ 130, 20 ≤ atoms ≤ 70); Veber (rotatable bonds ≤ 10, TPSA ≤ 140); Egan (WLOGP ≤ 5.88, TPSA ≤ 131.6); Muegge (200 ≤ MW ≤ 600, −2 ≤ XLOGP ≤ 5, TPSA ≤ 150, rings ≤ 7, C > 4, heteroatoms > 1, rotatable bonds > 15, HBA ≤ 10, HBD ≤ 5); Abbott Bioavailability Score (probability of *F* > 10% in rat).^57^ iLOG^58^. BOILED Egg is a plot of TPSA on the *X*-axis versus WLOGP on the *Y*-axis. The white oval is the suitable TPSA/WLOGP value for the highest probability of GI absorption, and the yolk circle is the suitable TPSA/WLOGP value for the highest probability to BBB accessibility. The optimal values of physicochemical properties involved in Bioavailability Radar are as follows: size (150 g/mol < MV > 500 g/mol), polarity (20 Å^2^<TPSA > 130 Å^2^), lipophilicity (–0.7 < XLOGP3> +5.0), insolubility (0 < Log S (ESOL)>6), insaturation (0.25 < Fraction Csp3 > 1.0), and flexibility (rotatable bonds < 9)^59^.

## Supplementary Material

Supplemental Material
