# Supplementary material for: Design and biological evaluation of 3-substituted quinazoline-2,4(1H,3H)-dione derivatives as dual c-Met/VEGFR-2-TK inhibitors
Source: J Enzyme Inhib Med Chem. 2023 Mar 15;38(1):2189578. doi: 10.1080/14756366.2023.2189578 (PMC10026756; doi:10.1080/14756366.2023.2189578)
Supplement: Supplemental Material [file IENZ_A_2189578_SM4314.pdf]

Design and biological evaluation of 3-substituted quinazoline-2,4(1*H*,3*H*)-dione derivatives as dual c-Met/VEGFR-2-TK inhibitors

Abdelfattah Hassan[1*], Fawzy A. F. Mubarak[2], Ihsan A. Shehadi[3], Ahmed M. Mosallam[2], Hussain Temairk[2], Mohamed Badr[4], Aboubakr H. Abdelmonsef[2*]

[1]*Department of Medicinal Chemistry, Faculty of Pharmacy, South Valley University, Qena, Egypt*
[2]*Department of Chemistry, Faculty of Science, South Valley University, Qena, Egypt*
[3]*Department of Chemistry, Pure and Applied Chemistry Research Group, College of Sciences, University of Sharjah, Sharjah 27272, UAE*
[4]*Department of Biochemistry, Faculty of Pharmacy, Menoufia University, Menoufia, Egypt*

To whom correspondence should be addressed:

[*]Abdelfattah Hassan: abdelfattah_hassan@svu.edu.eg, Tel.: +201000760083
[*]Aboubakr H. Abdelmonsef: aboubakr.ahmed@sci.svu.edu.eg; Tel.: +201098965494

List of Figures

Figure 1: IR spectrum of compound **2b**. 1
Figure 2: ^{1}H NMR spectrum of compound **2b**. 2
Figure 3: Mass spectrum of compound **2**b. 3
Figure 4: IR spectrum of compound **2b**. 4
Figure 5: ^{1}H NMR spectrum of compound **2b**. 5
Figure 6: Mass spectrum of compound **2b**. 6
Figure 7: IR spectrum of compound **2c**. 7
Figure 8: ^{1}H NMR spectrum of compound **2c**. 8
Figure 9: Mass spectrum of compound **2c**. 9
Figure 10: IR spectrum of compound **2d**. 10
Figure 11: ^{1}H NMR spectrum of compound **2d**. 11
Figure 12: ^{13}C NMR spectrum of compound **2d**. 12
Figure 13: Mass spectrum of compound **2d**. 13
Figure 14: IR spectrum of compound **2e**. 14
Figure 15: ^{1}H NMR spectrum of compound **2e**. 15
Figure 16: Mass spectrum of compound **2e**. 16

Figure 17: IR spectrum of compound **2f**. 17
Figure 18: ^{1}H NMR spectrum of compound **2f**. 18
Figure 19: Mass spectrum of compound **2f**. 19
Figure 20: IR spectrum of compound **6g**. 20
Figure 21: ^{1}H NMR spectrum of compound **6g**. 21
Figure 22: ^{13}C NMR spectrum of compound **6g**. 22
Figure 23: Mass spectrum of compound **6g**. 23
Figure 24: IR spectrum of compound **3**. 24
Figure 25: ^{1}H NMR spectrum of compound **3**. 25
Figure 26: ^{13}C NMR spectrum of compound **3**. 26
Figure 27: Mass spectrum of compound **3**. 27
Figure 28: ^{1}H NMR spectrum of compound **4a**. 28
Figure 29: ^{13}C NMR spectrum of compound **4a**. 29
Figure 30: Mass spectrum of compound **4a**. 30
Figure 31: IR spectrum of compound **4b**. 31
Figure 32: ^{1}H NMR spectrum of compound **4b**. 32
Figure 33: ^{13}C NMR spectrum of compound **4b**. 33
Figure 34: Mass spectrum of compound **4b**. 34
Figure 35: IR spectrum of compound **4c**. 35
Figure 36: ^{1}H NMR spectrum of compound **4c**. 36
Figure 37: ^{13}C NMR spectrum of compound **4c**. 37
Figure 38: Mass spectrum of compound **4c**. 38
Figure 39: IR spectrum of compound **4d**. 39
Figure 40: ^{1}H NMR spectrum of compound **4d**. 40
Figure 41: Mass spectrum of compound **4d**. 41
Figure 42: IR spectrum of compound **4e**. 42
Figure 43: ^{1}H NMR spectrum of compound **4e**. 43
Figure 44: Mass spectrum of compound **4e**. 44
Figure 45: IR spectrum of compound **4f**. 45
Figure 46: ^{1}H NMR spectrum of compound **4f**. 46
Figure 47: Mass spectrum of compound **4f**. 47
Figure 48: ^{1}H NMR spectrum of compound **4g**. 48
Figure 49: ^{13}C NMR spectrum of compound **4g**. 49
Figure 50: Mass spectrum of compound **4g**. 50
Figure 51: IR spectrum of compound **4h**. 52
Figure 52: ^{1}H NMR spectrum of compound **4h**. 53
Figure 53: ^{13}C NMR spectrum of compound **4h**. 54
Figure 54: Mass spectrum of compound **4h**. 55
Figure 55: IR spectrum of compound **4i**. 55
Figure 56: ^{1}H NMR spectrum of compound **4i**. 56

Figure 57: Mass spectrum of compound **4i**. 57
Figure 58: IR spectrum of compound **4j**. 58
Figure 59: ^{1}H NMR spectrum of compound **4j**. 60
Figure 61: c-Met IC_{50} calculations of compounds **2c, 2f, 4b, 4e, 4g, 4h** and cabozantinib. 62
Figure 62: VEGFR-2 IC_{50} calculations of compounds **2c, 2f, 4b, 4e, 4g, 4h** and cabozantinib. 66

List of Tables

Table 1: ^{1}H NMR chemical shifts and ratio of cis/trans N-acylhydrazones **4a-d**. 60
Table 2: c-Met IC_{50} calculations of compounds **2c, 2f, 4b, 4e, 4g, 4h** and cabozantinib. 60
Table 3: VEGFR-2 IC_{50} calculations of compounds **2c, 2f, 4b, 4e, 4g, 4h** and cabozantinib. 63
Table 4: Physicochemical parameters and druglikeness of target compounds and cabozantinib. 66
Table 5: Solubility and pharmacokinetics of target compounds and cabozantinib. 67

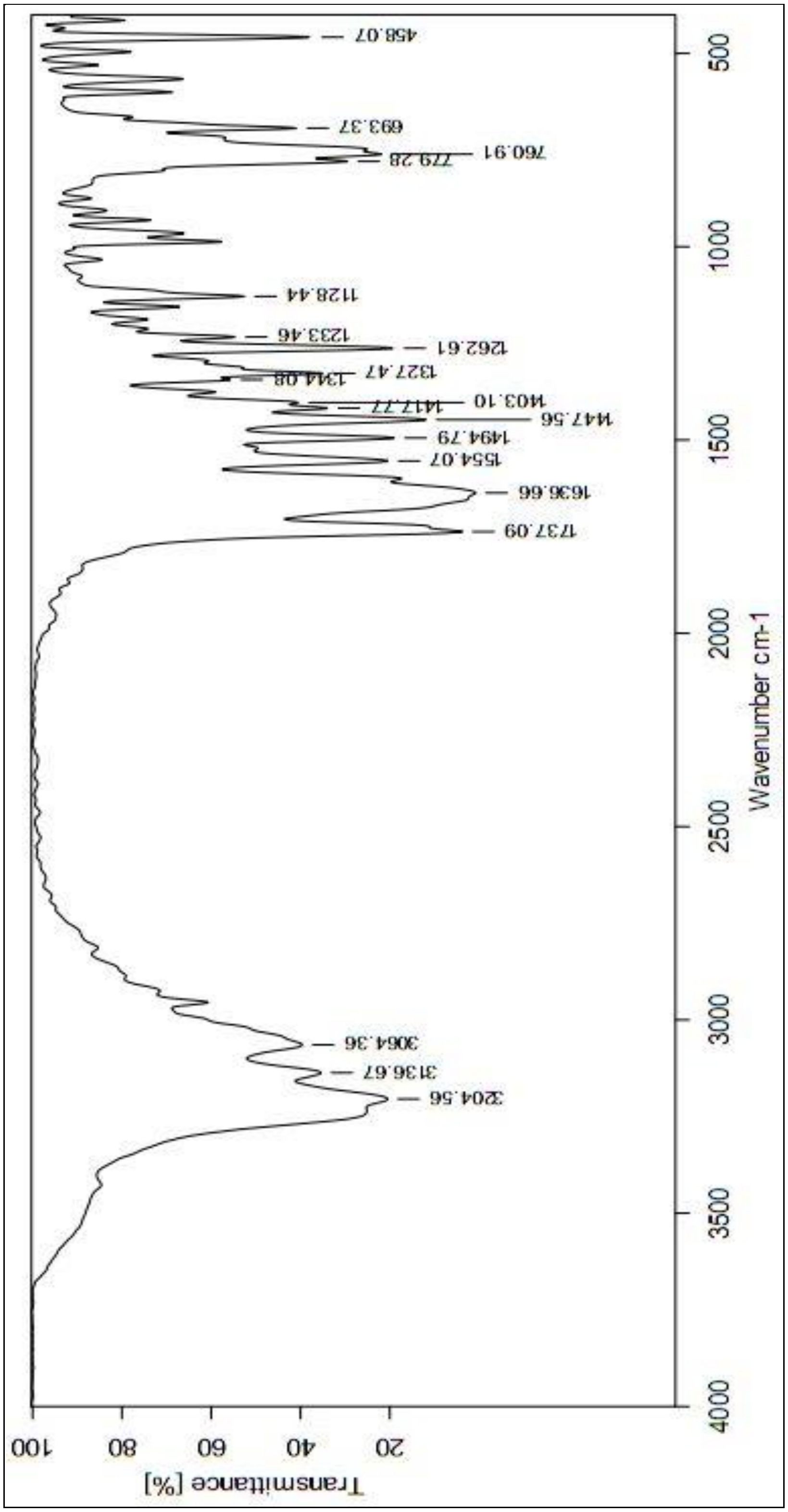

Figure 1: IR spectrum of compound **2b**.

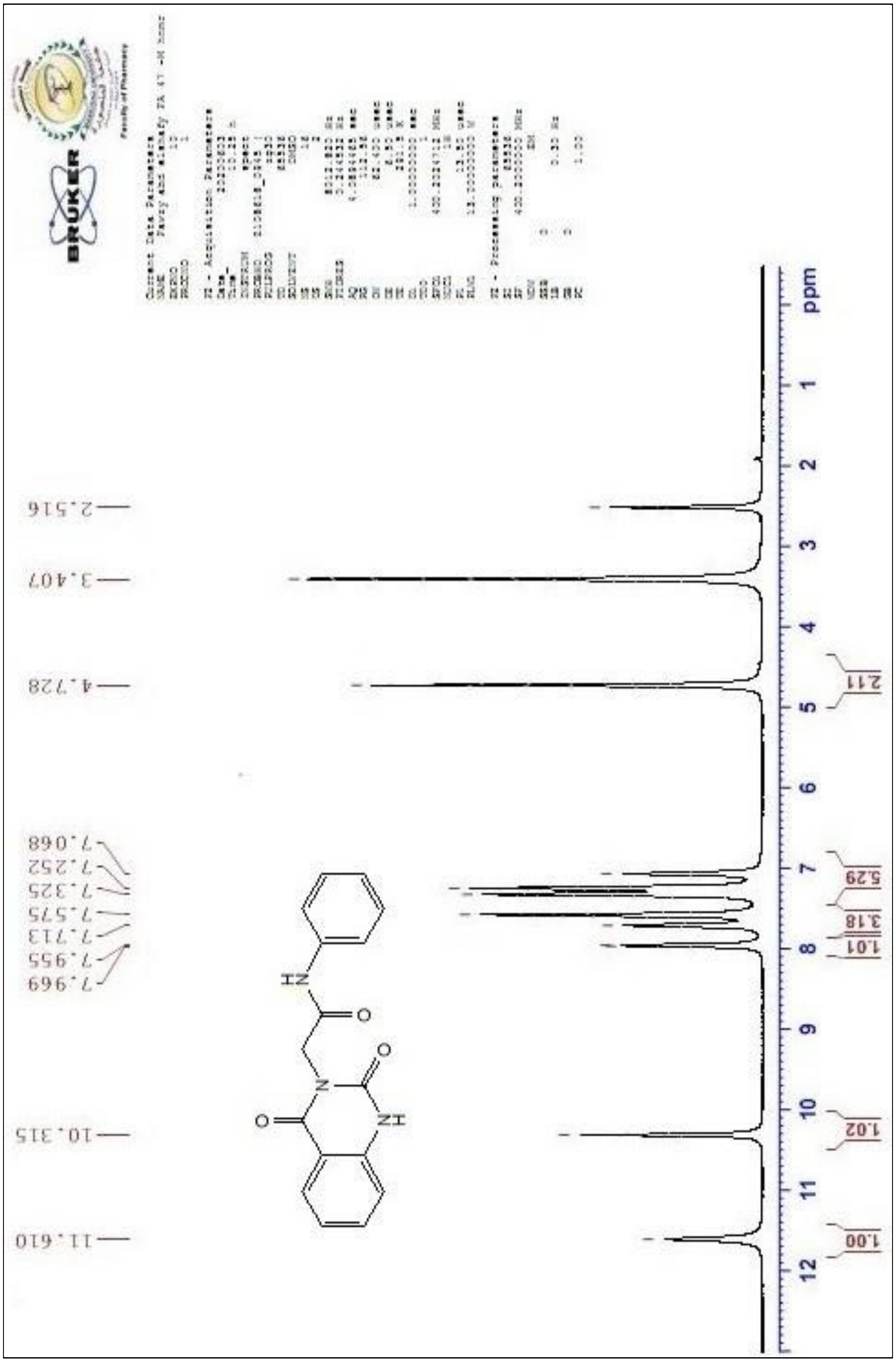

Figure 2: ^{1}H NMR spectrum of compound **2b**.

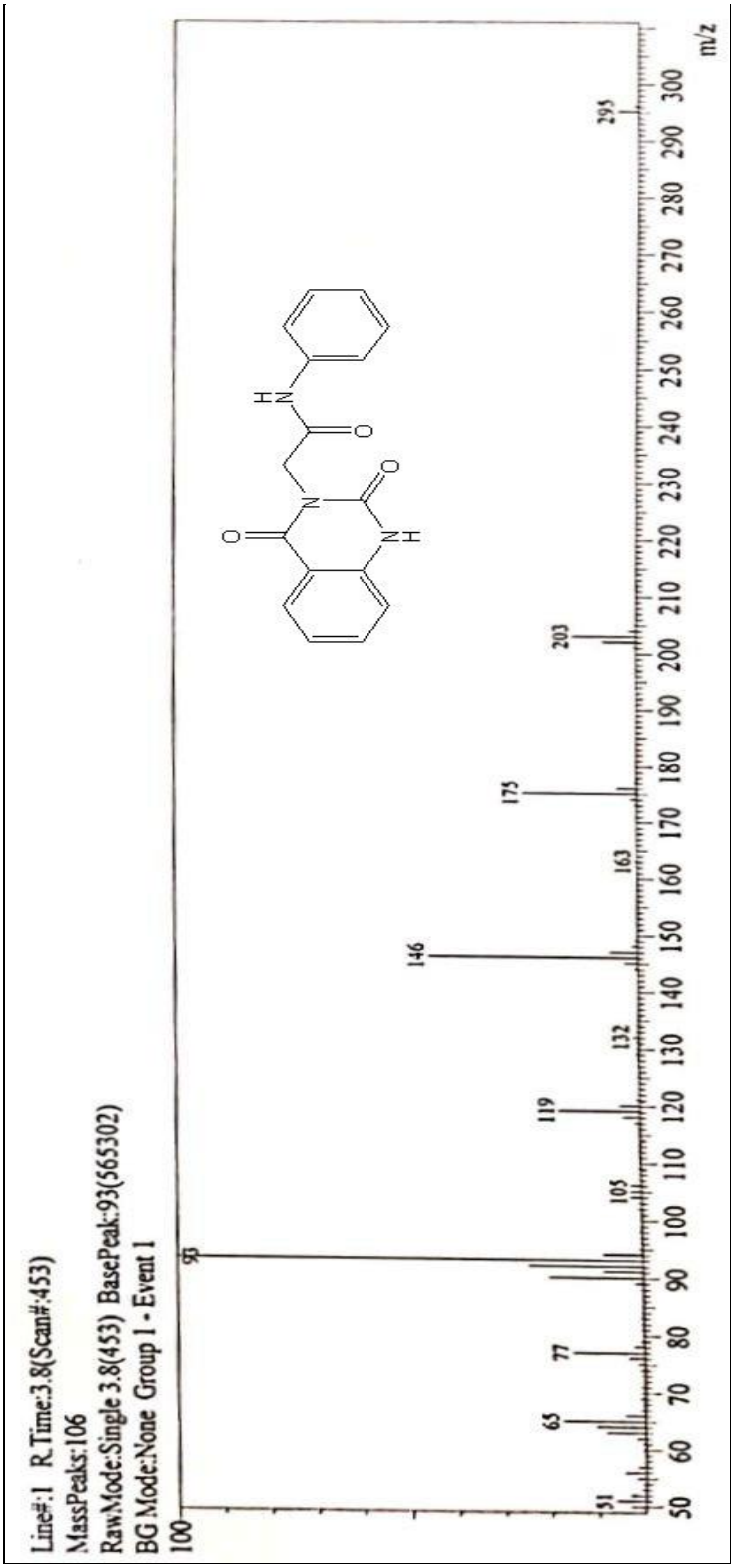

Figure 3: Mass spectrum of compound **2**b.

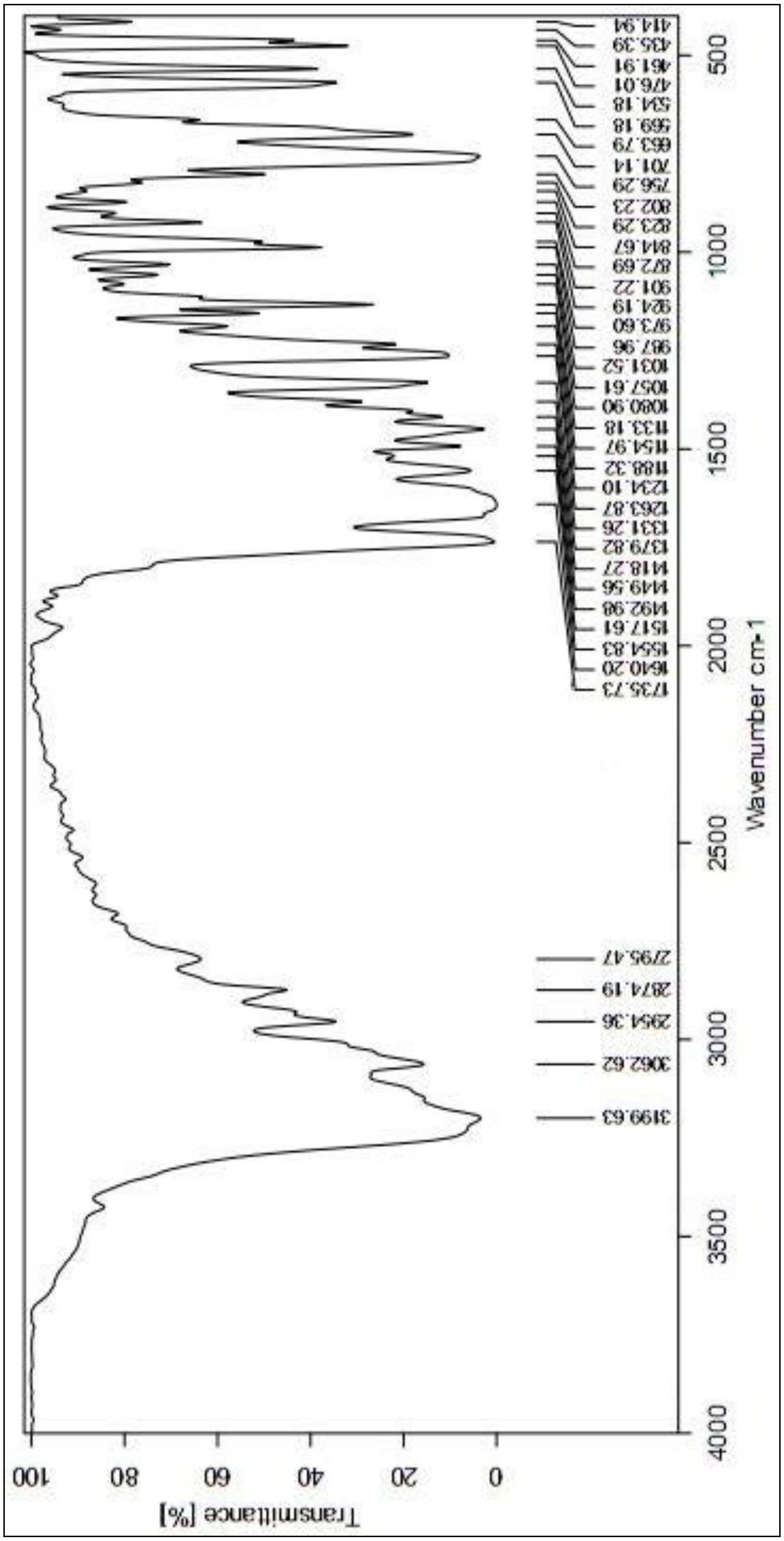

Figure 4: IR spectrum of compound **2b**.

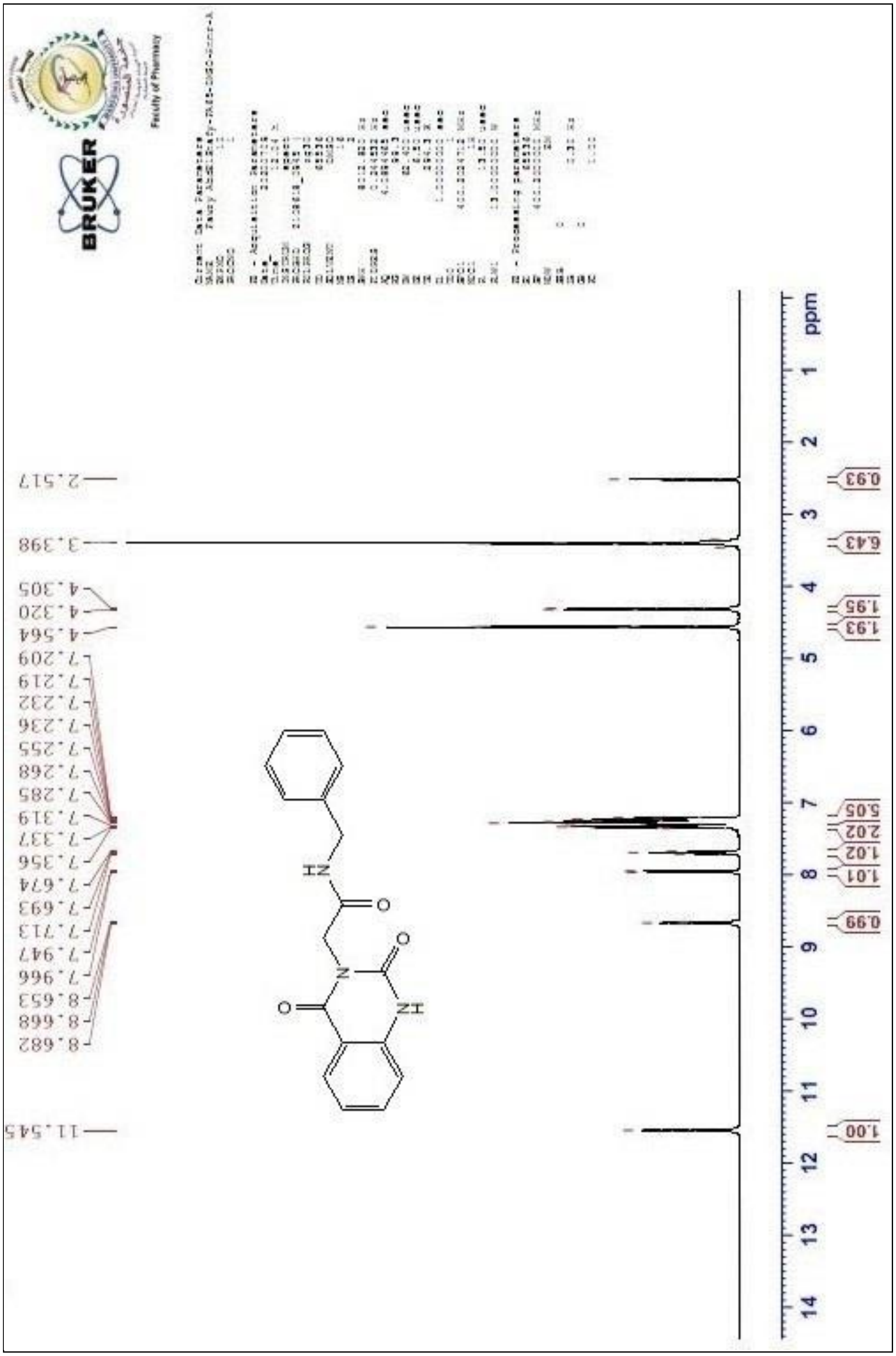

Figure 5: ^{1}H NMR spectrum of compound **2b**.

Figure 6: Mass spectrum of compound **2b**.

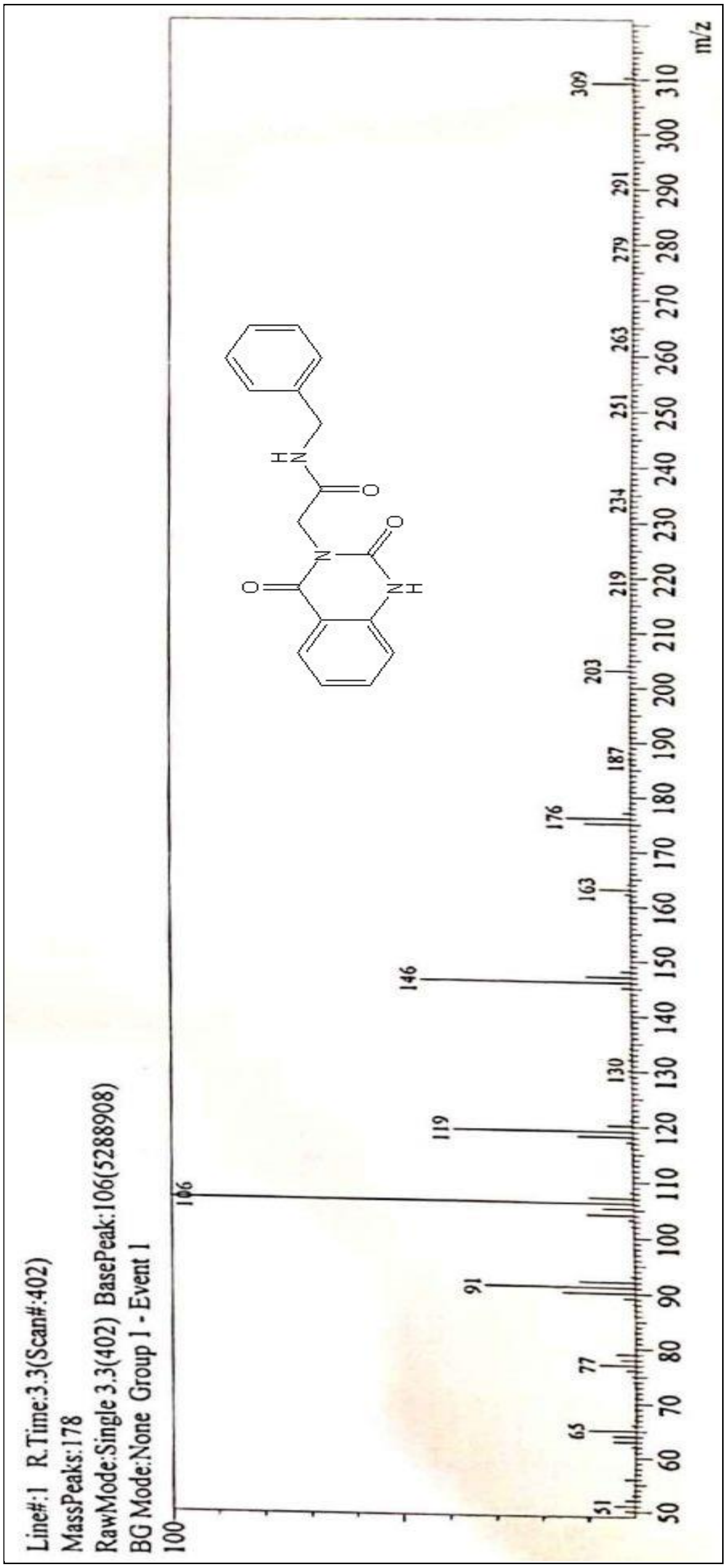

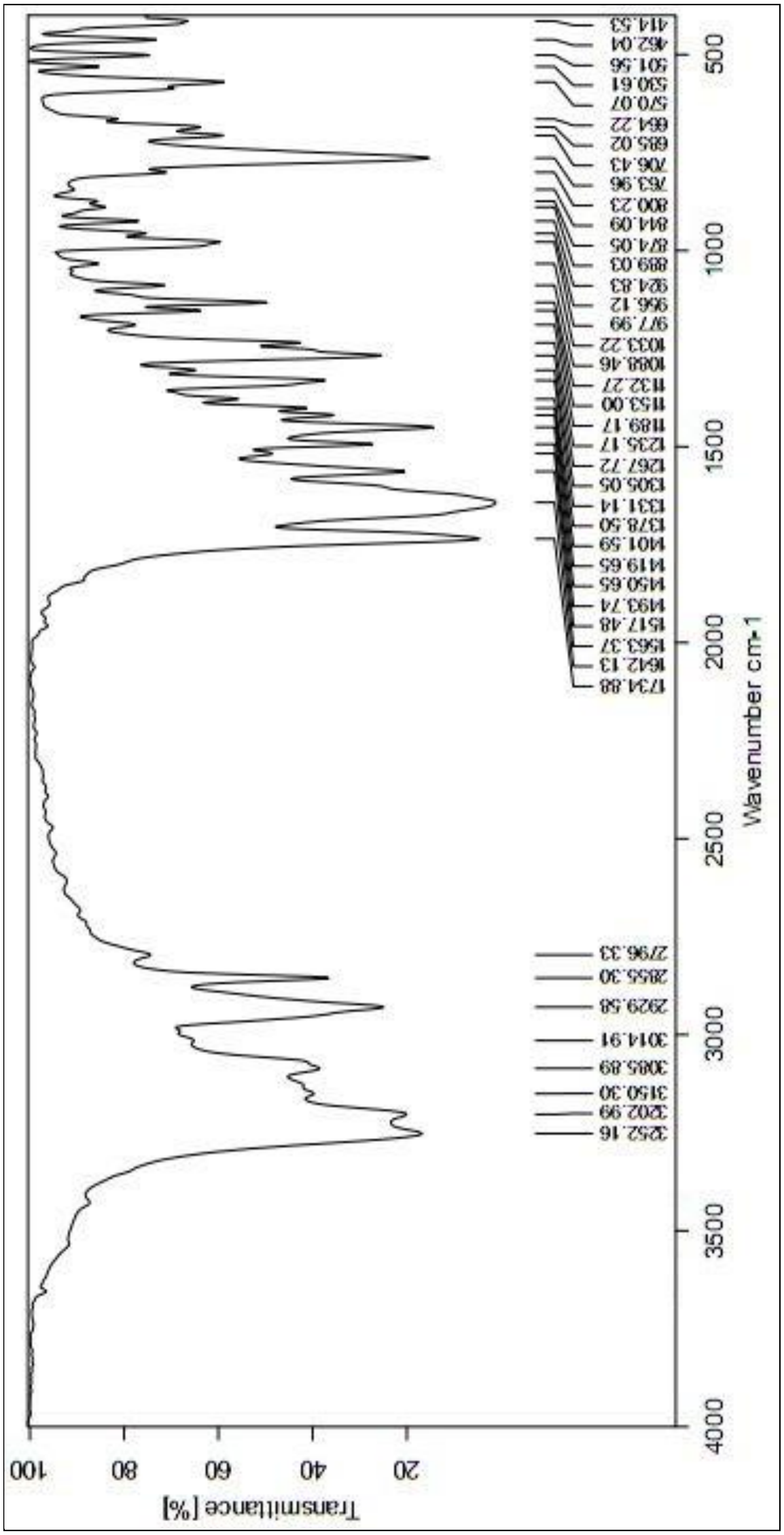

Figure 7: IR spectrum of compound **2c**.

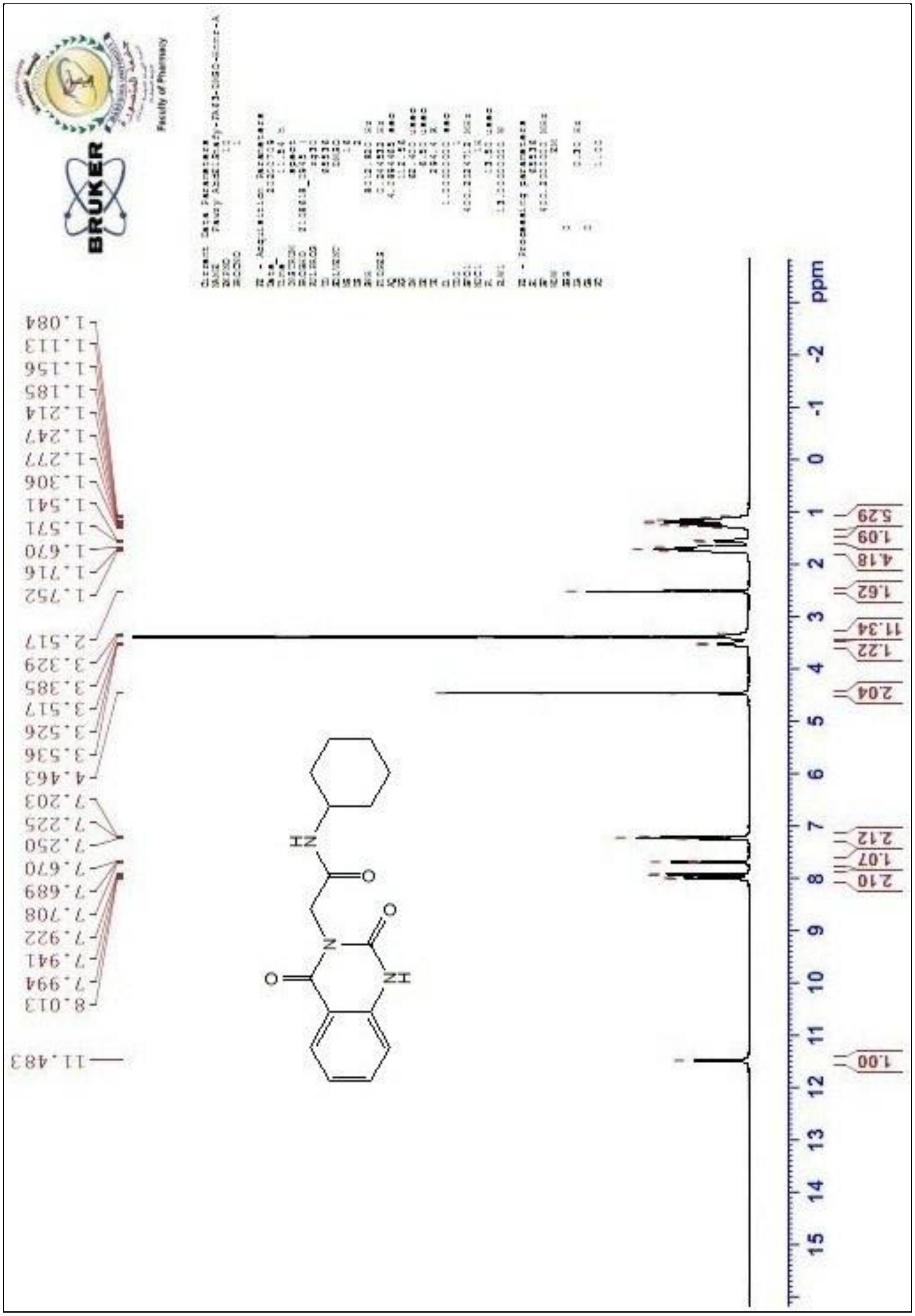

Figure 8: ^{1}H NMR spectrum of compound **2c**.

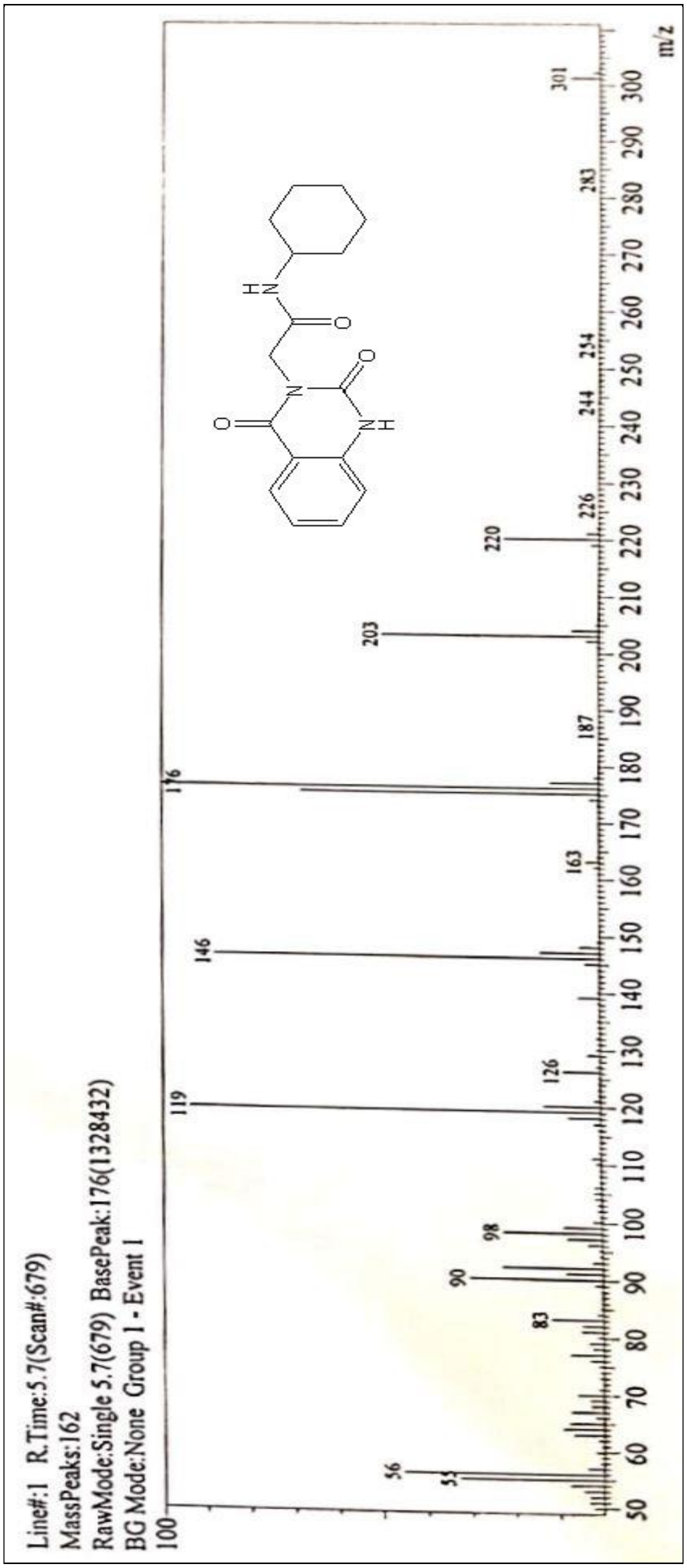

Figure 9: Mass spectrum of compound **2c**.

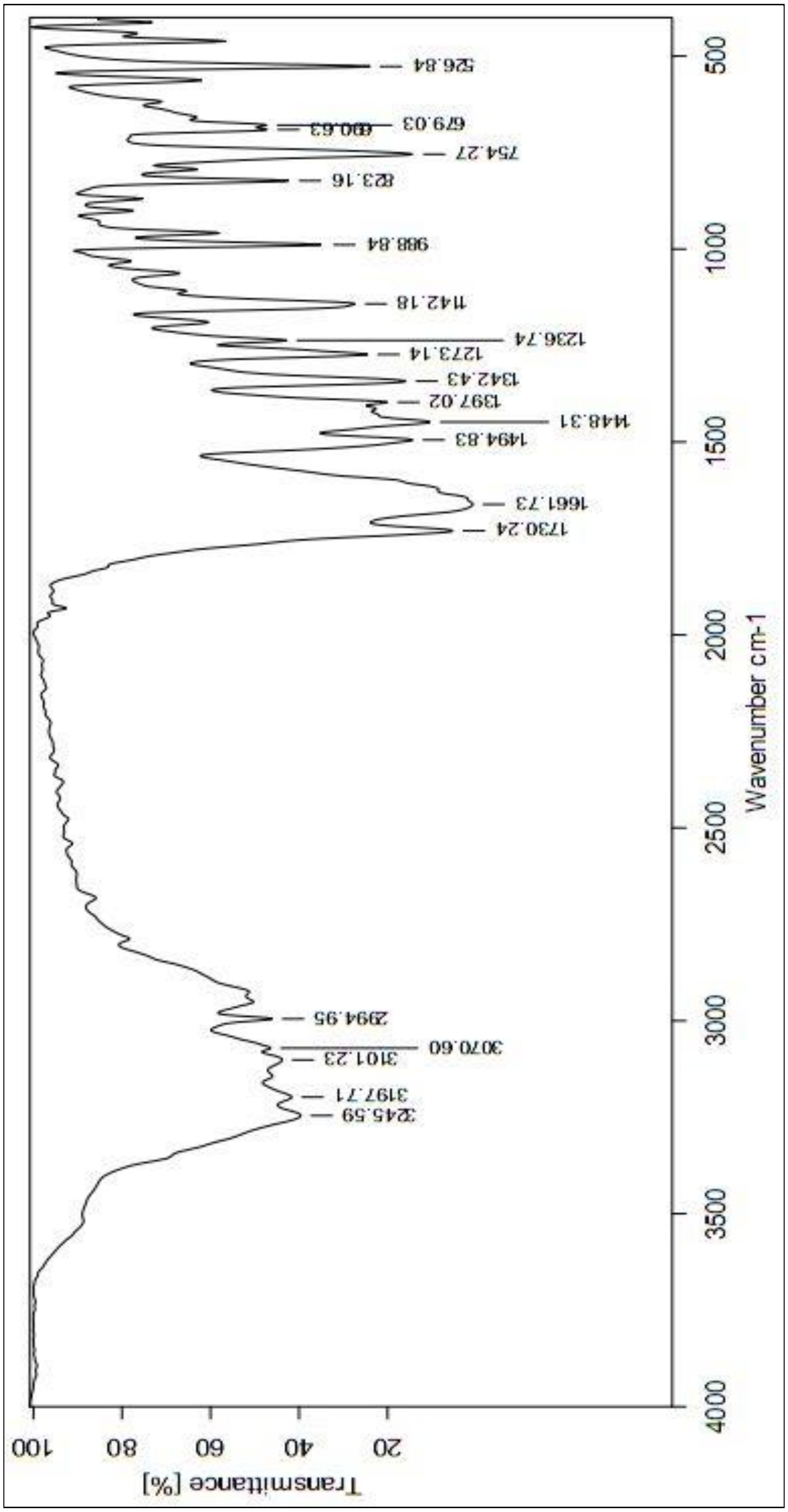

Figure 10: IR spectrum of compound **2d**.

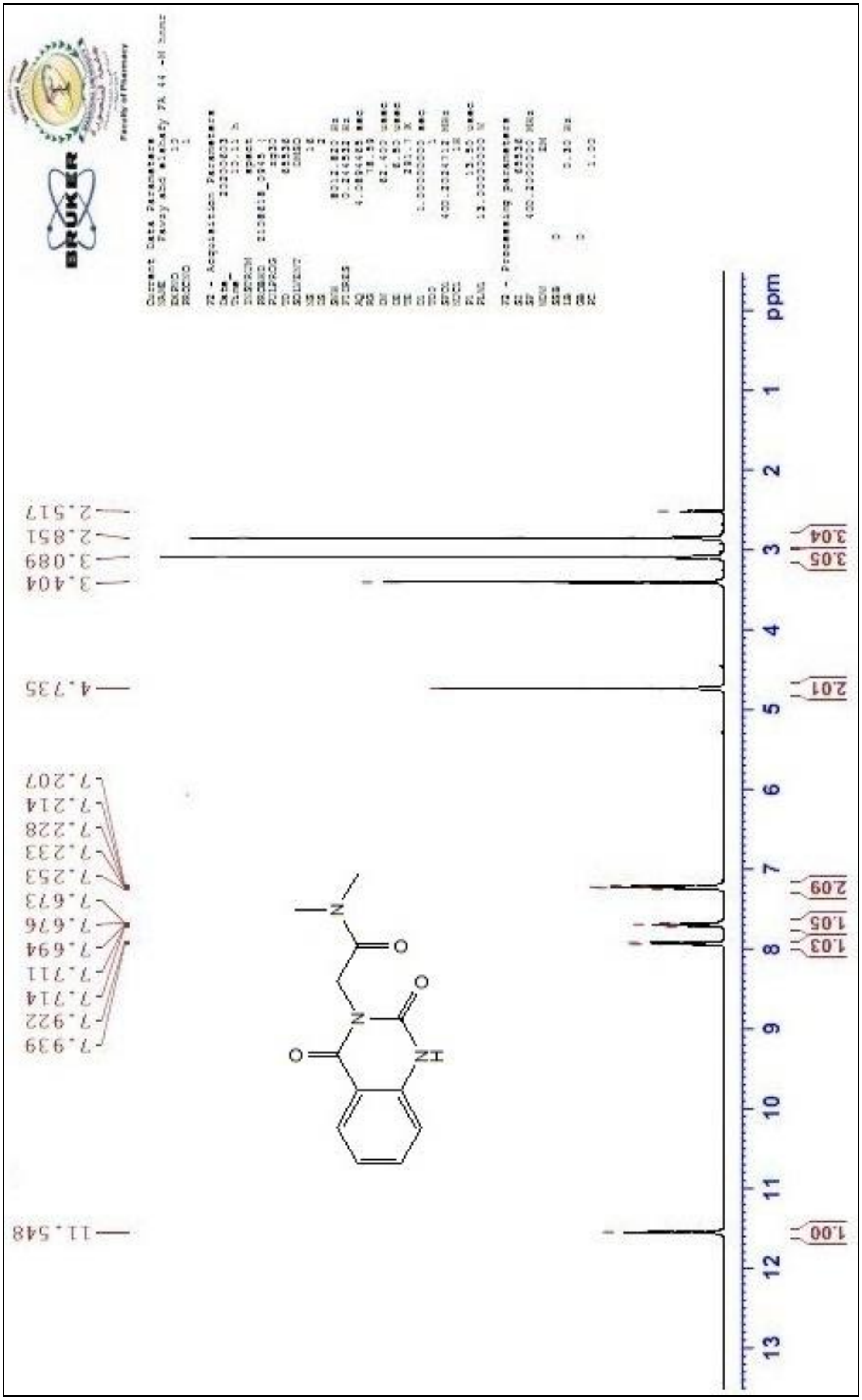

Figure 11: ^{1}H NMR spectrum of compound **2d**.

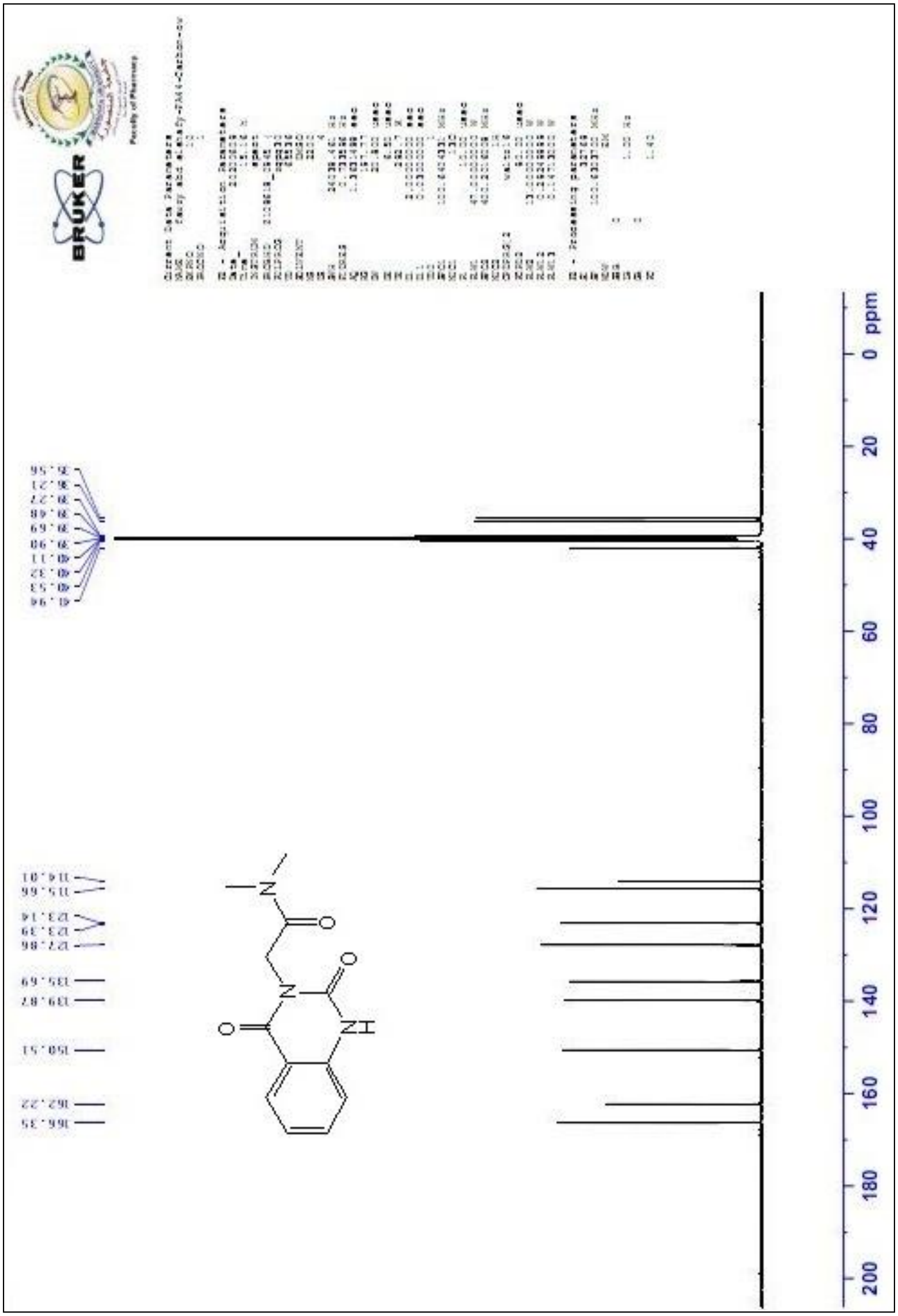

Figure 12: ^{13}C NMR spectrum of compound **2d**.

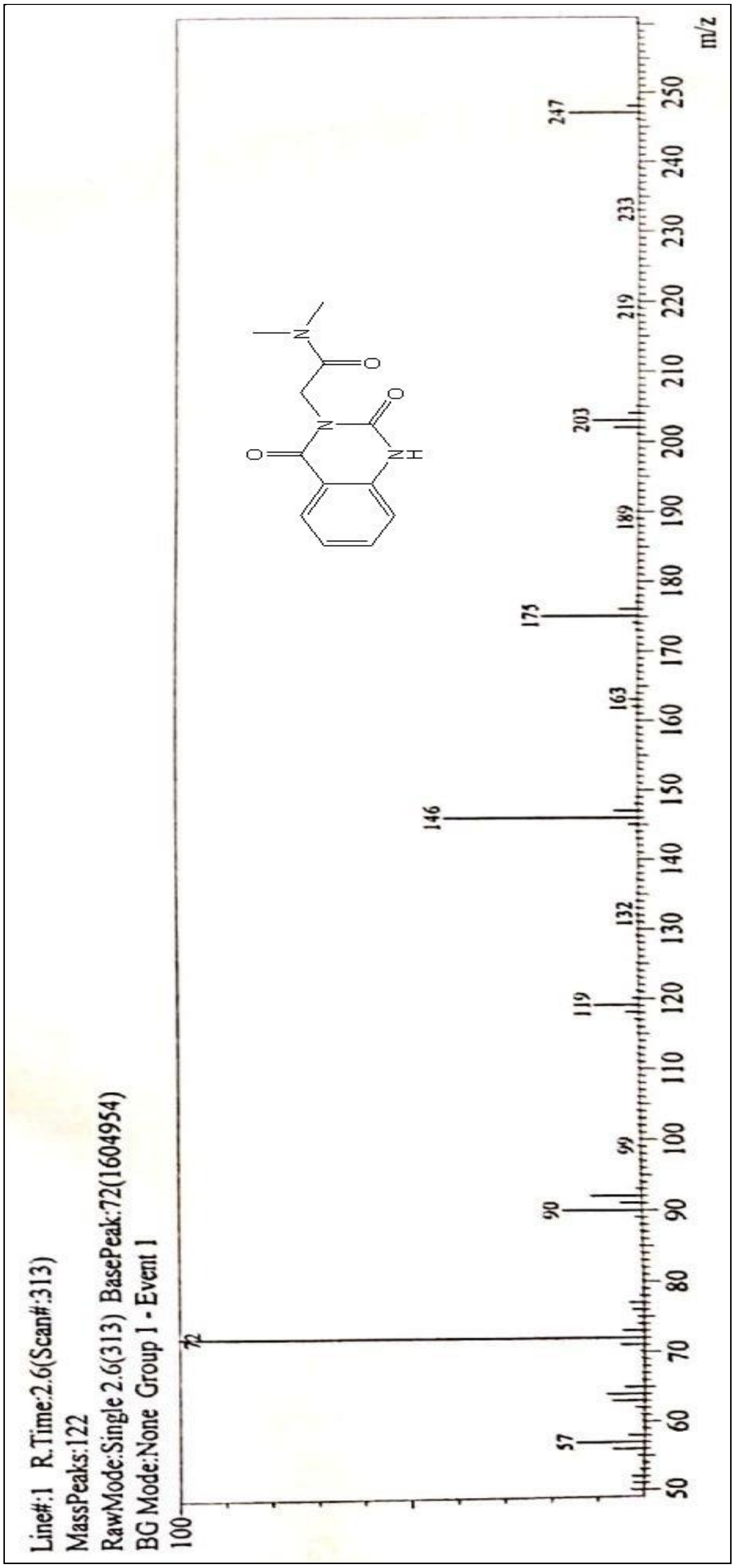

Figure 13: Mass spectrum of compound **2d**.

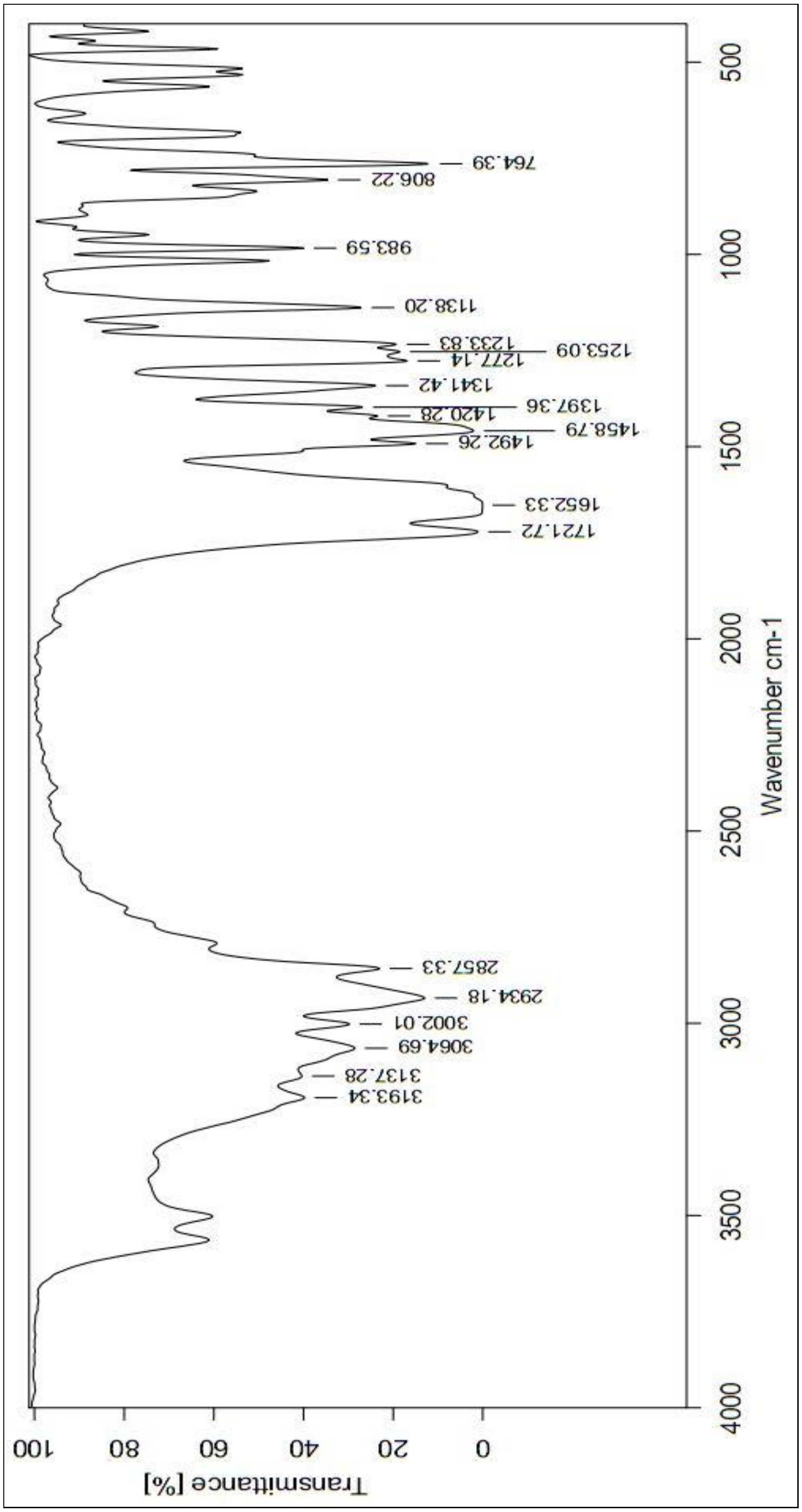

Figure 14: IR spectrum of compound **2e**.

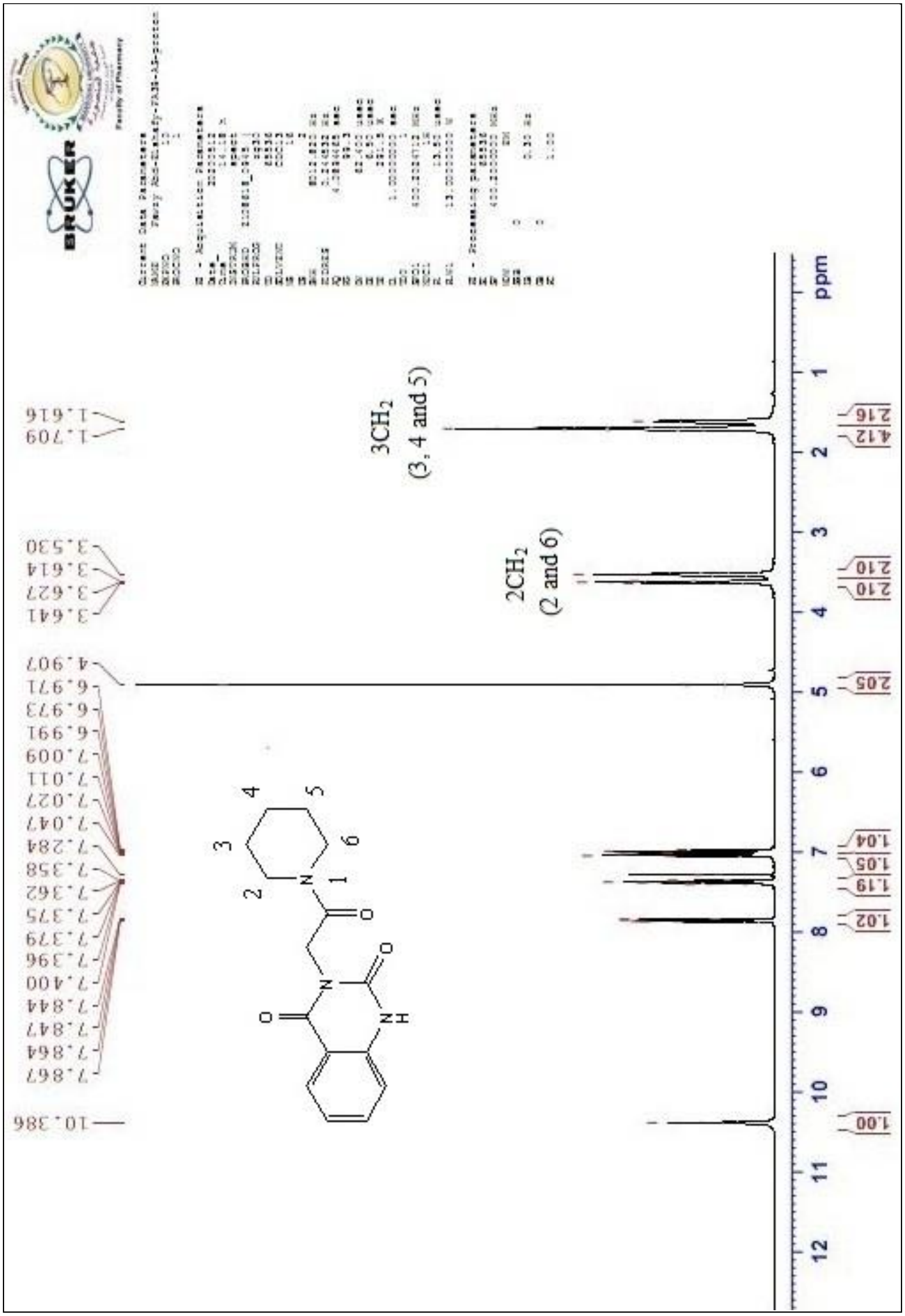

Figure 15: ^{1}H NMR spectrum of compound **2e**.

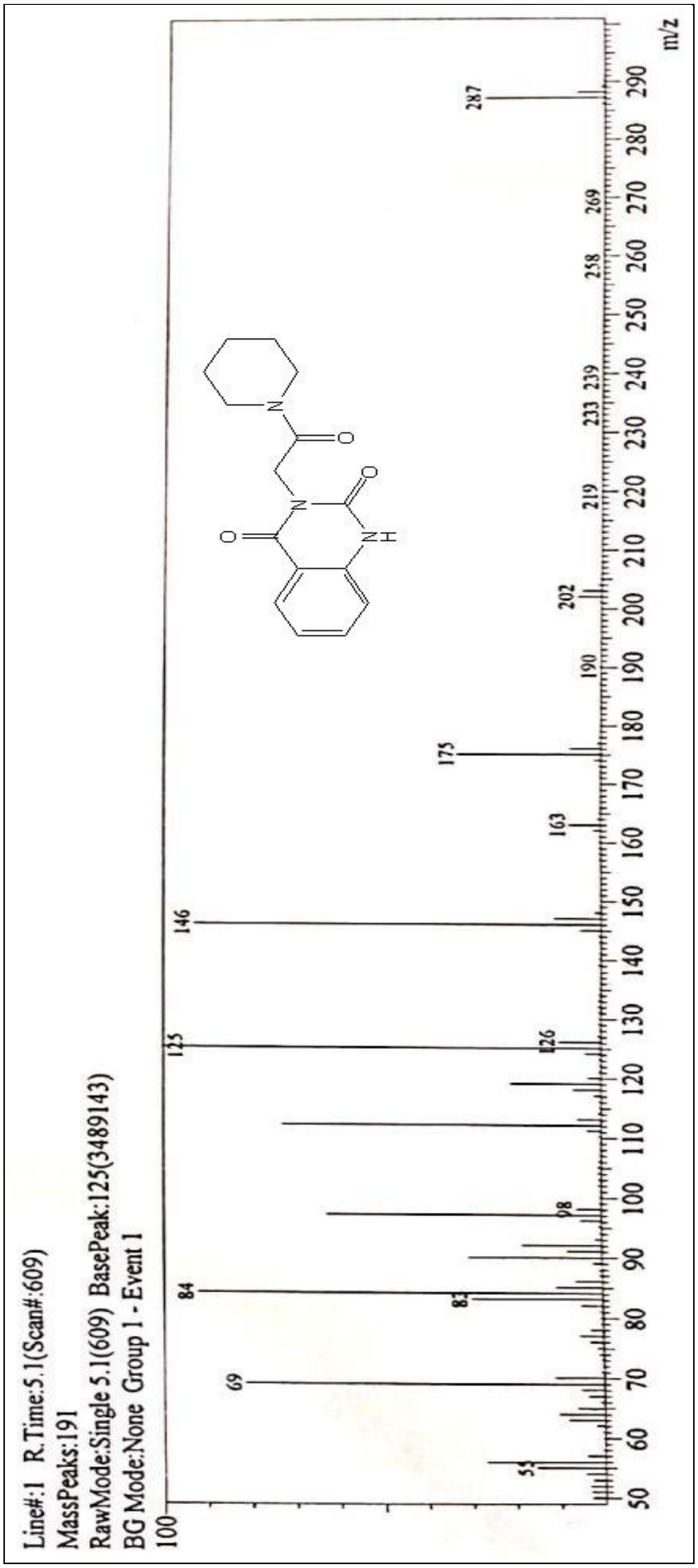

Figure 16: Mass spectrum of compound **2e**.

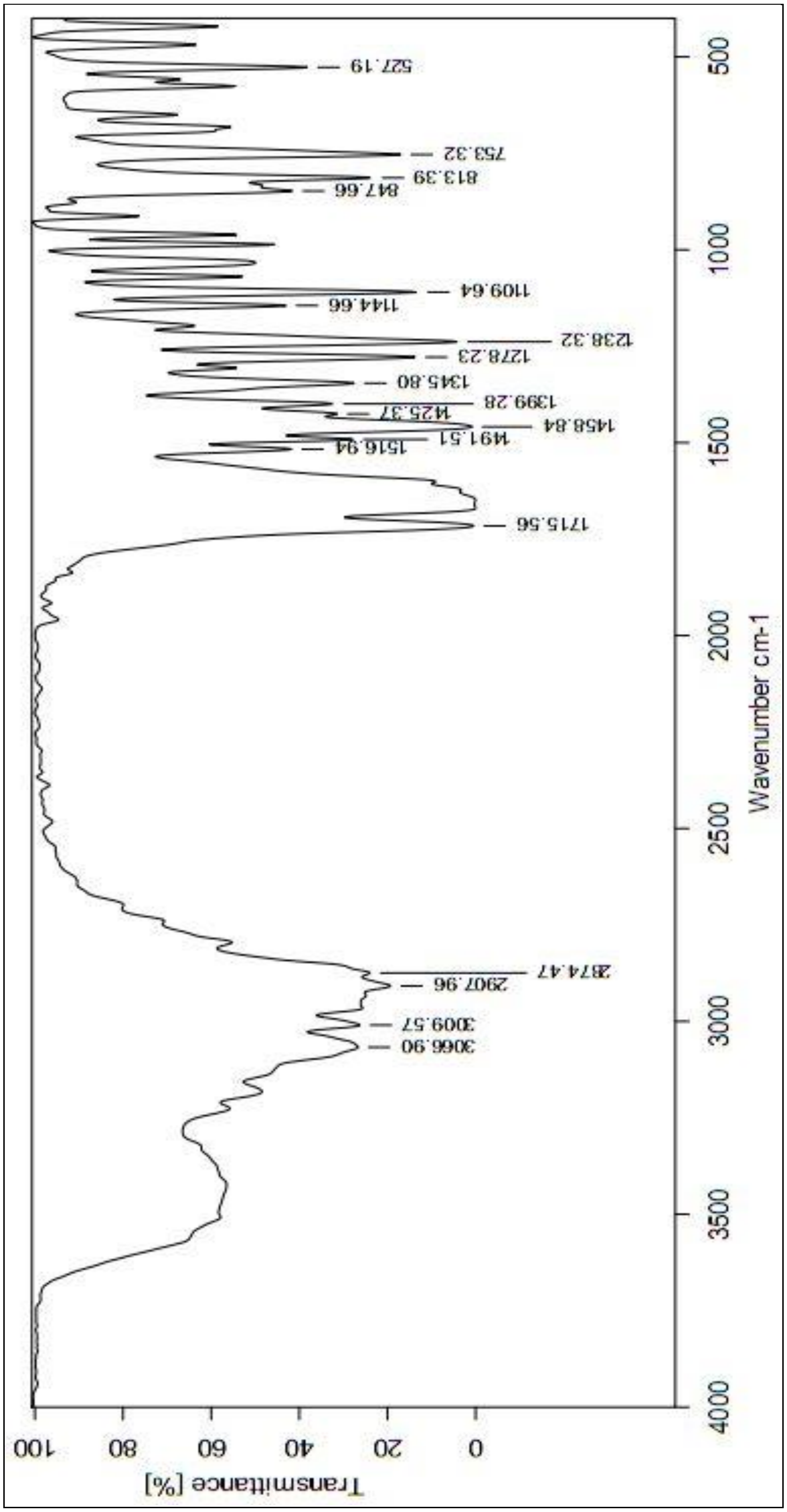

Figure 17: IR spectrum of compound **2f**.

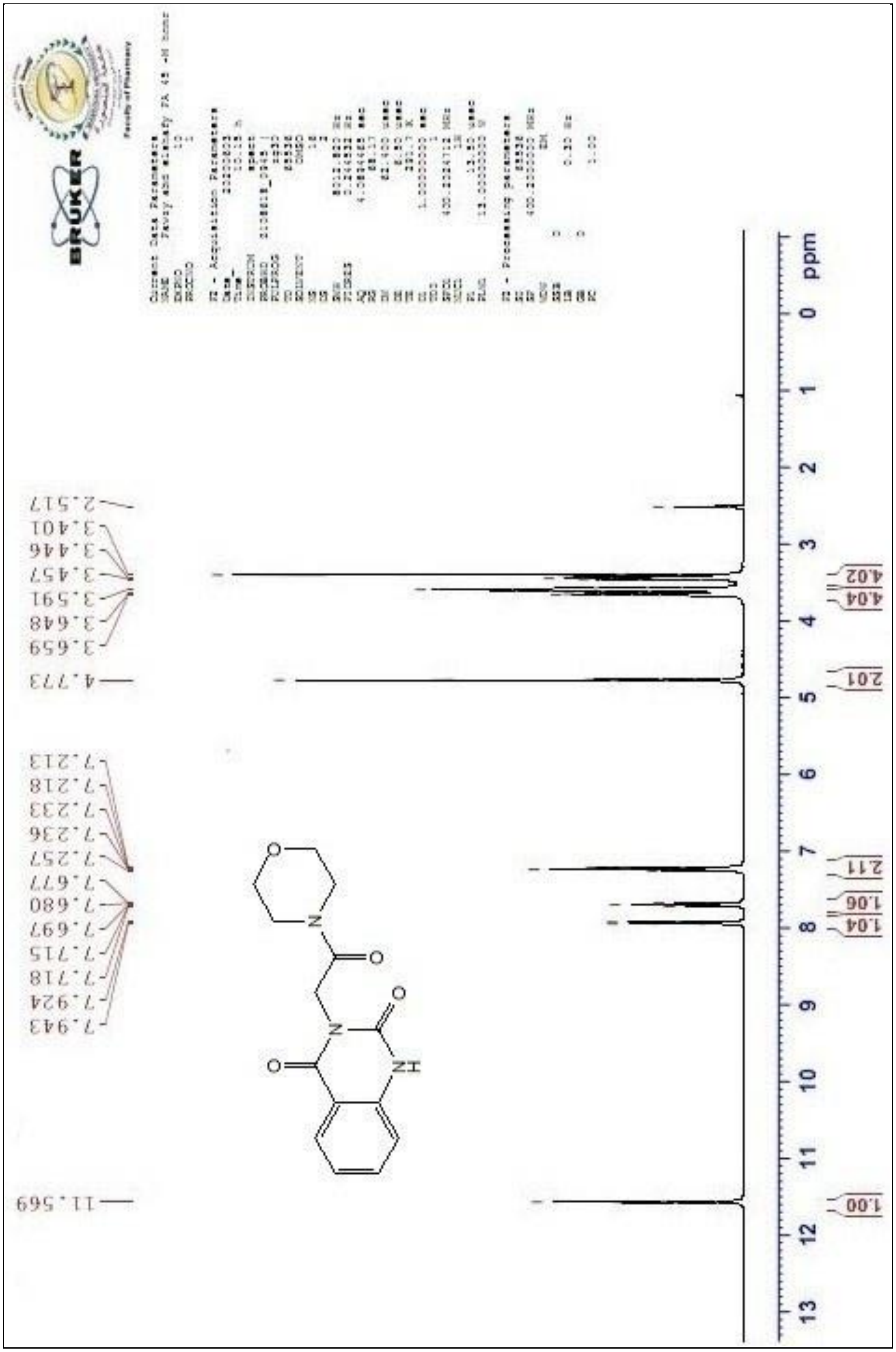

Figure 18: ^{1}H NMR spectrum of compound **2f**.

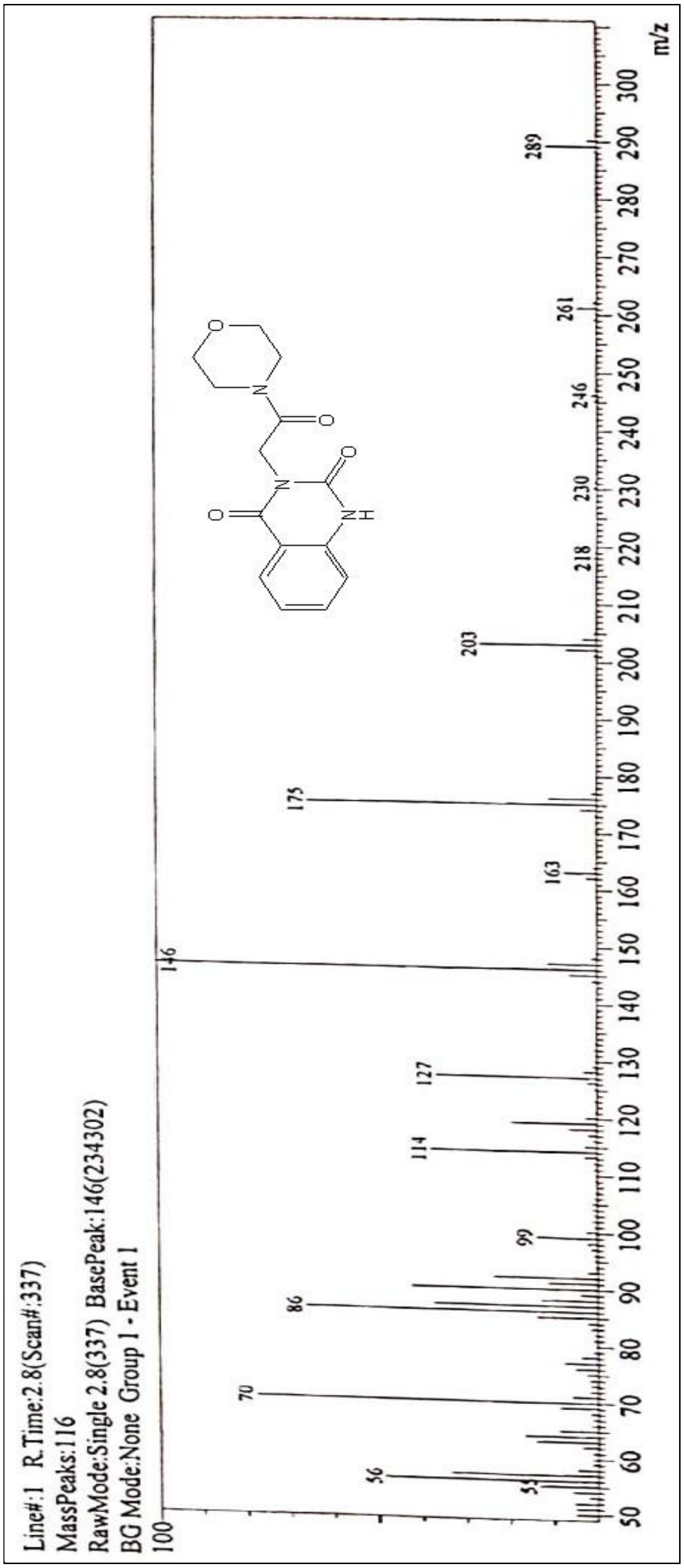

Figure 19: Mass spectrum of compound **2f**.

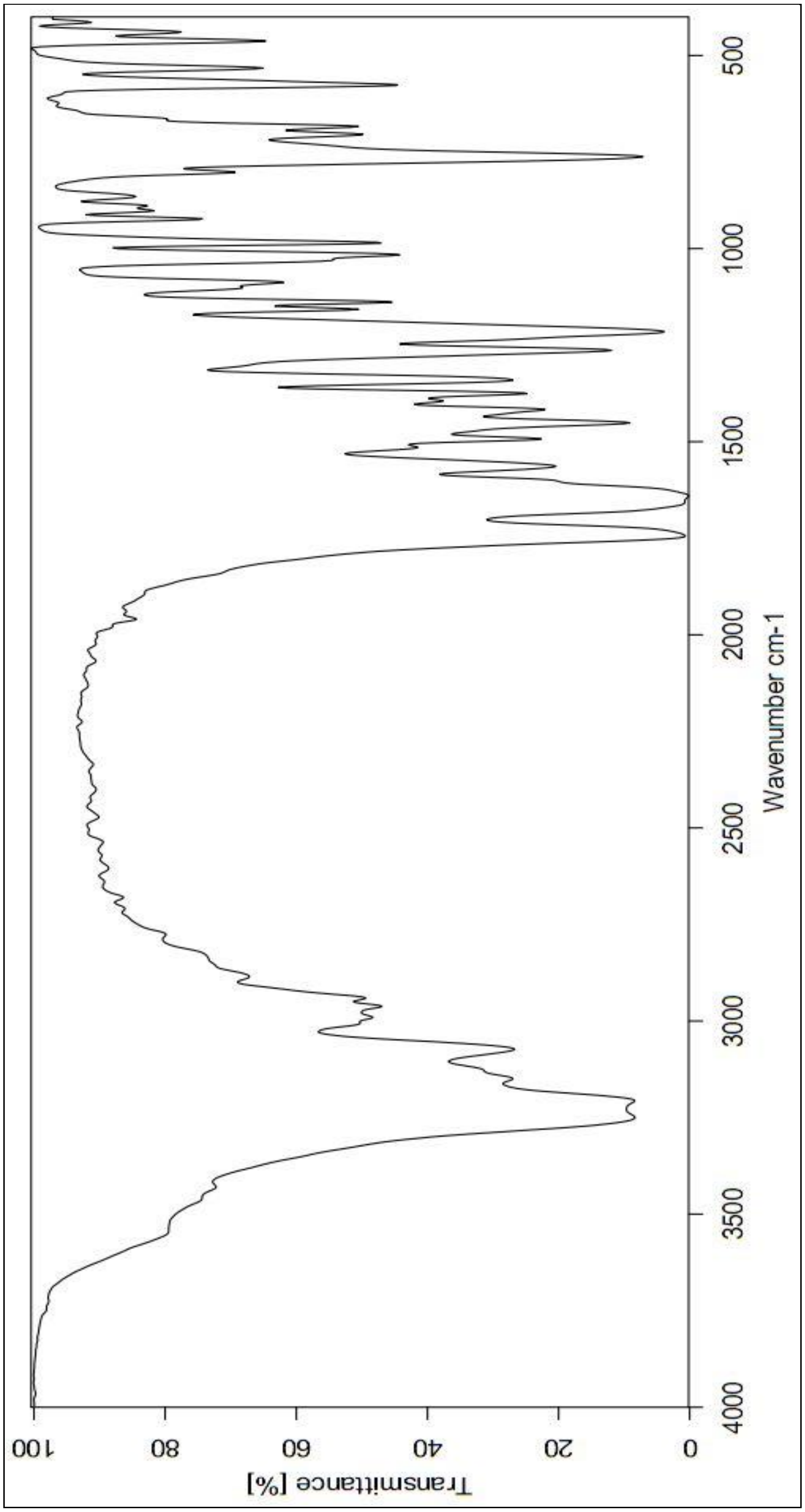

Figure 20: IR spectrum of compound **6g**.

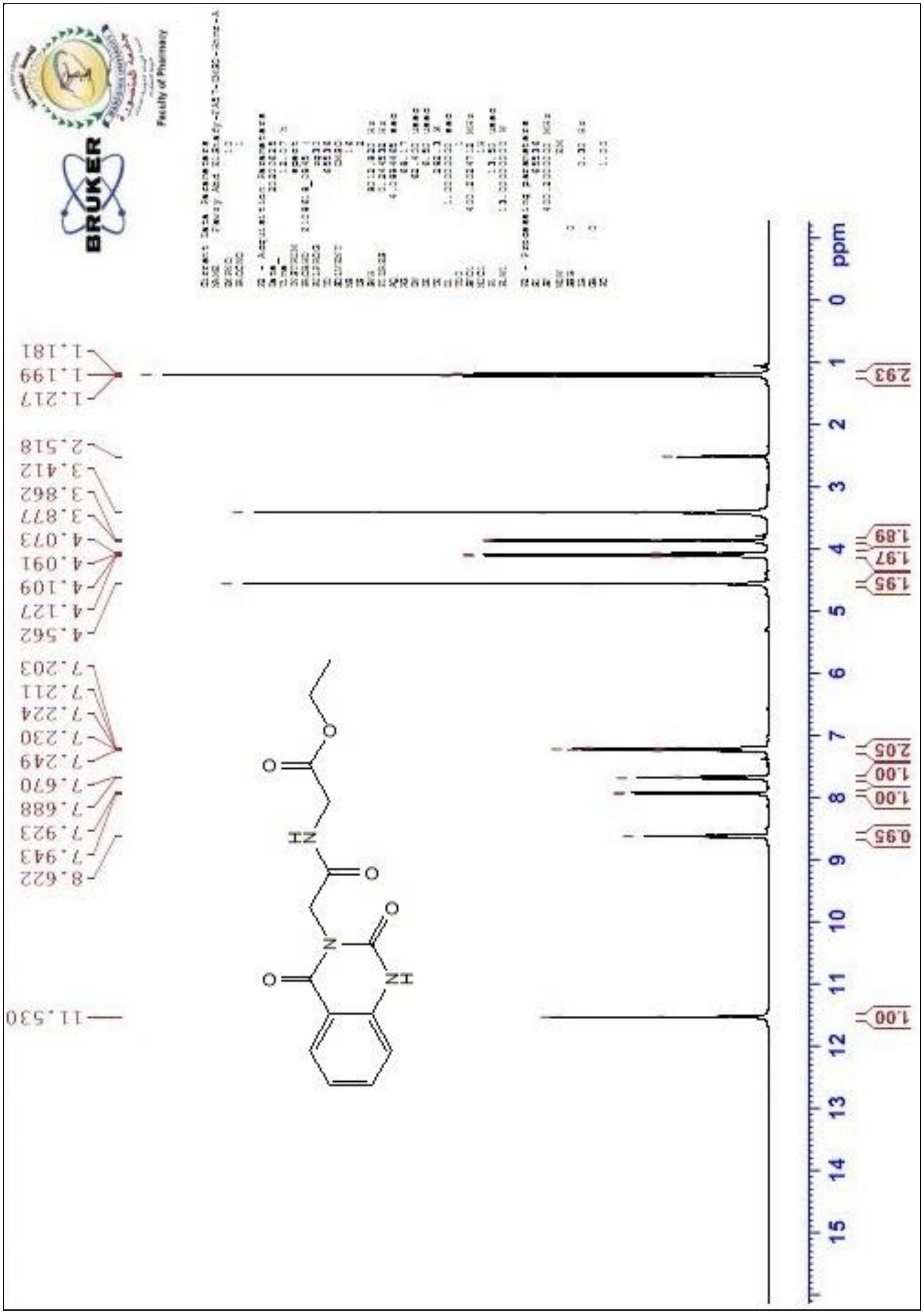

Figure 21: ^{1}H NMR spectrum of compound **6g**.

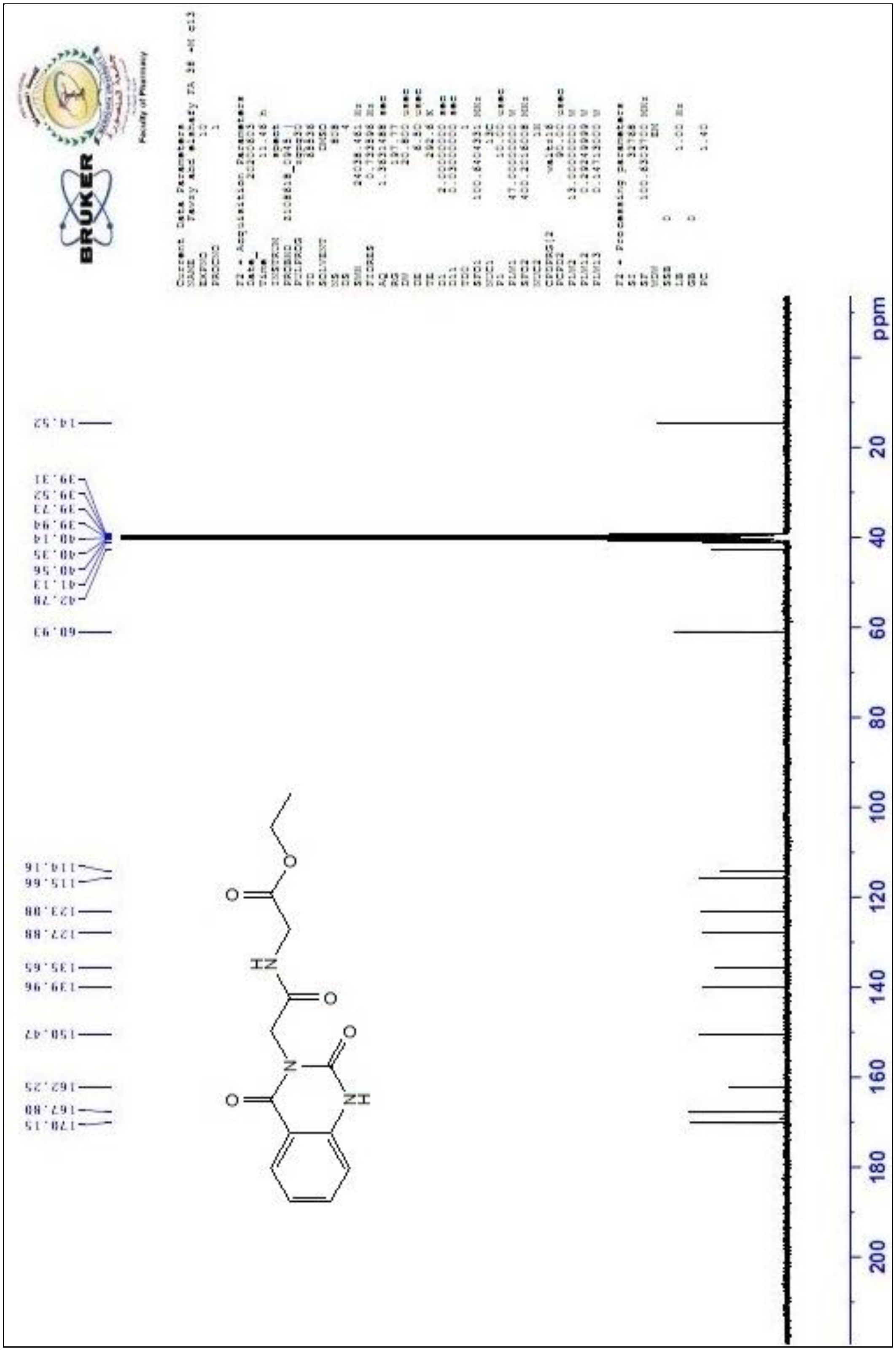

Figure 22: ^{13}C NMR spectrum of compound **6g**.

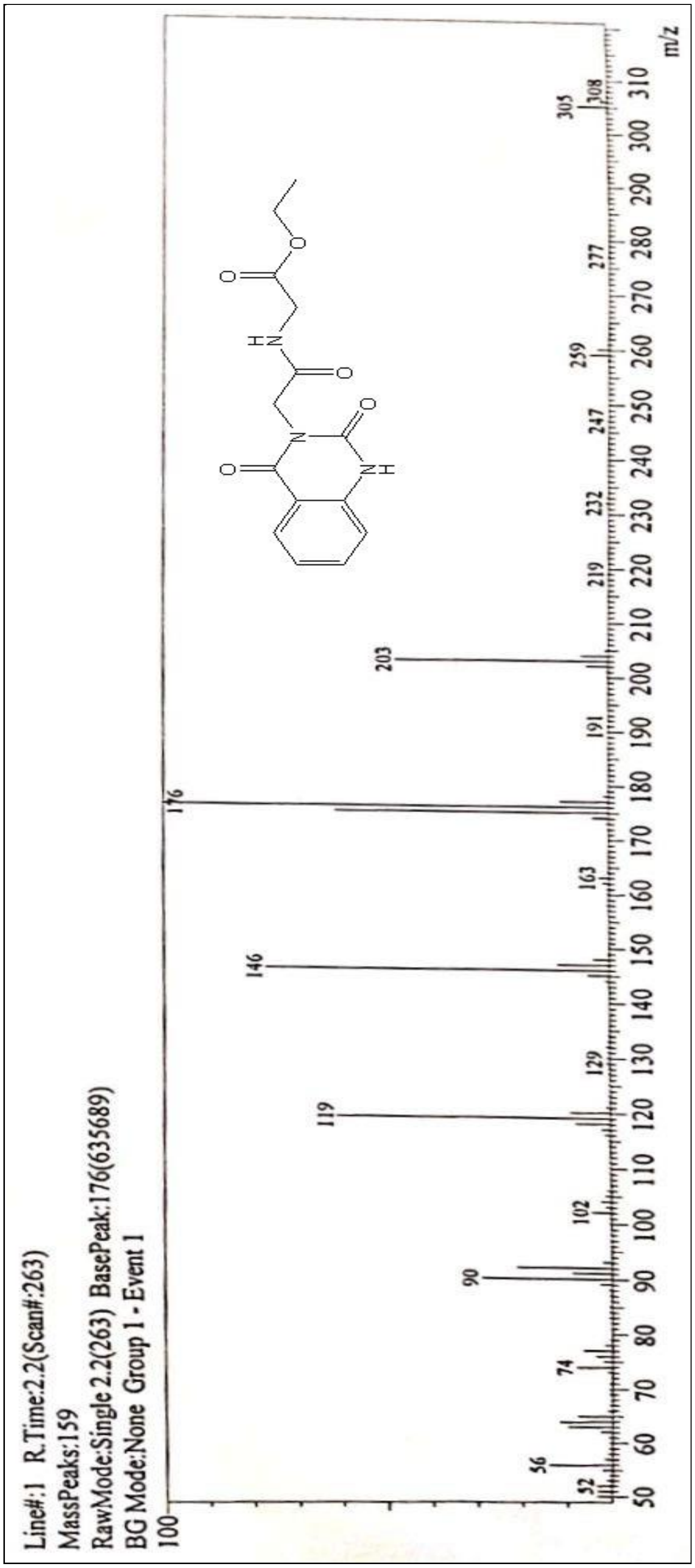

Figure 23: Mass spectrum of compound **6g**.

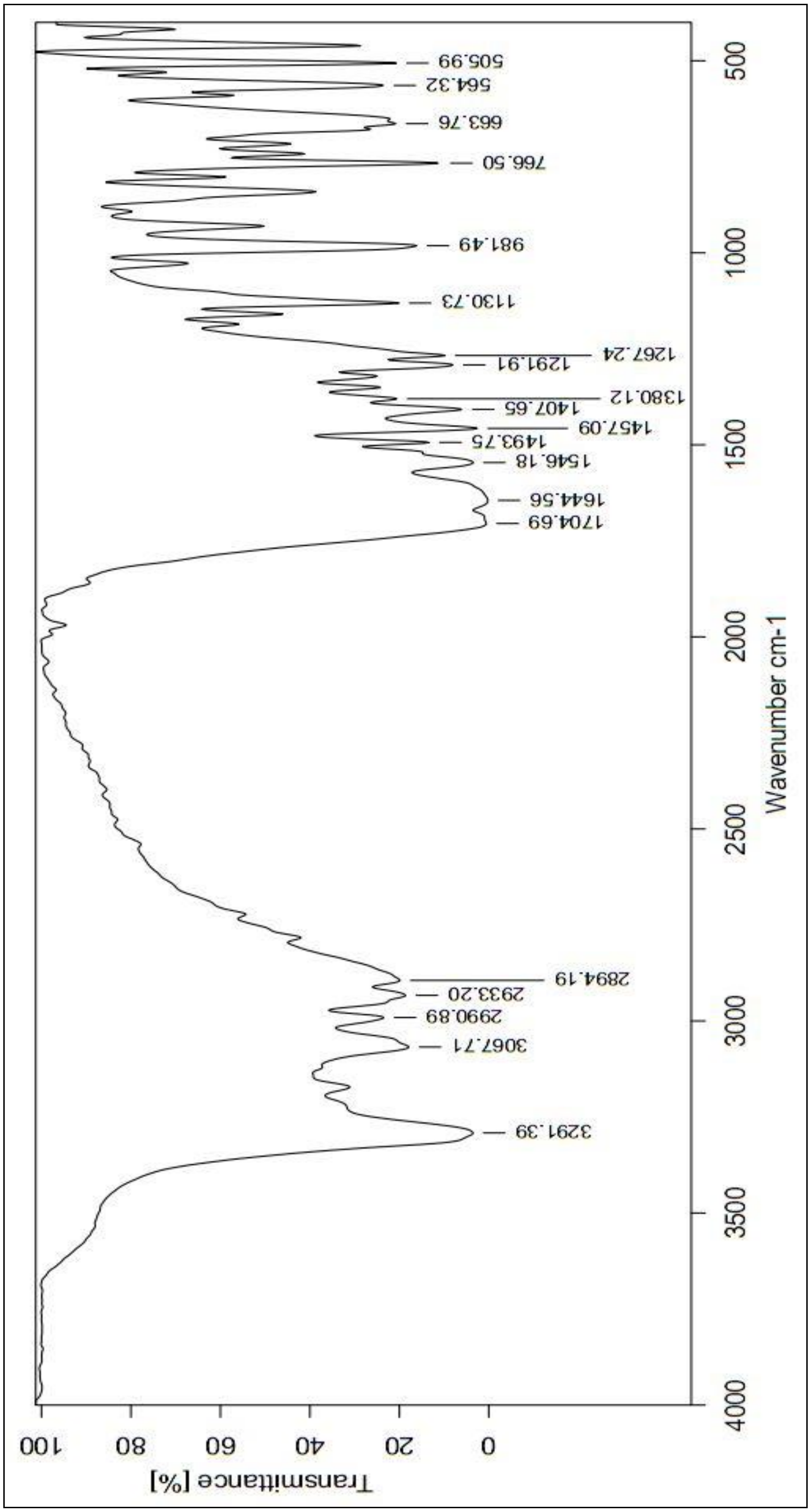

Figure 24: IR spectrum of compound **3**.

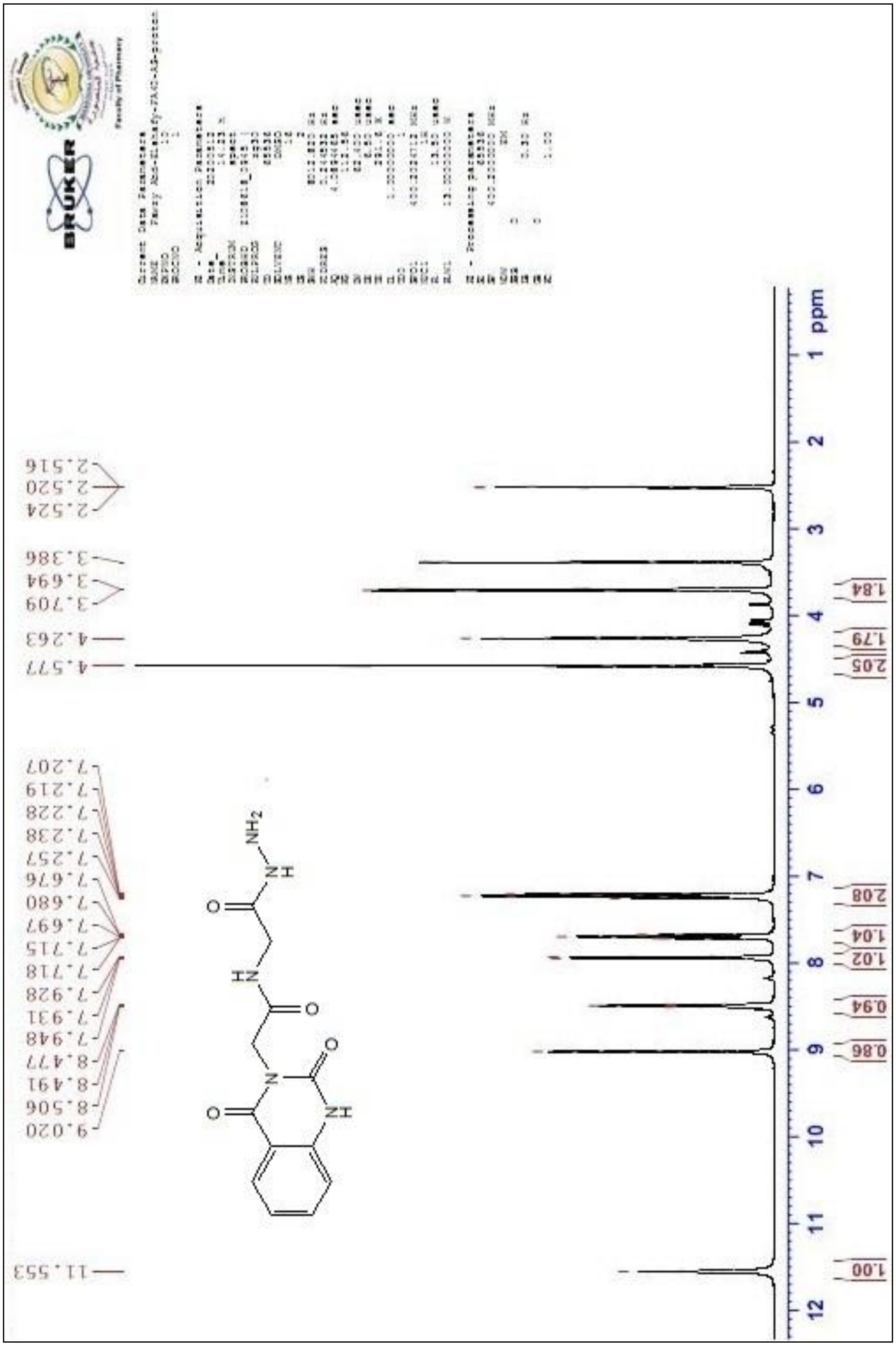

Figure 25: ^{1}H NMR spectrum of compound **3**.

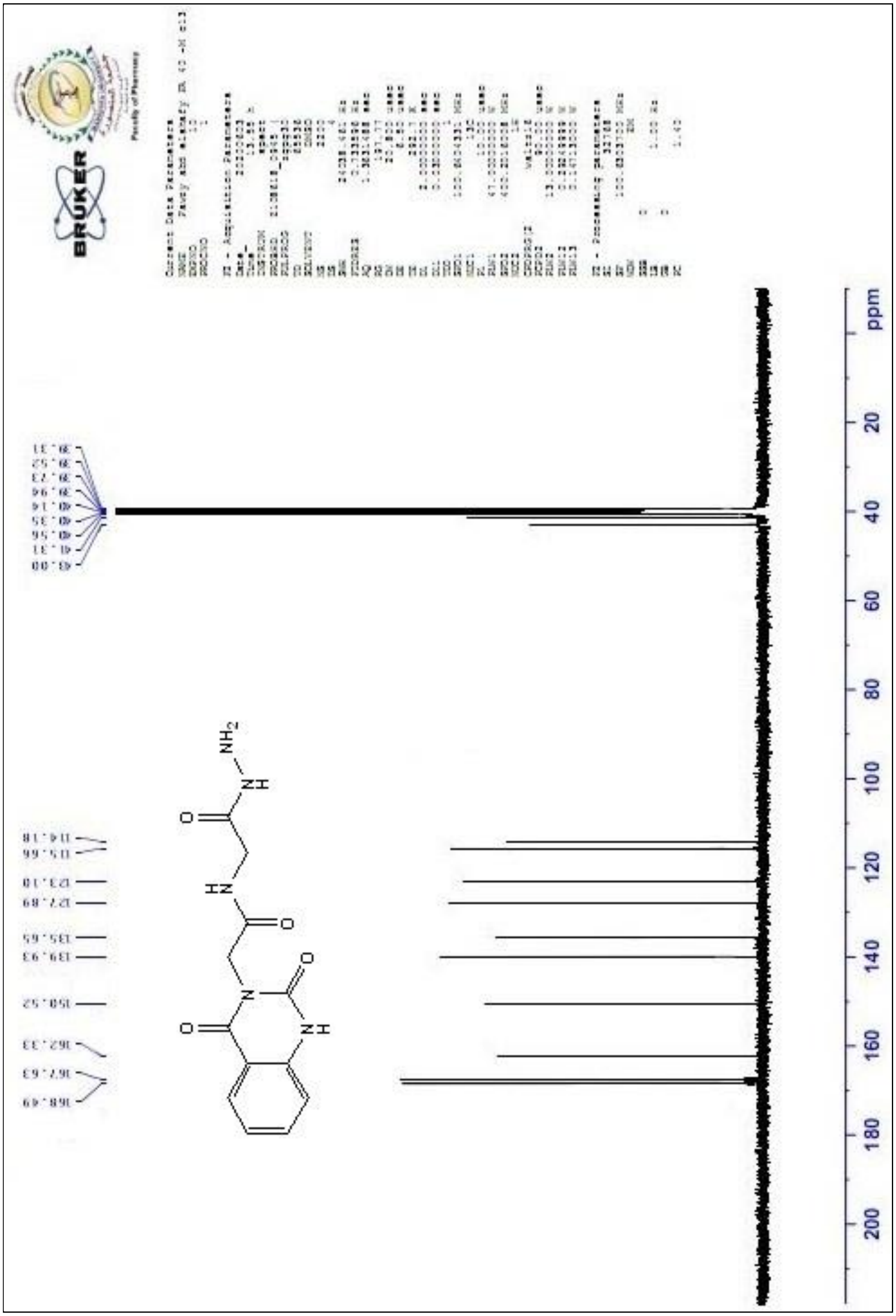

Figure 26: ^{13}C NMR spectrum of compound **3**.

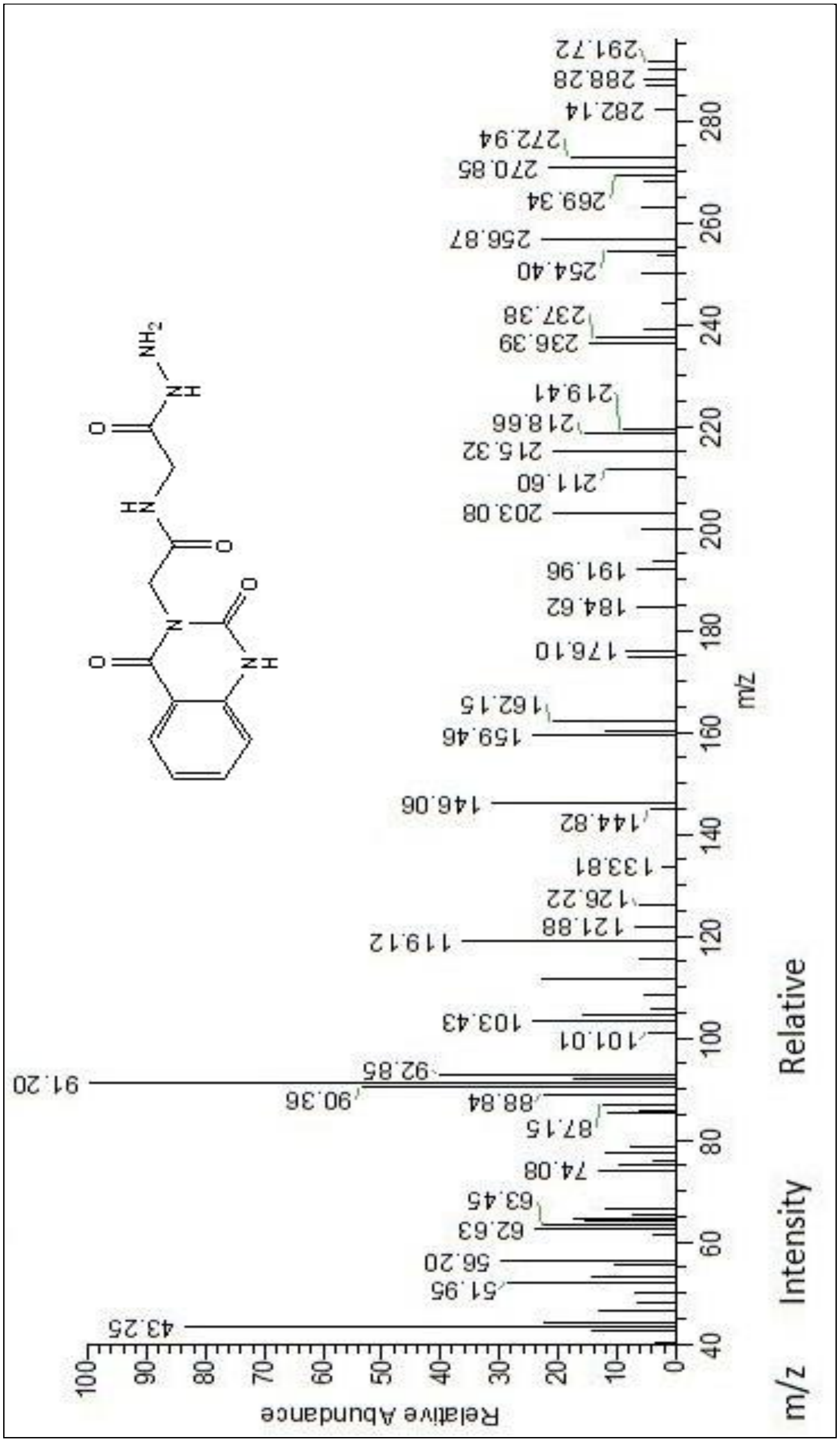

Figure 27: Mass spectrum of compound **3**.

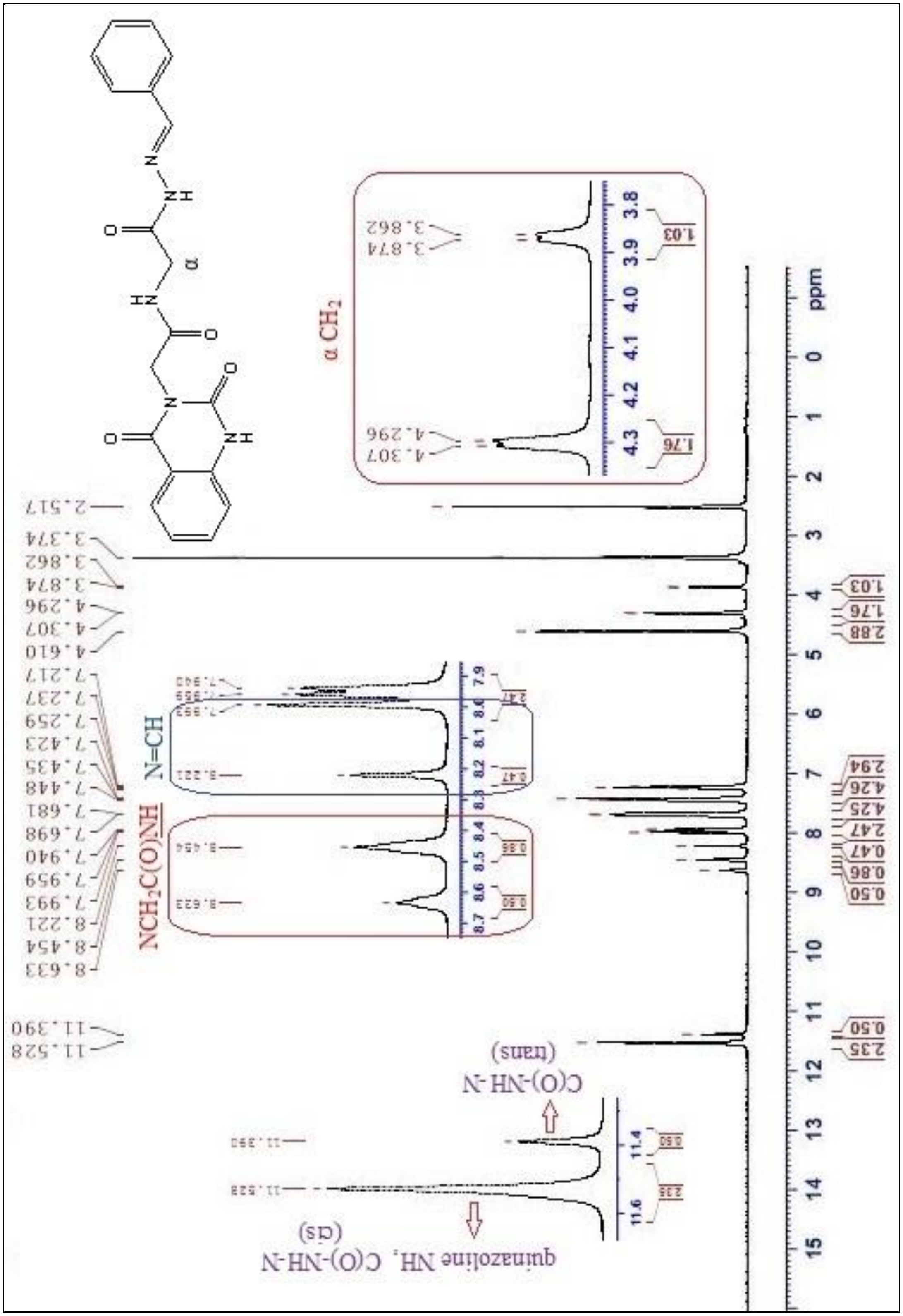

Figure 28: ^{1}H NMR spectrum of compound **4a**.

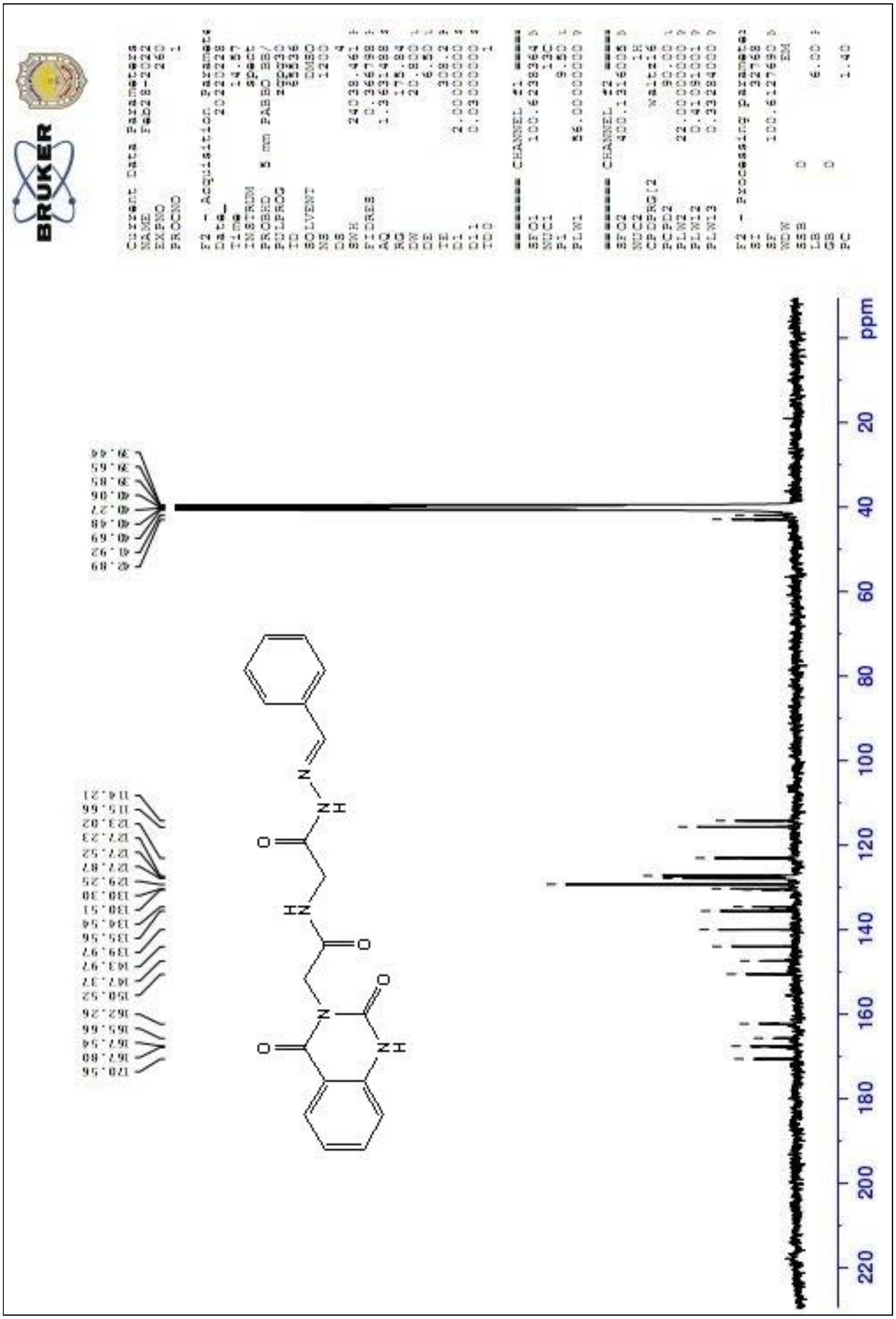

Figure 29: ^{13}C NMR spectrum of compound **4a**.

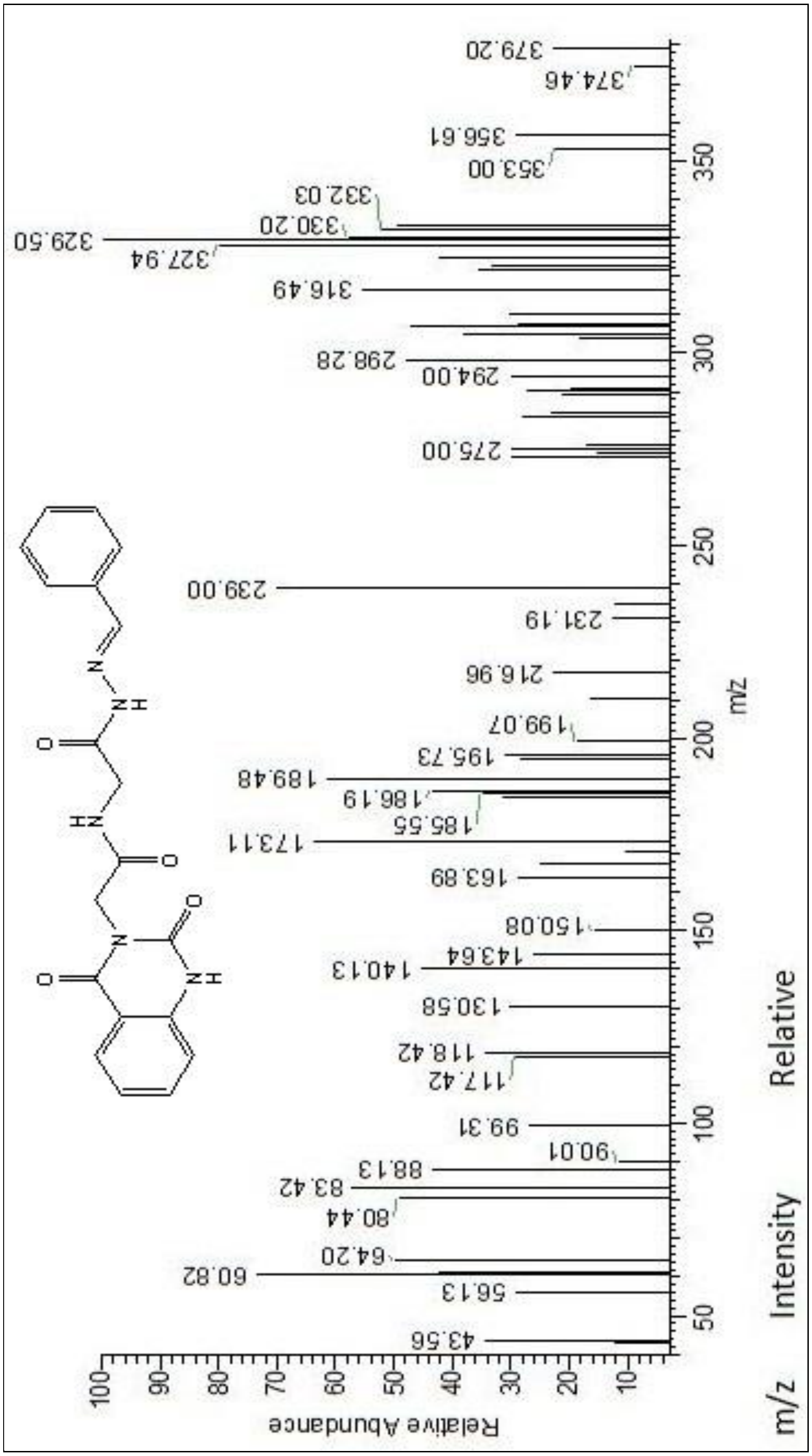

Figure 30: Mass spectrum of compound **4a**.

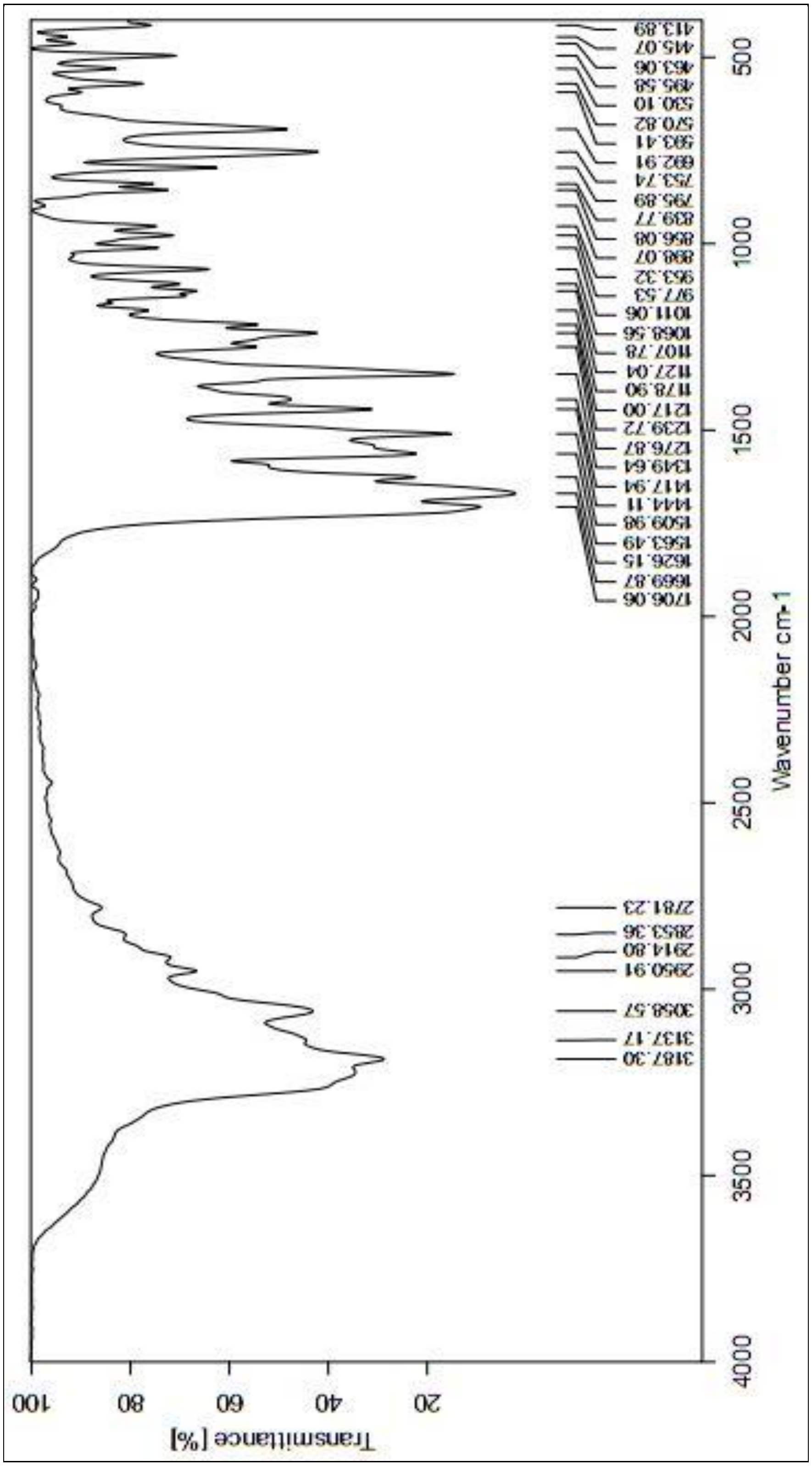

Figure 31: IR spectrum of compound **4b**.

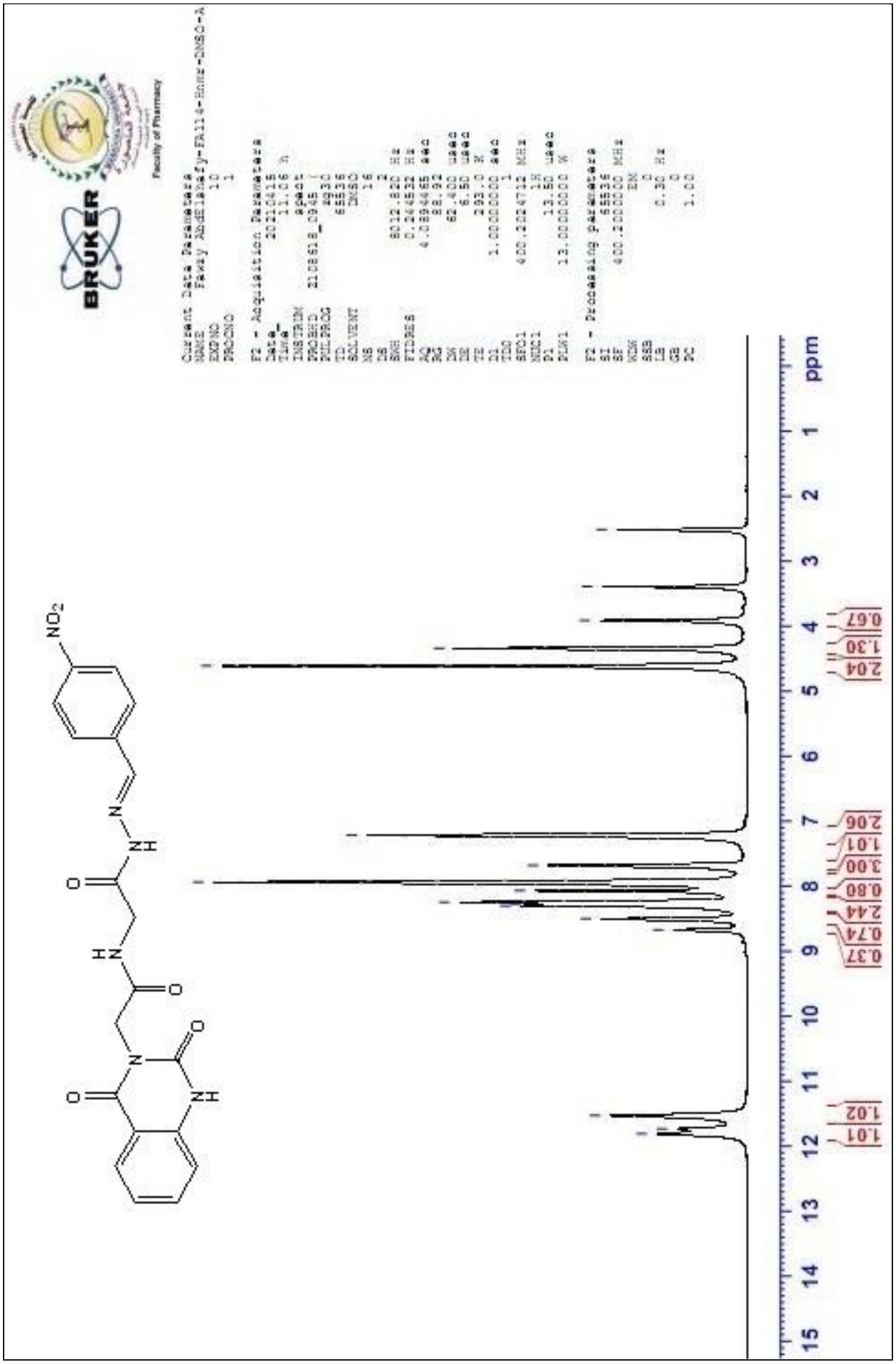

Figure 32: ^{1}H NMR spectrum of compound **4b**.

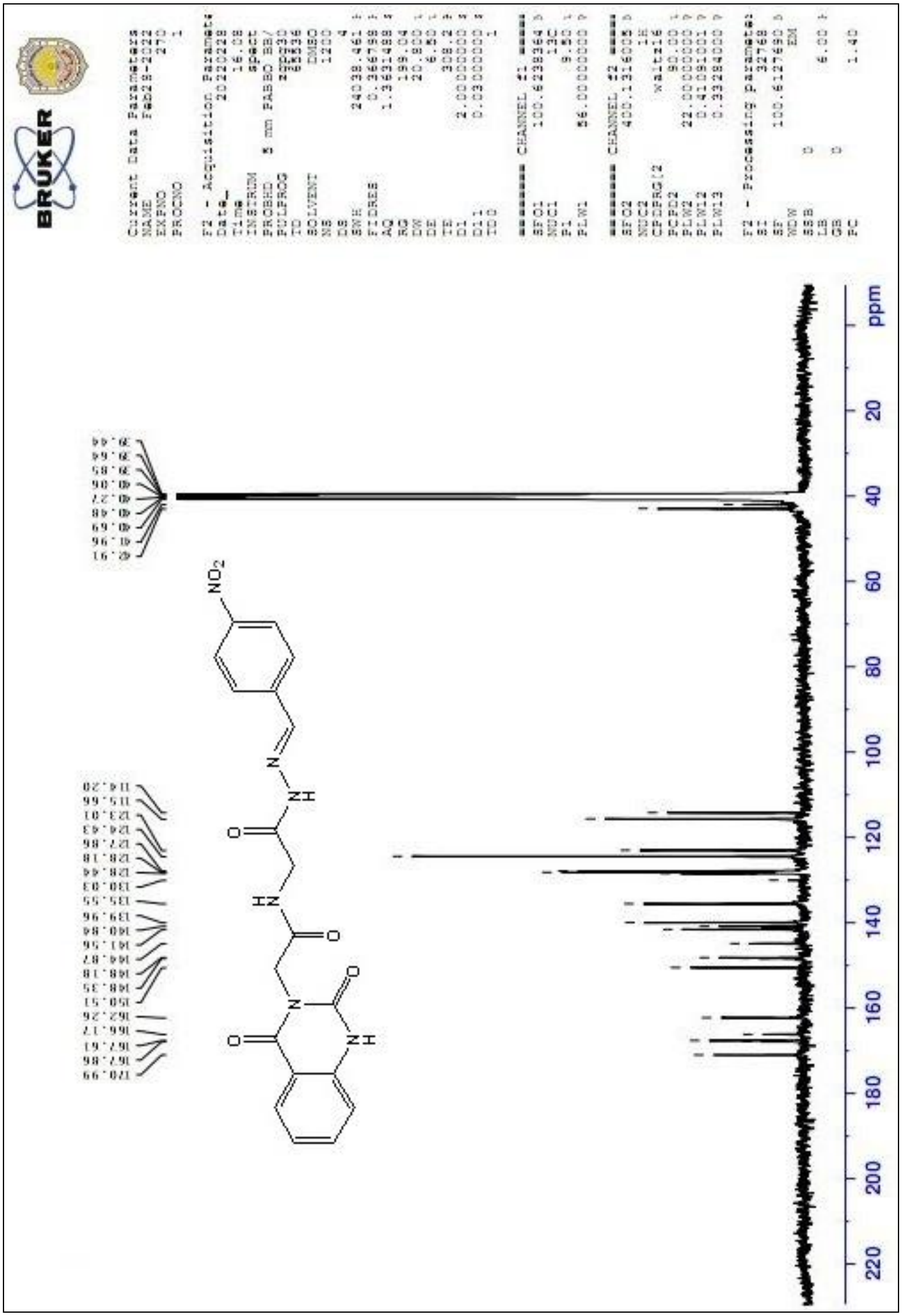

Figure 33: ^{13}C NMR spectrum of compound **4b**.

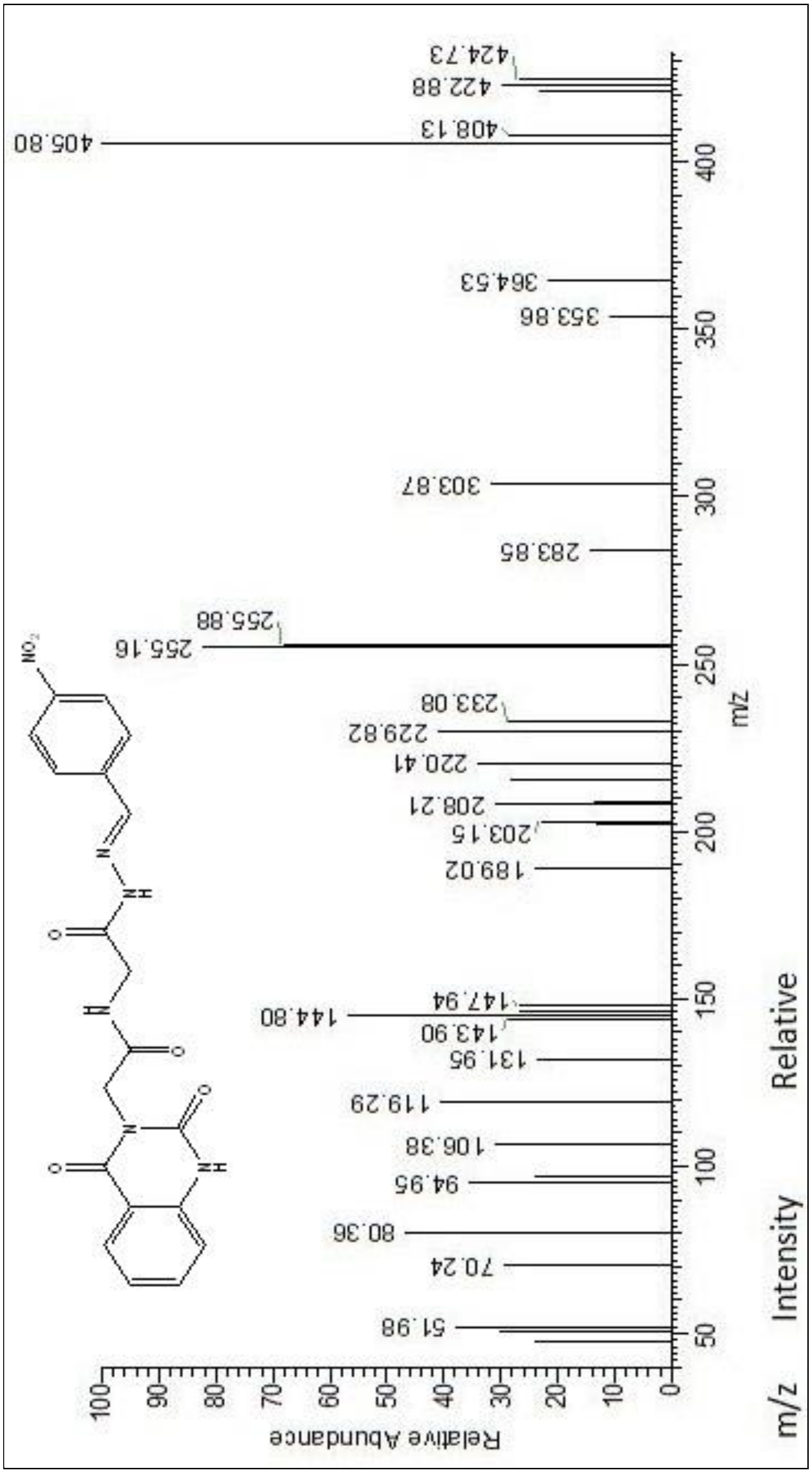

Figure 34: Mass spectrum of compound **4b**.

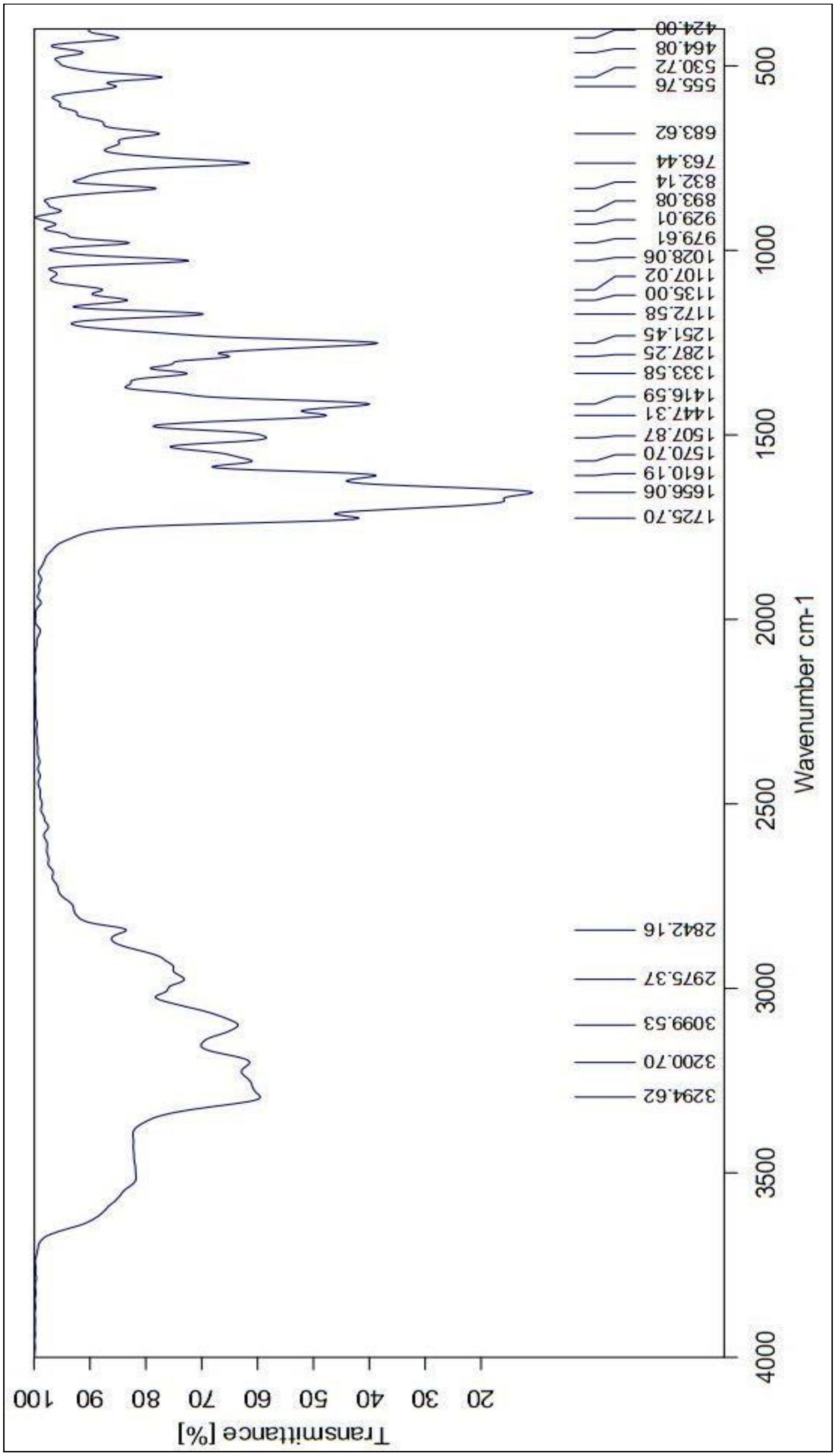

Figure 35: IR spectrum of compound **4c**.

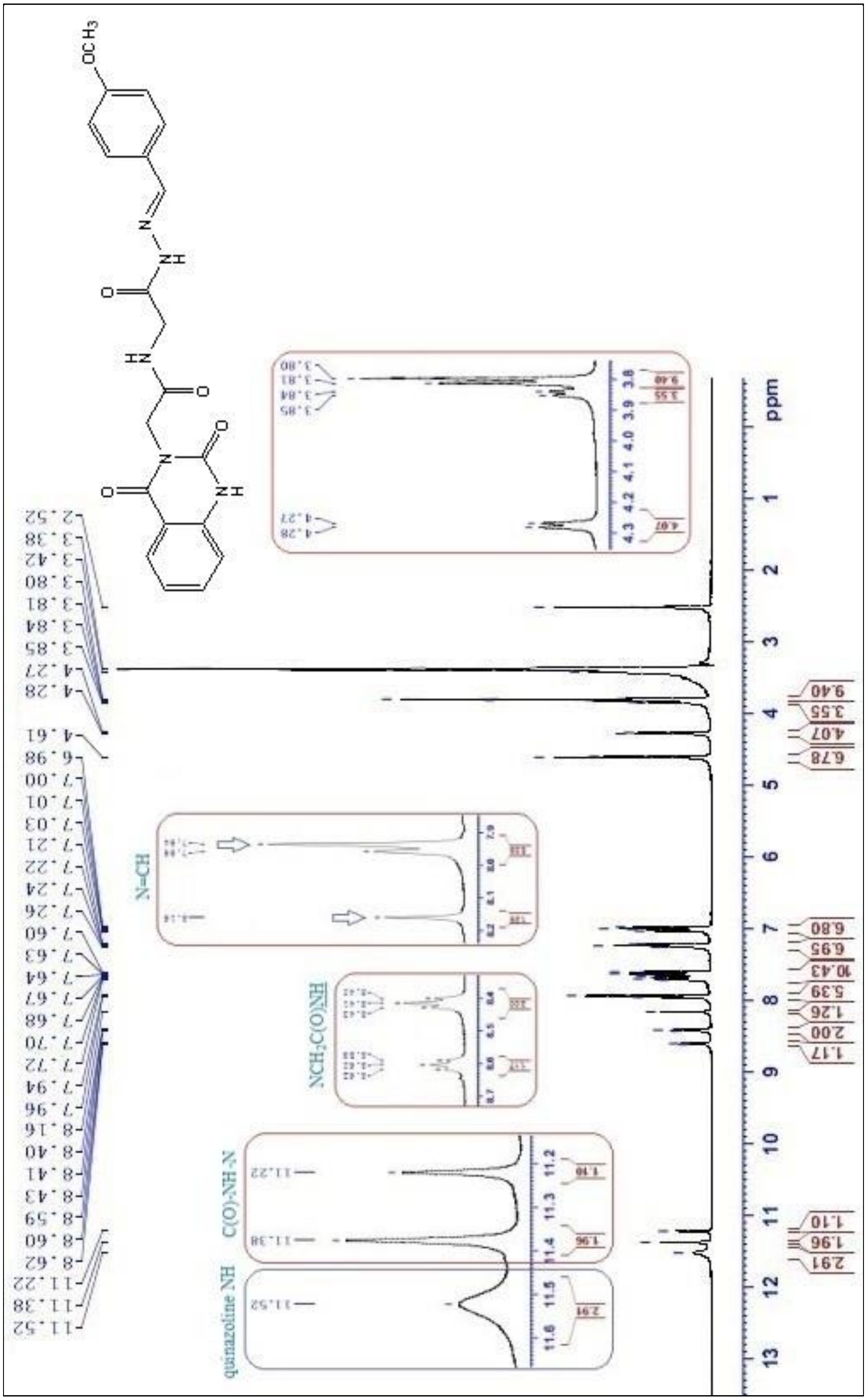

Figure 36: ^{1}H NMR spectrum of compound **4c**.

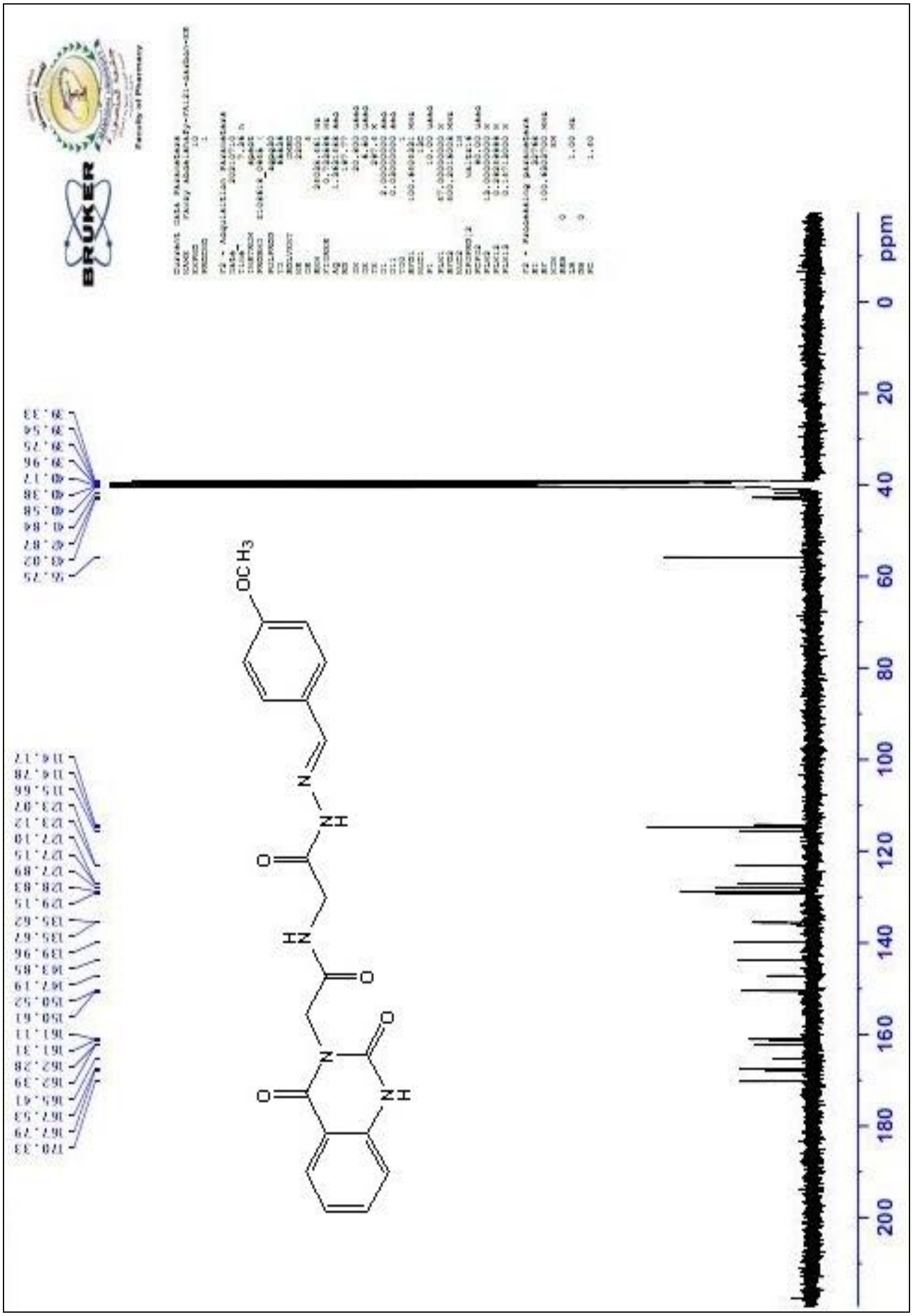

Figure 37: ^{13}C NMR spectrum of compound **4c**.

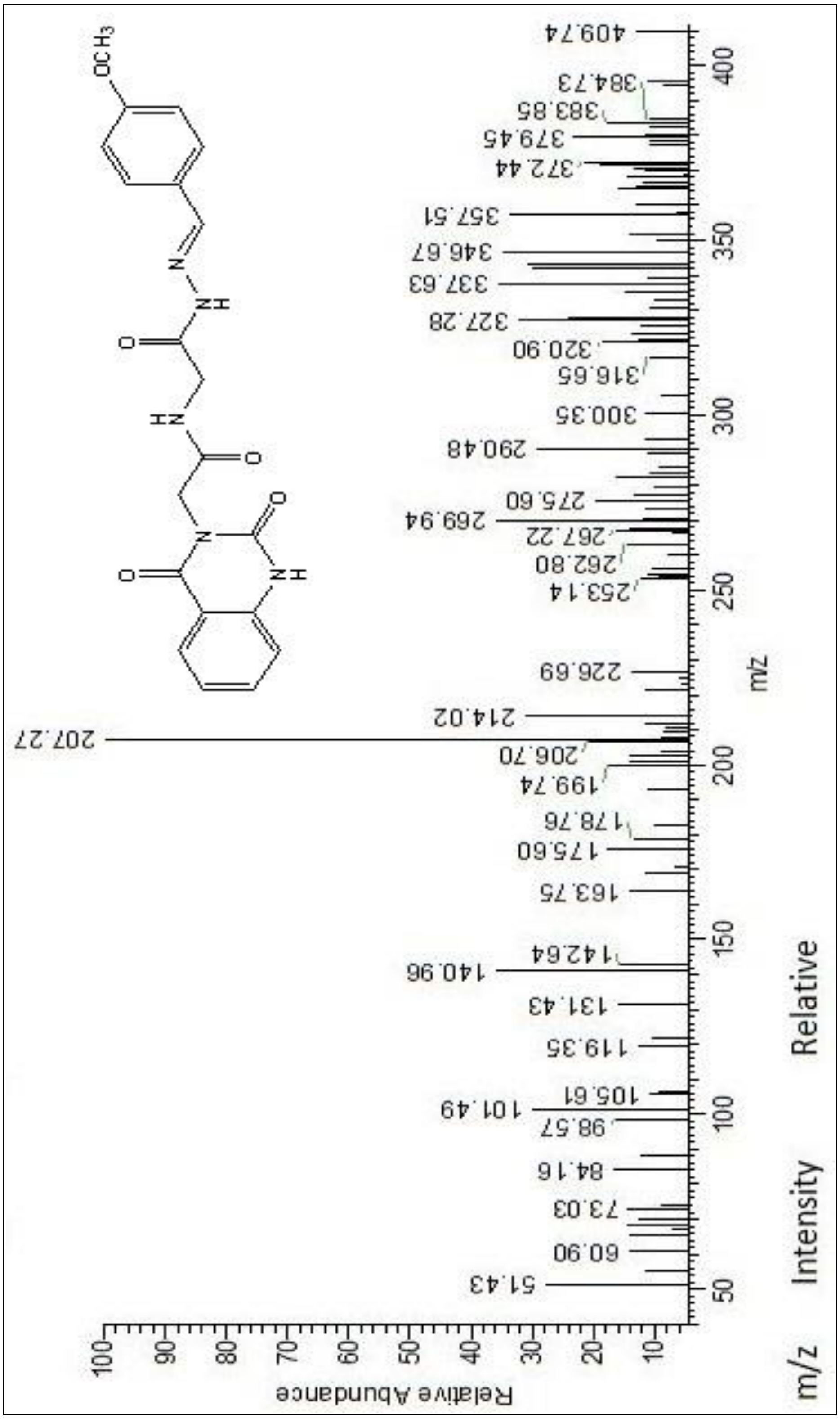

Figure 38: Mass spectrum of compound **4c**.

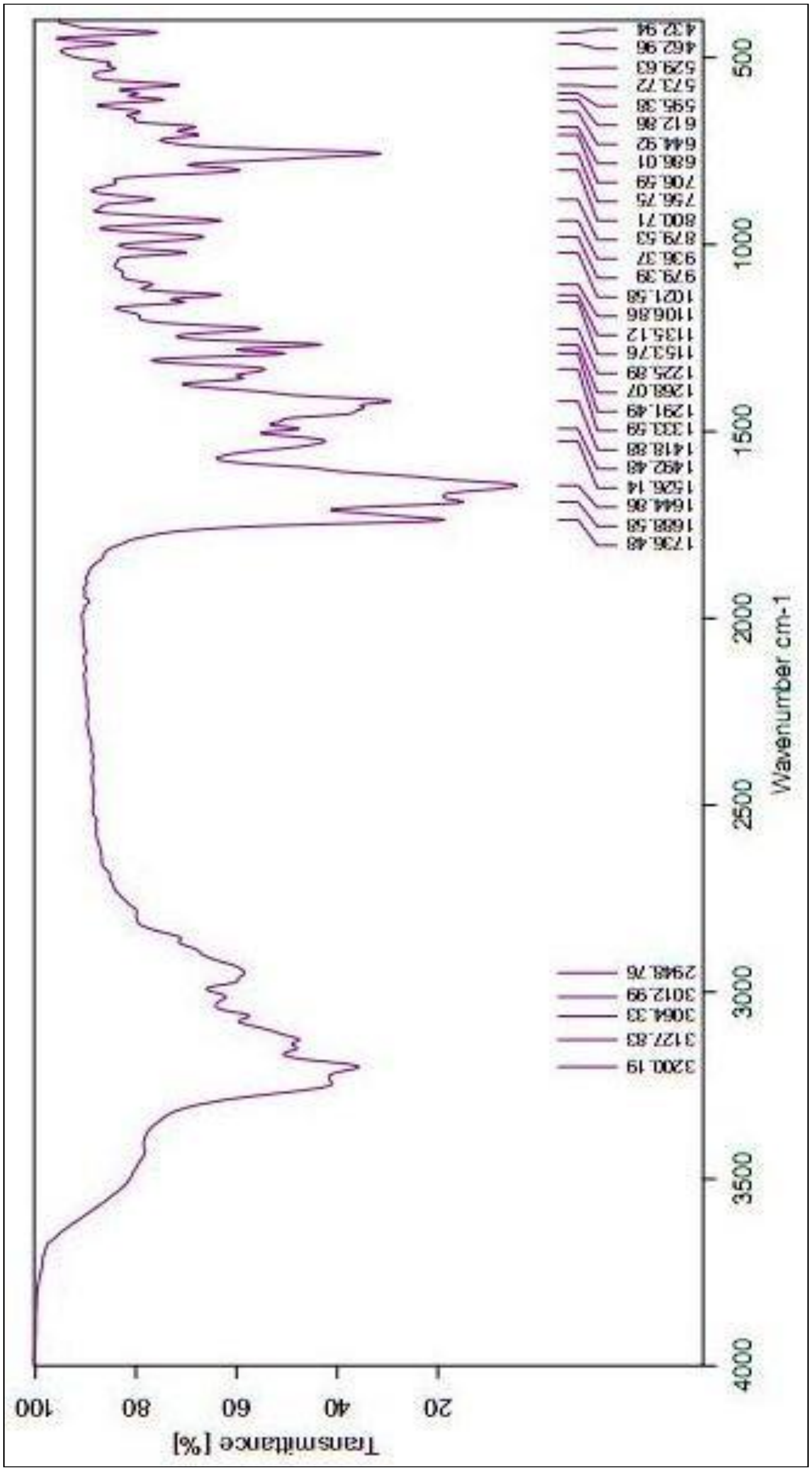

Figure 39: IR spectrum of compound **4d**.

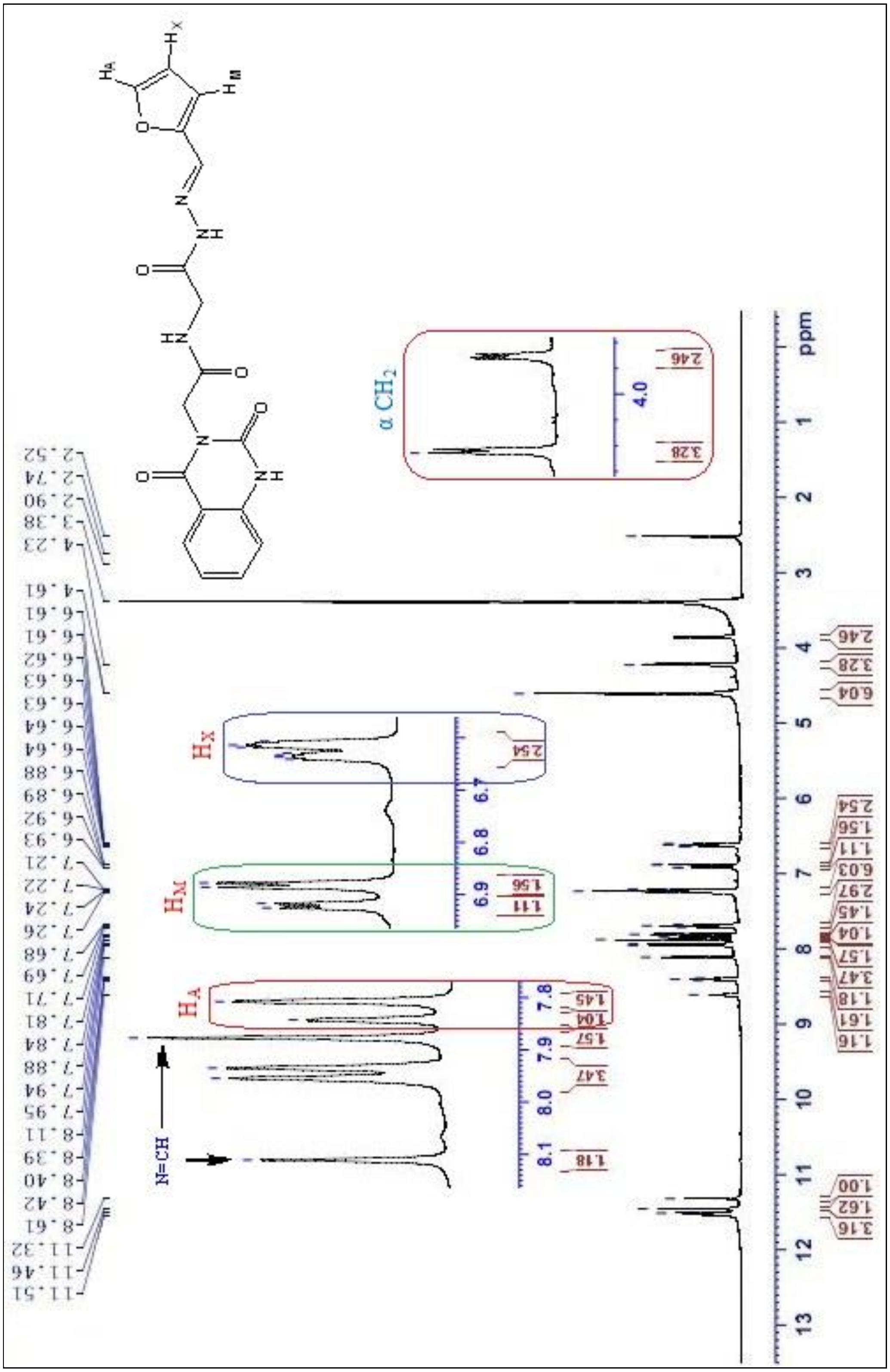

Figure 40: ^{1}H NMR spectrum of compound **4d**.

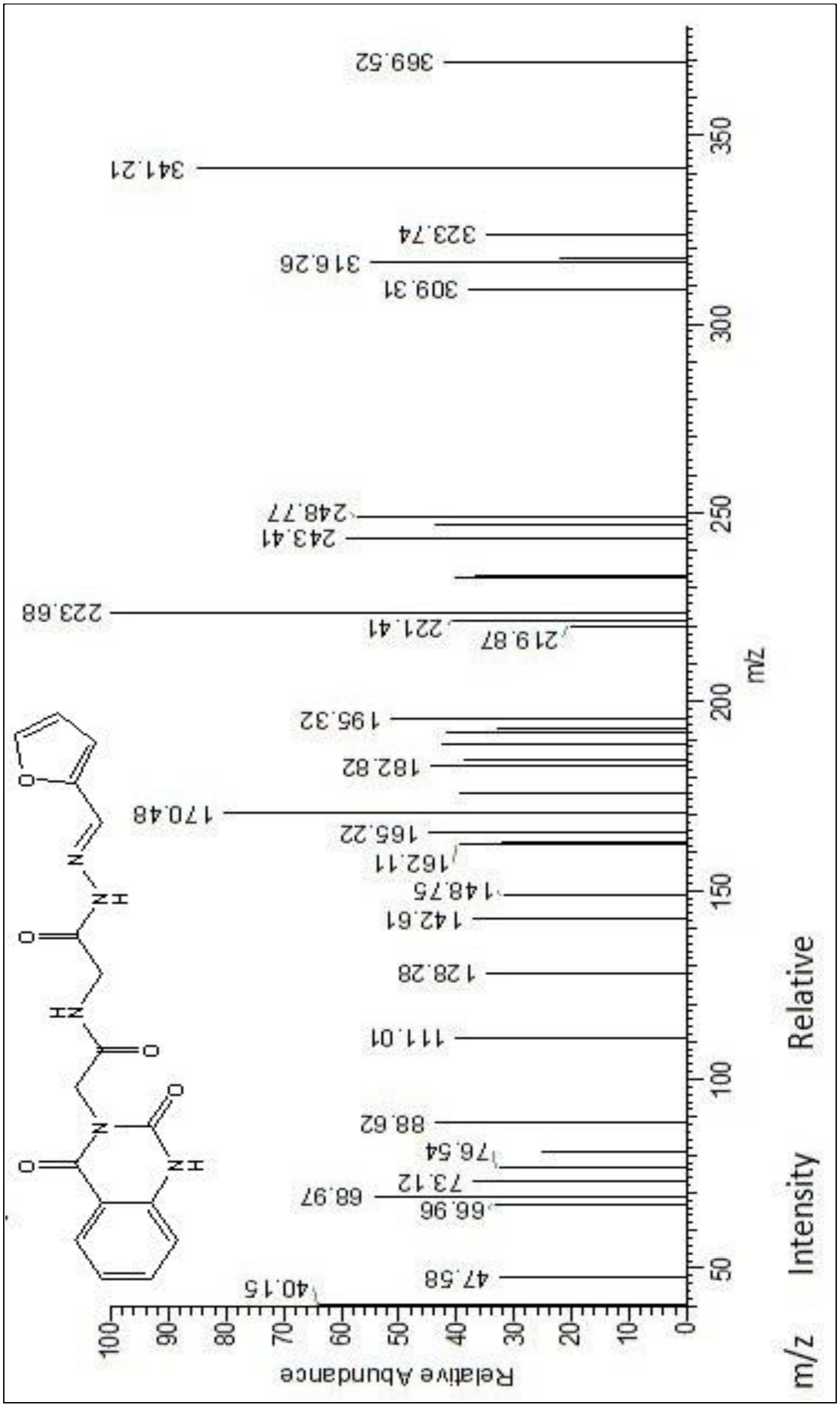

Figure 41: Mass spectrum of compound **4d**.

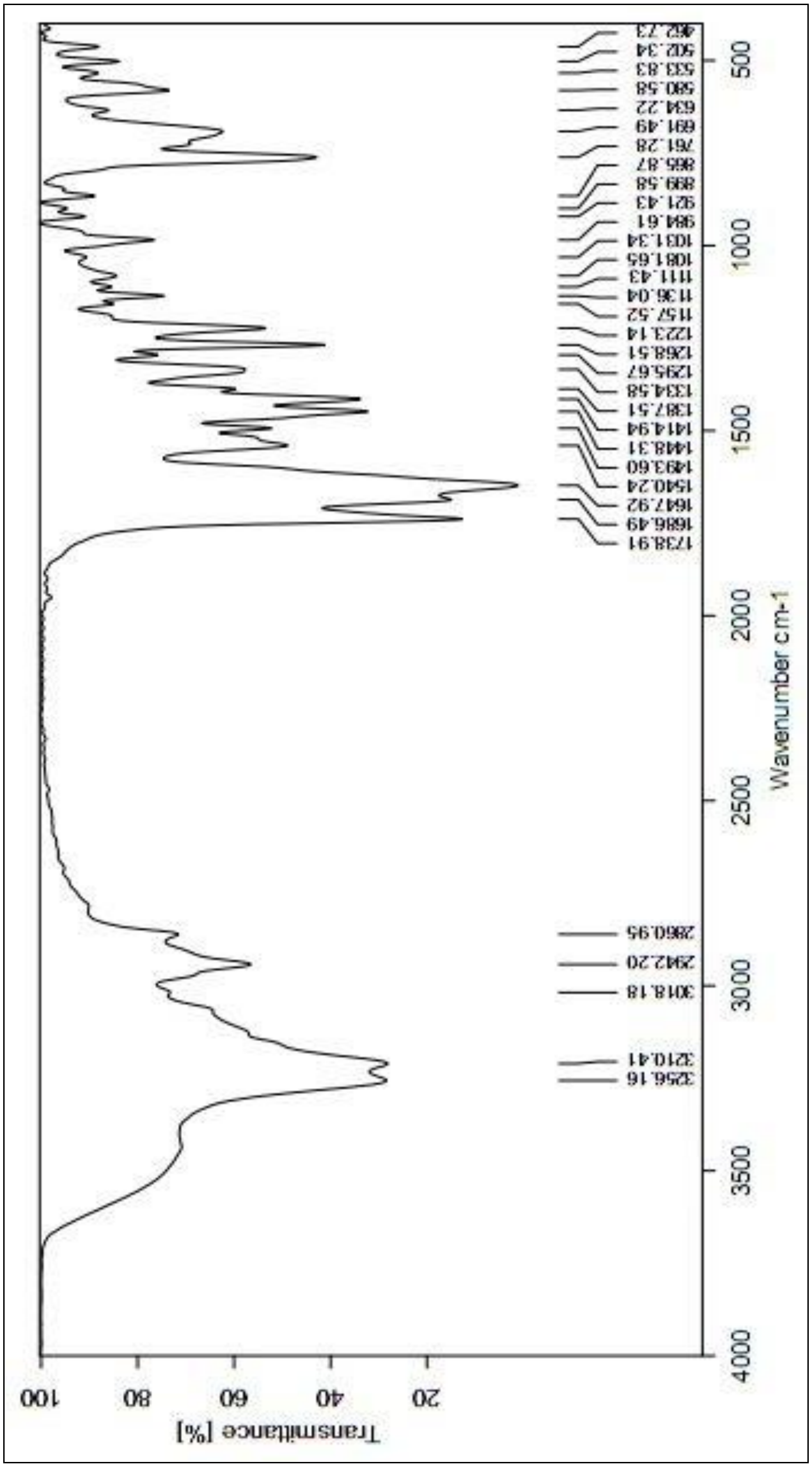

Figure 42: IR spectrum of compound **4e**.

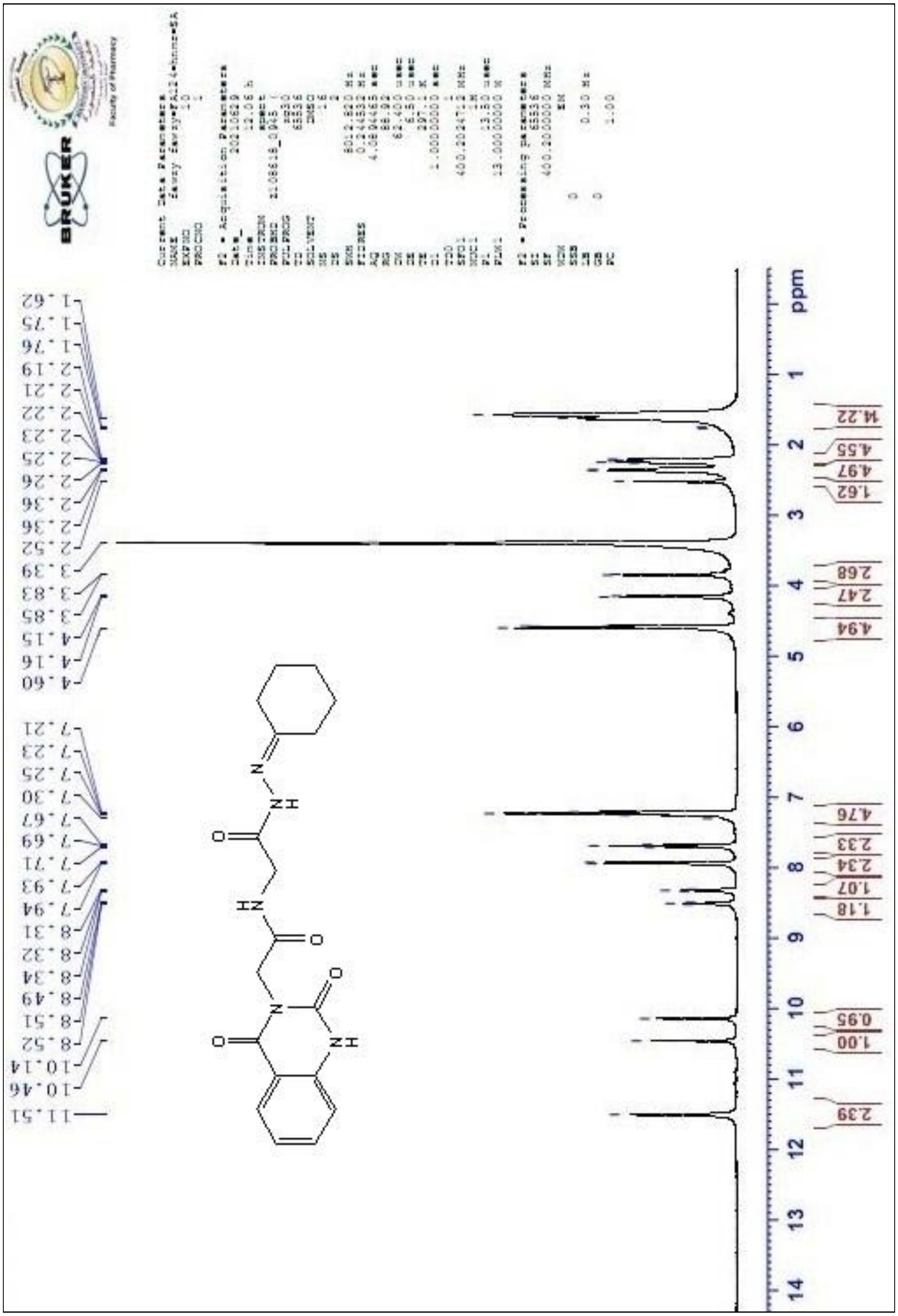

Figure 43: ^{1}H NMR spectrum of compound **4e**.

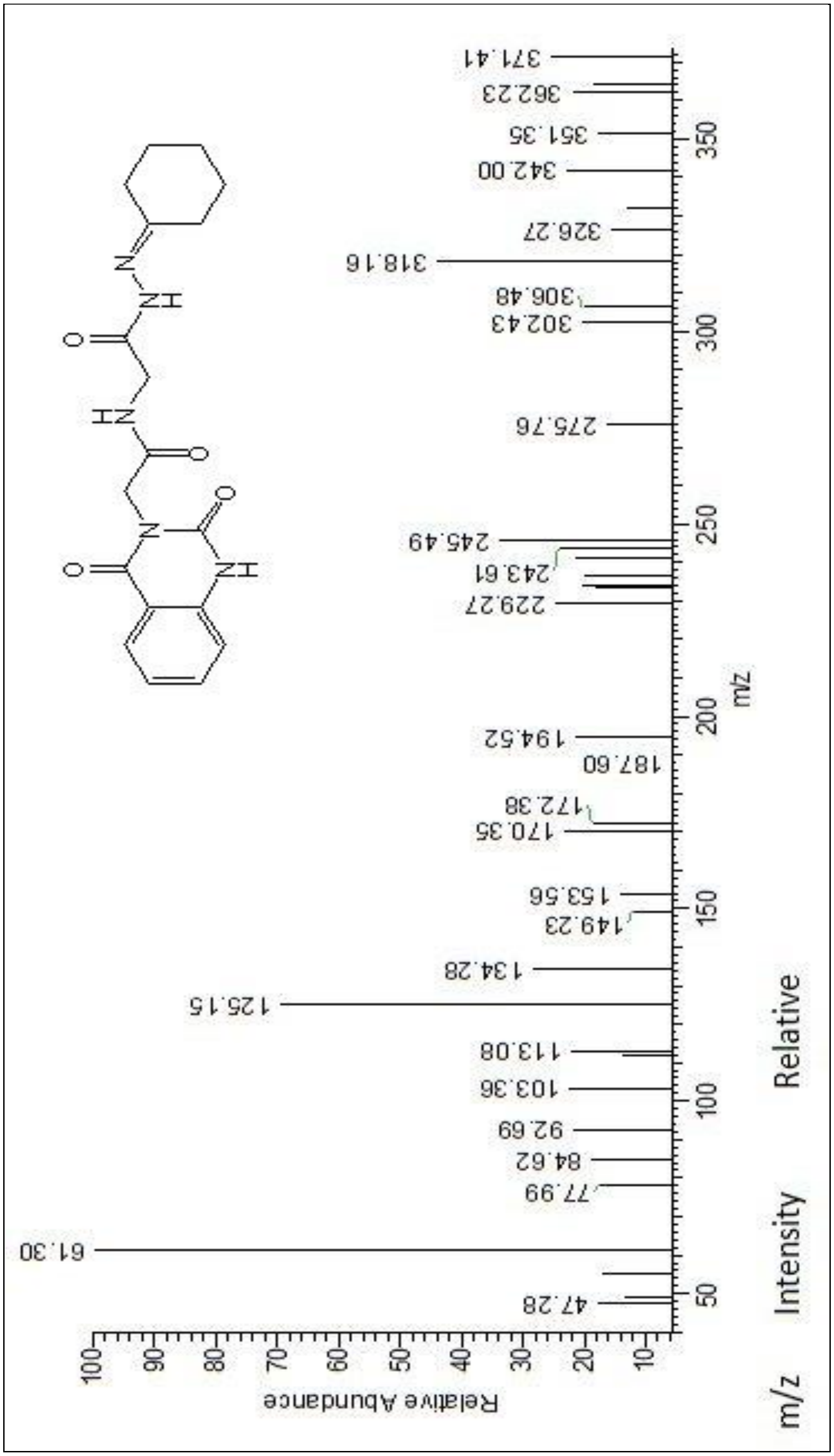

Figure 44: Mass spectrum of compound **4e**.

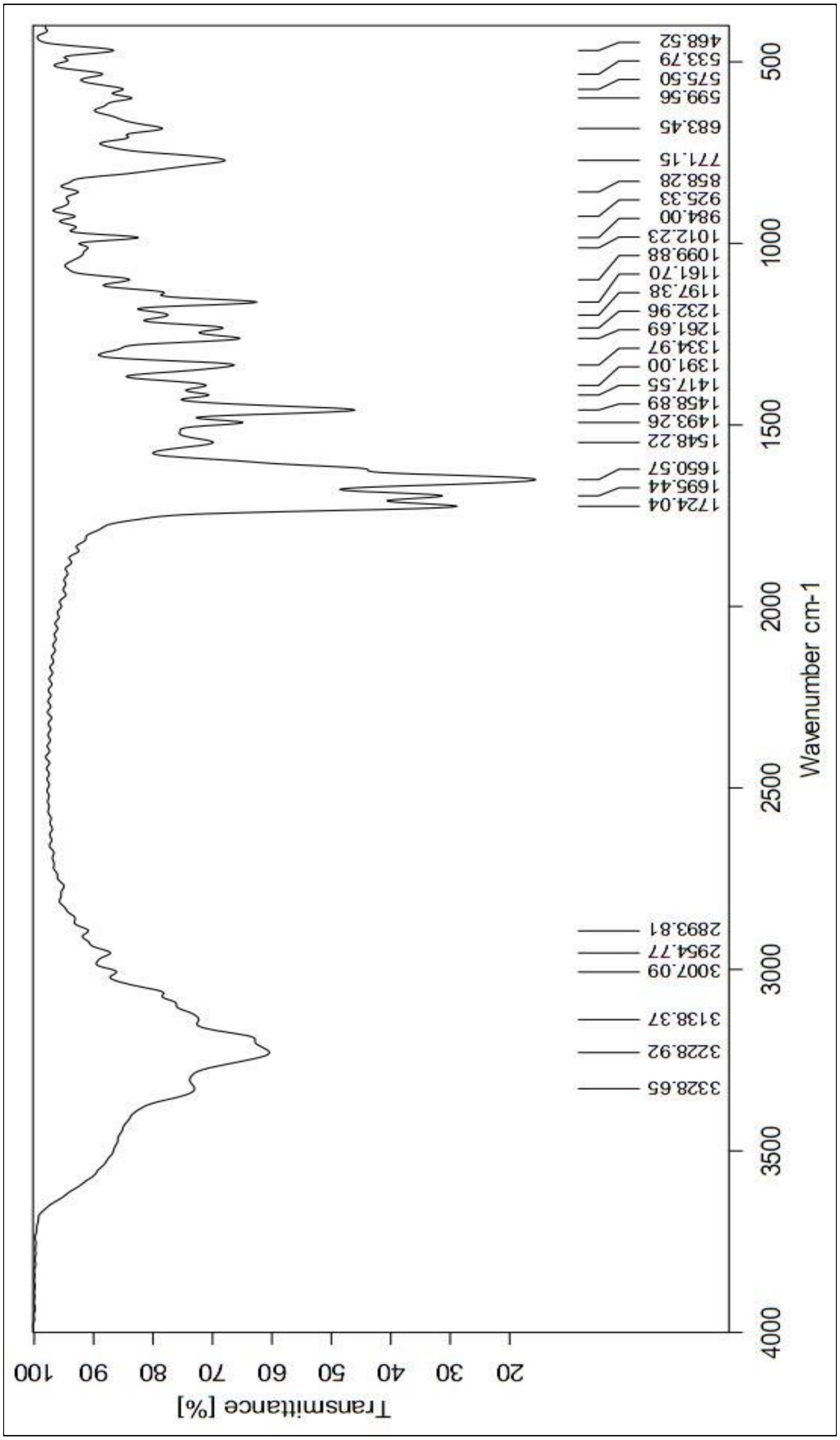

Figure 45: IR spectrum of compound **4f**.

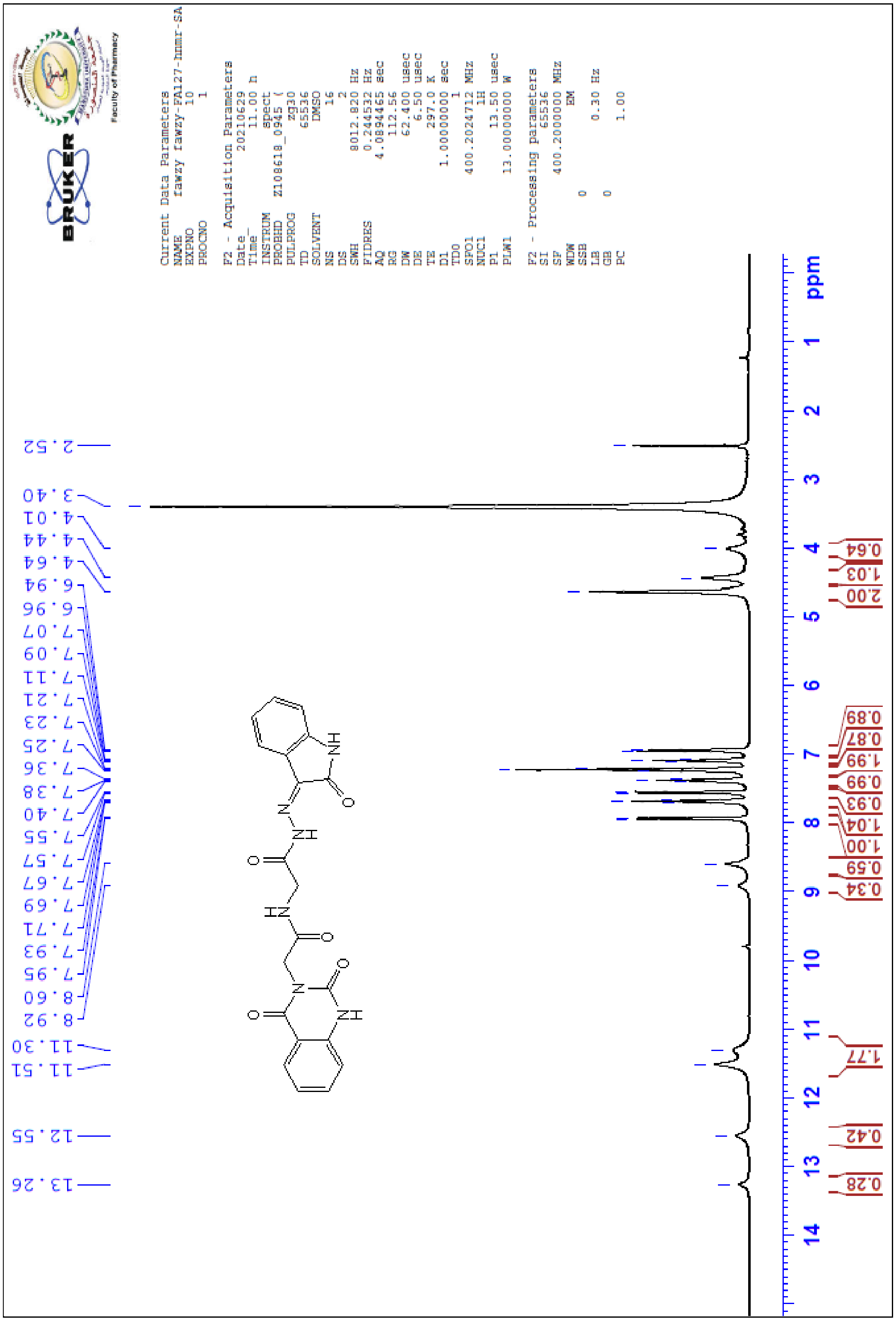

Figure 46: ^{1}H NMR spectrum of compound **4f**.

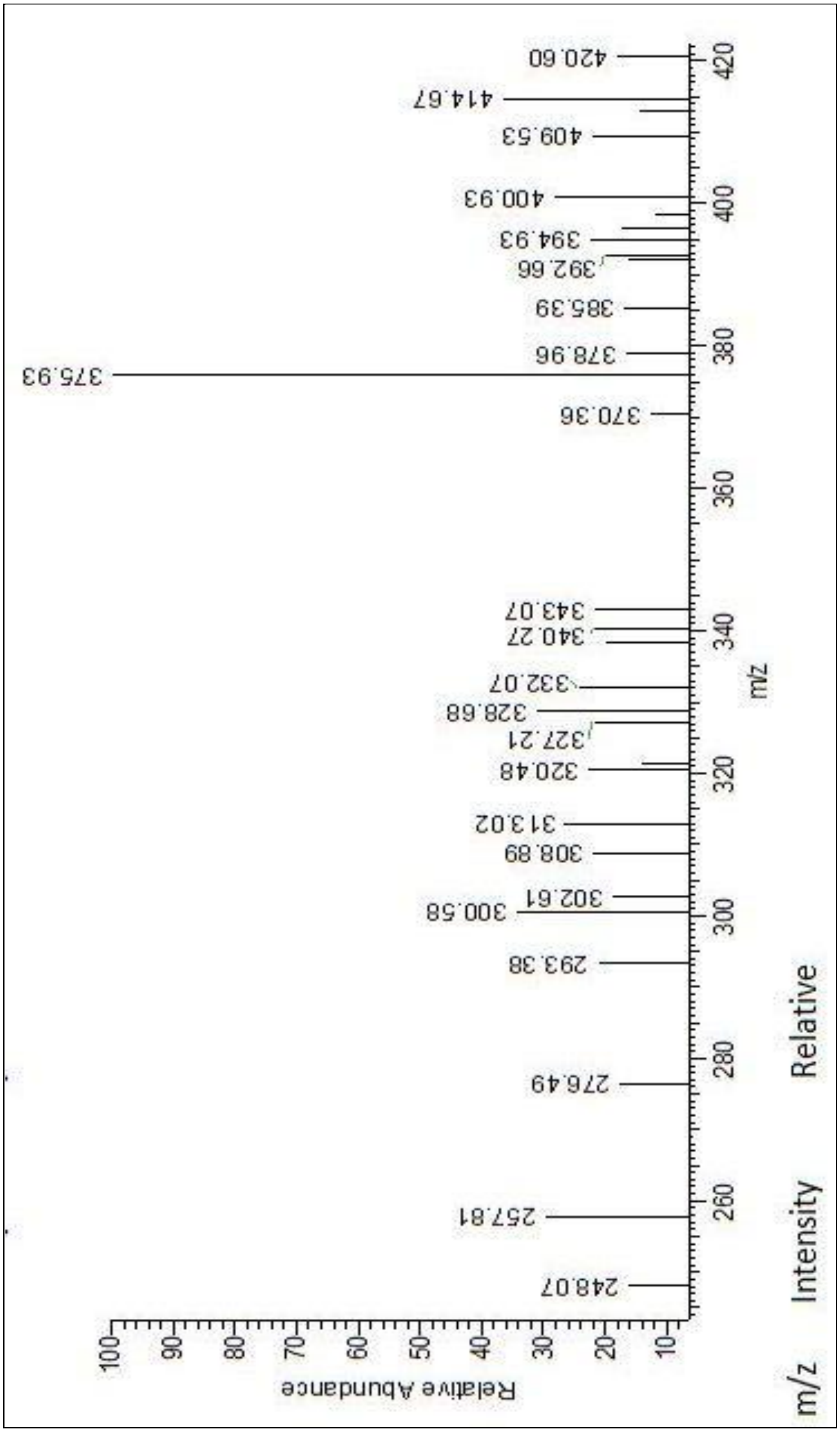

Figure 47: Mass spectrum of compound **4f**.

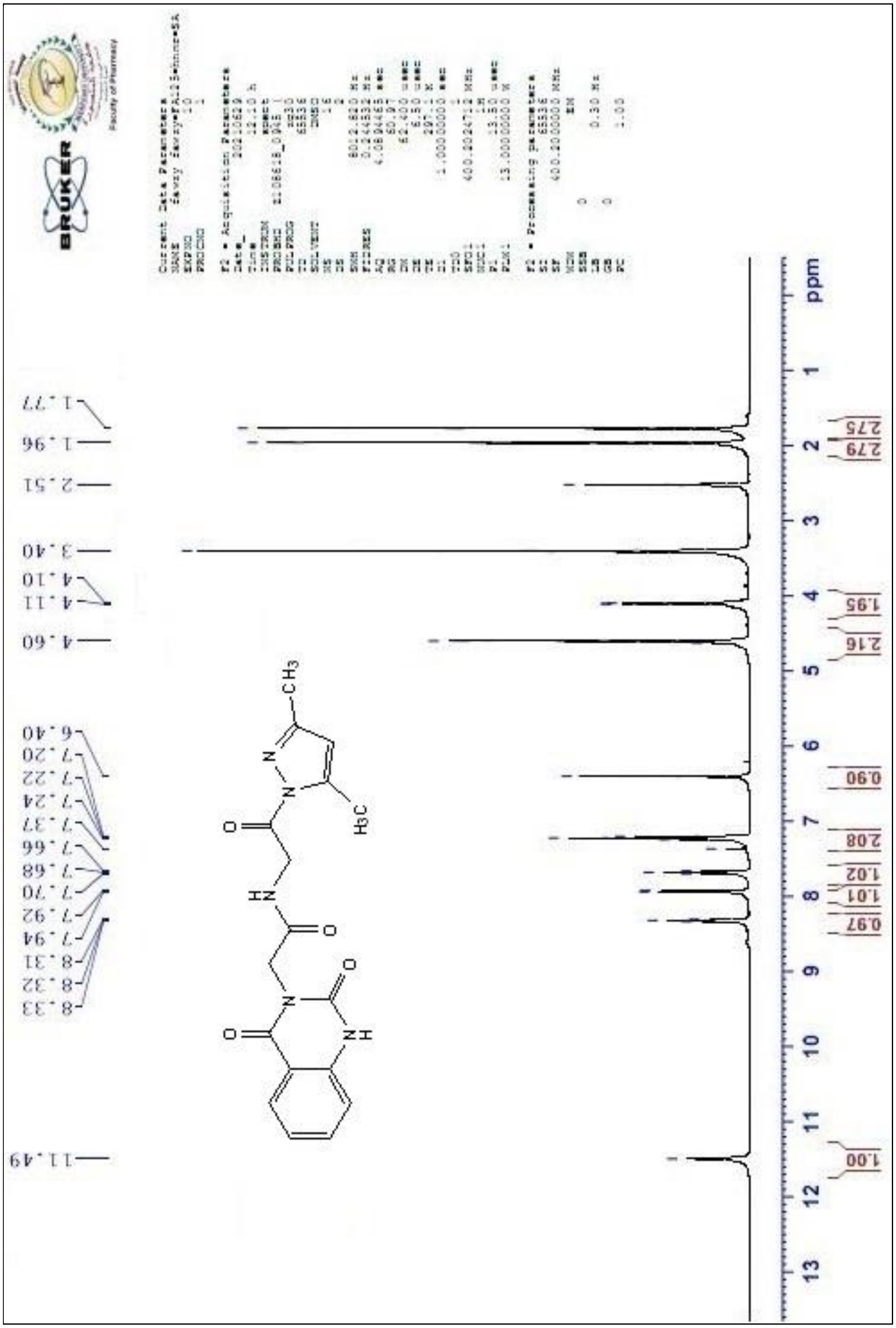

Figure 48: ^{1}H NMR spectrum of compound **4g**.

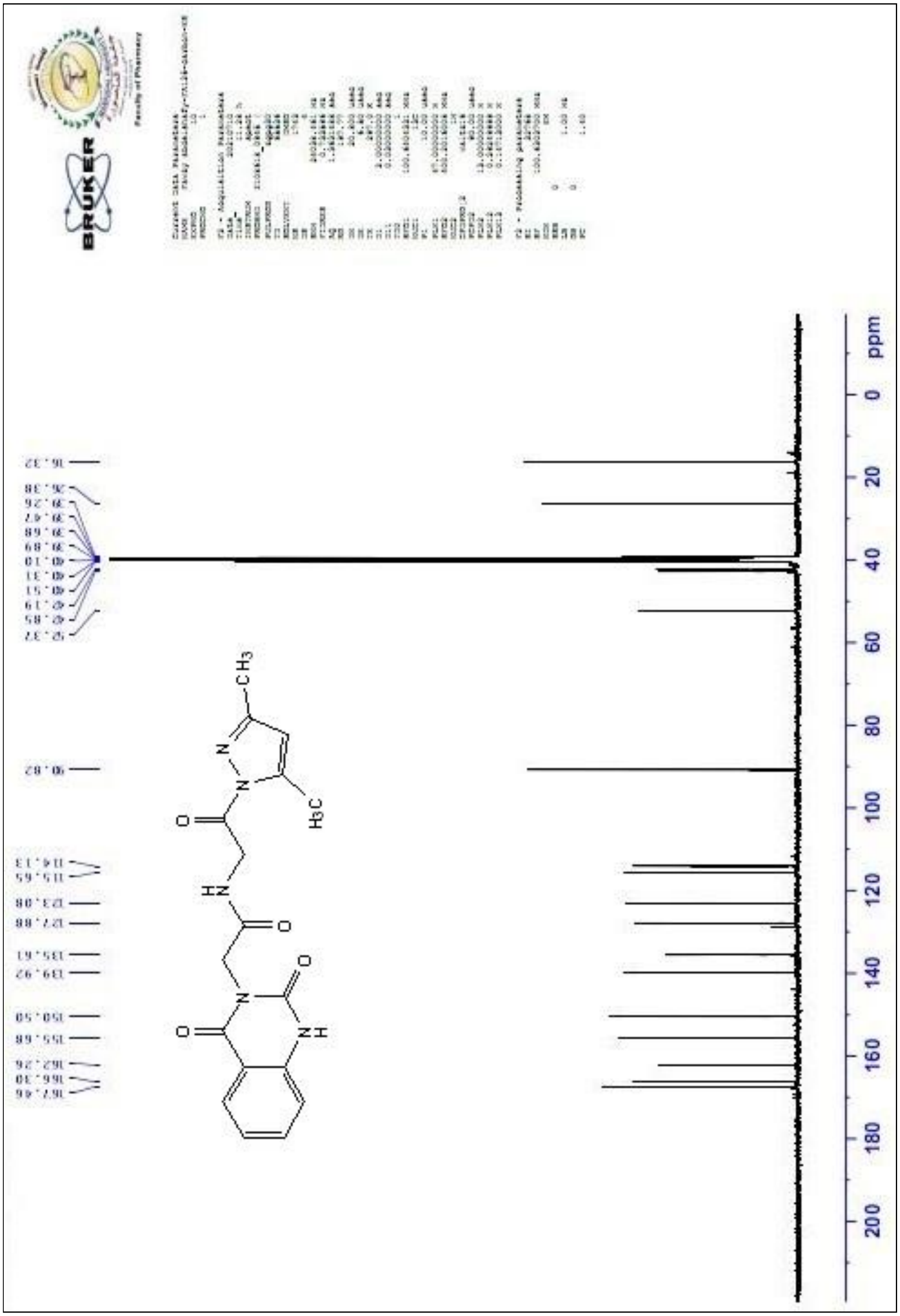

Figure 49: ^{13}C NMR spectrum of compound **4g**.

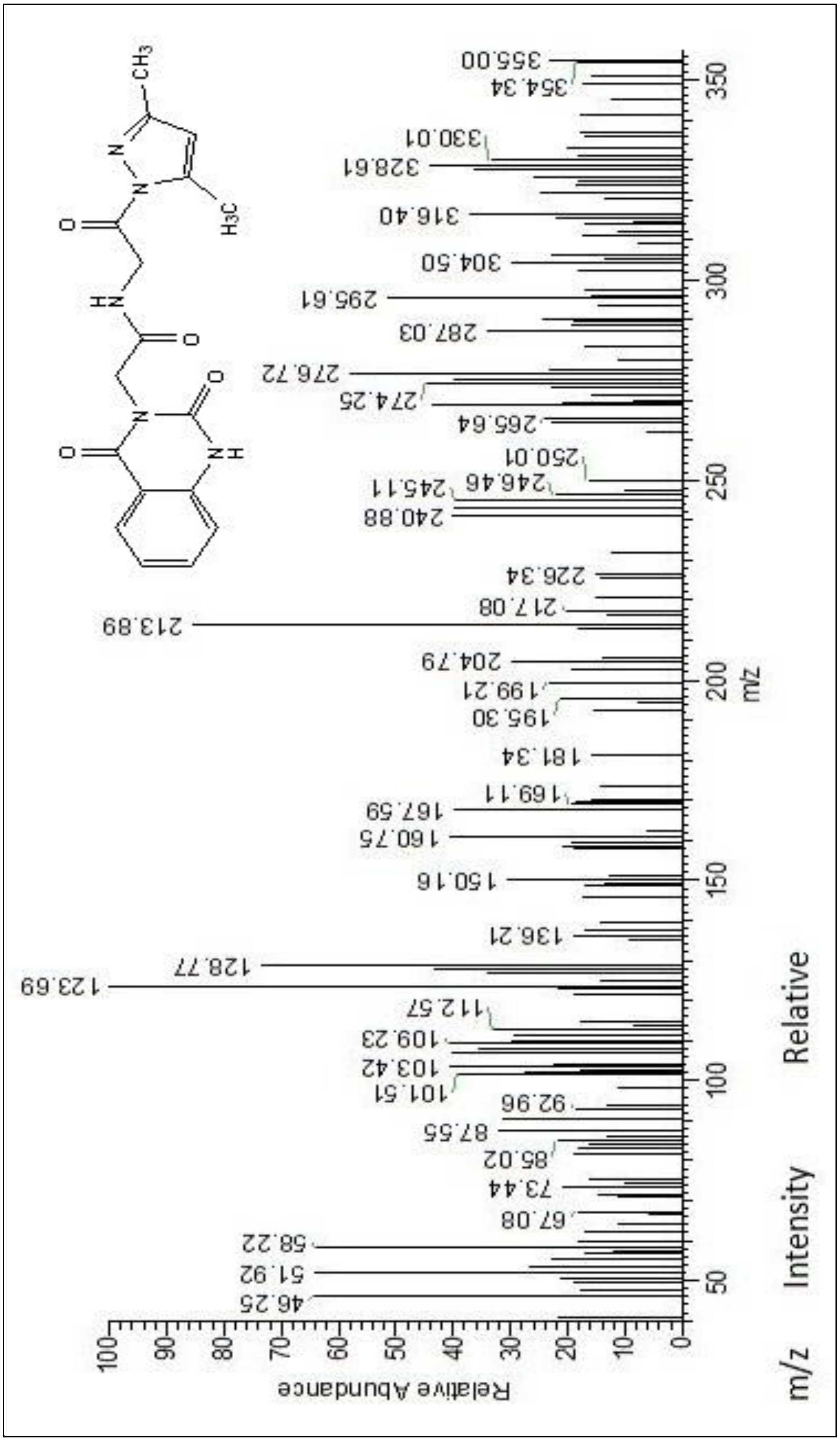

Figure 50: Mass spectrum of compound **4g**.

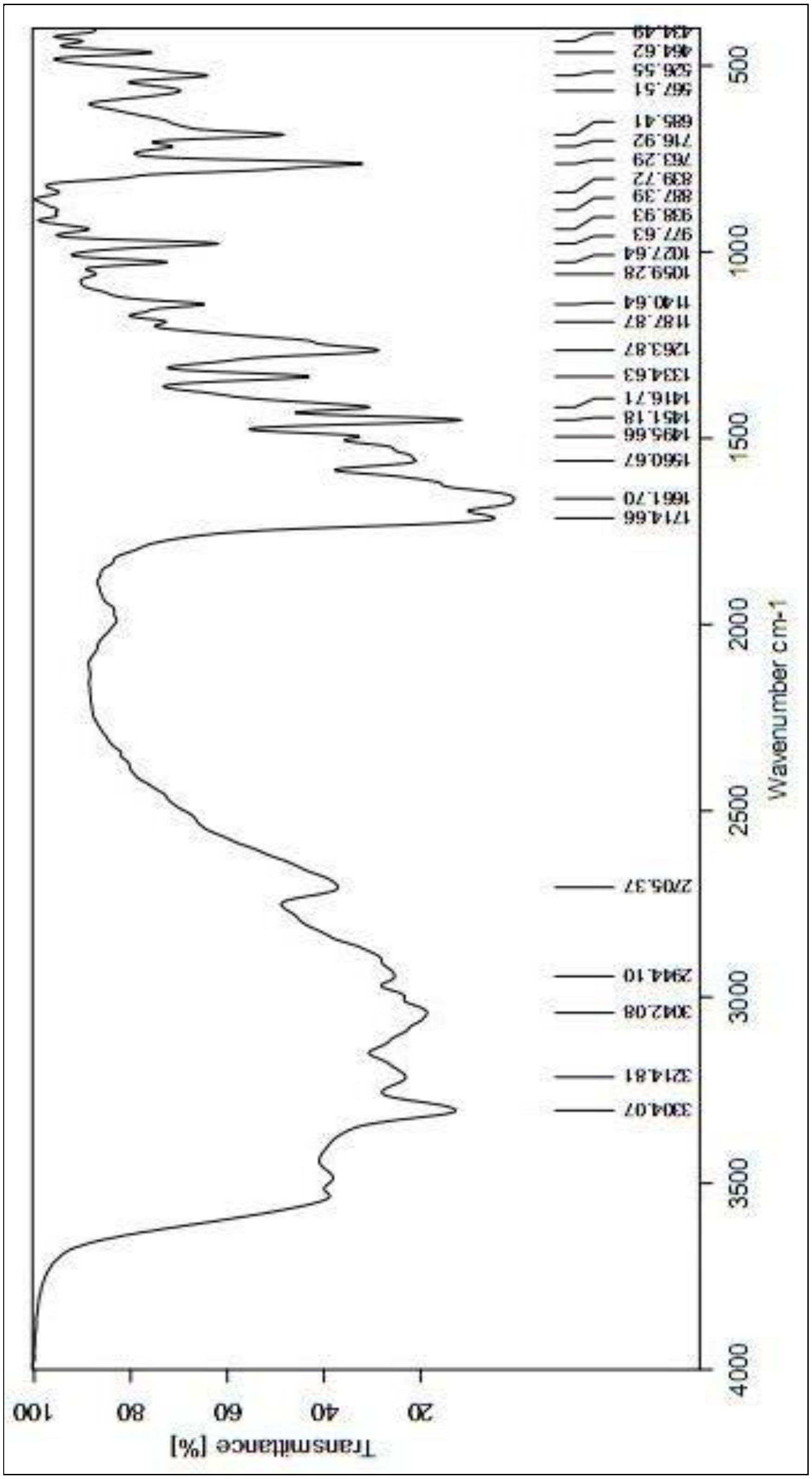
Transmittance [%]
Wavenumber cm-1
3304.07
3214.81
3042.08
2944.10
2705.37
1714.66
1661.70
1560.67
1495.66
1451.18
1416.71
1334.63
1263.87
1187.87
1140.64
1059.28
1027.64
977.63
938.93
887.39
839.72
763.29
716.92
685.41
567.51
526.55
464.62
434.49

Figure 51: IR spectrum of compound **4h**.

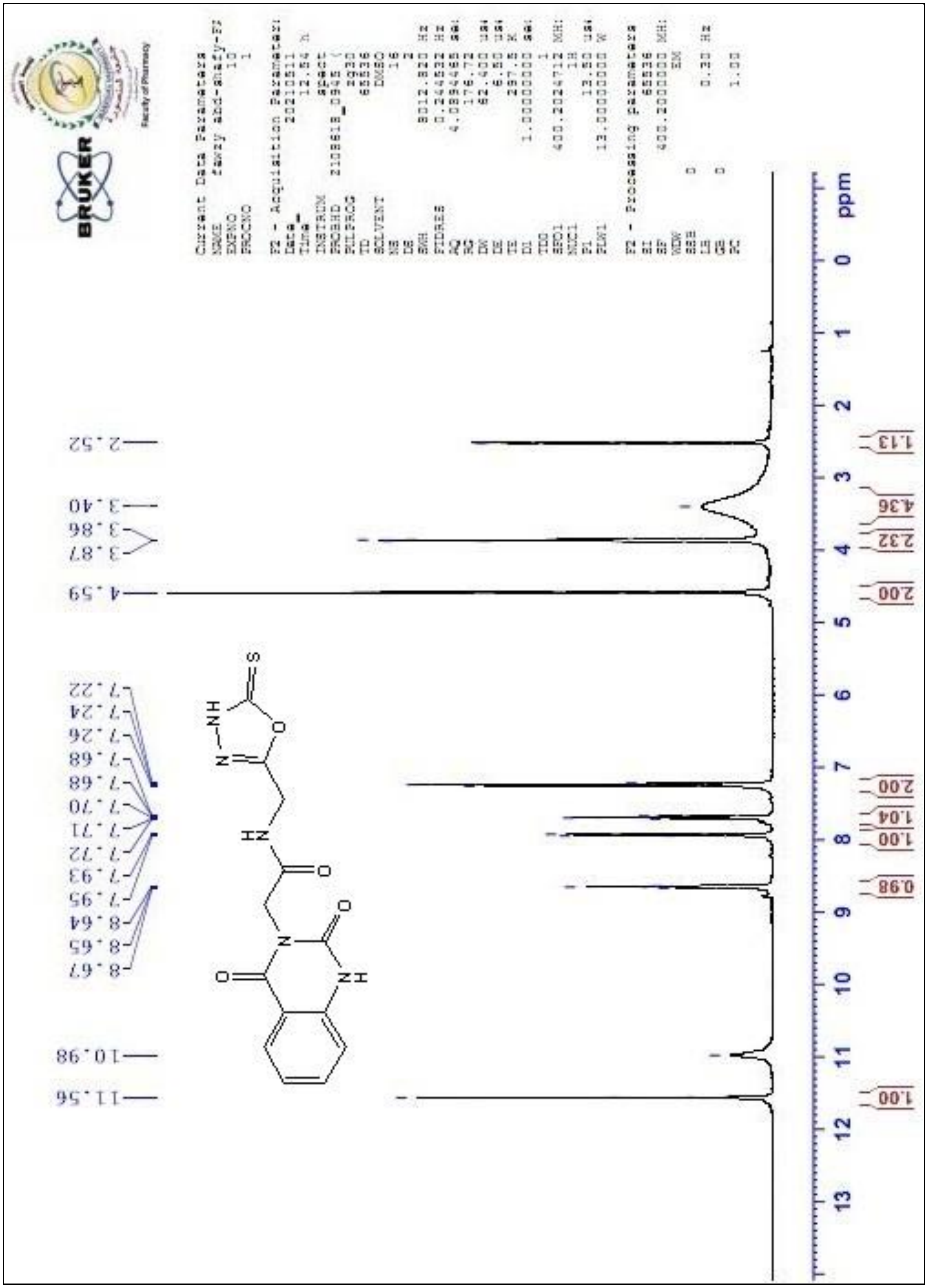

Figure 52: ^{1}H NMR spectrum of compound **4h**.

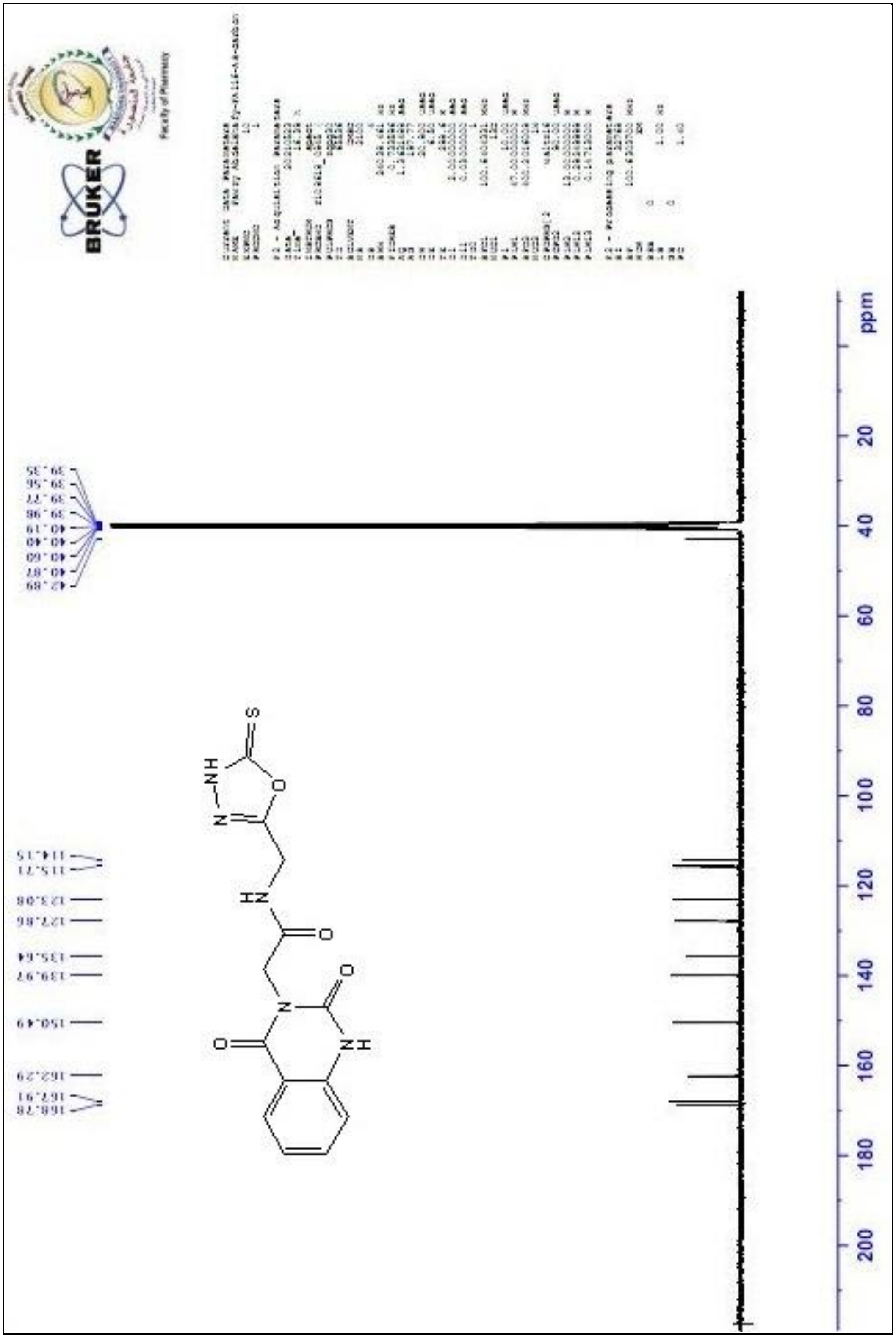

Figure 53: ^{13}C NMR spectrum of compound **4h**.

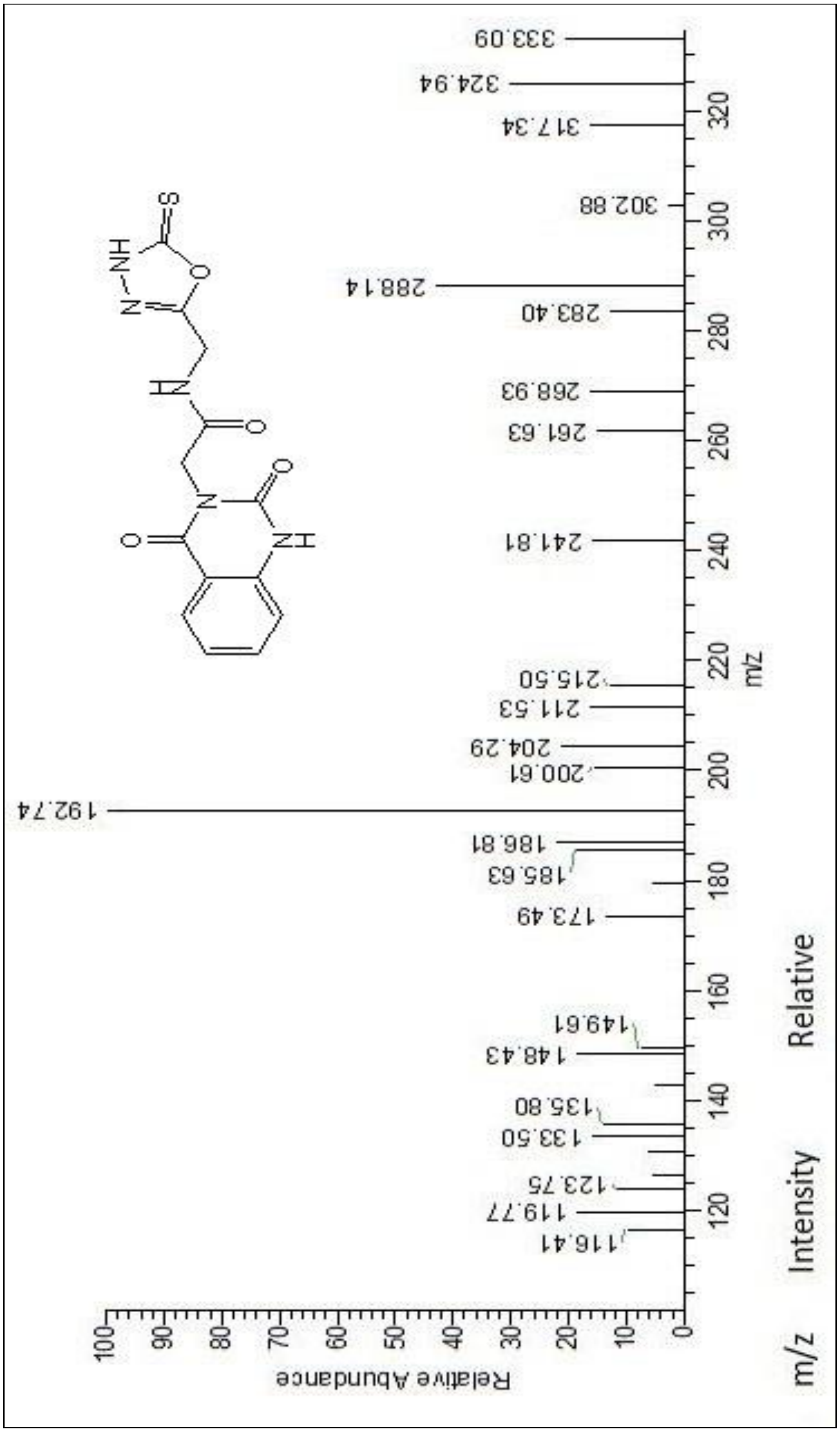

Figure 54: Mass spectrum of compound **4h**.

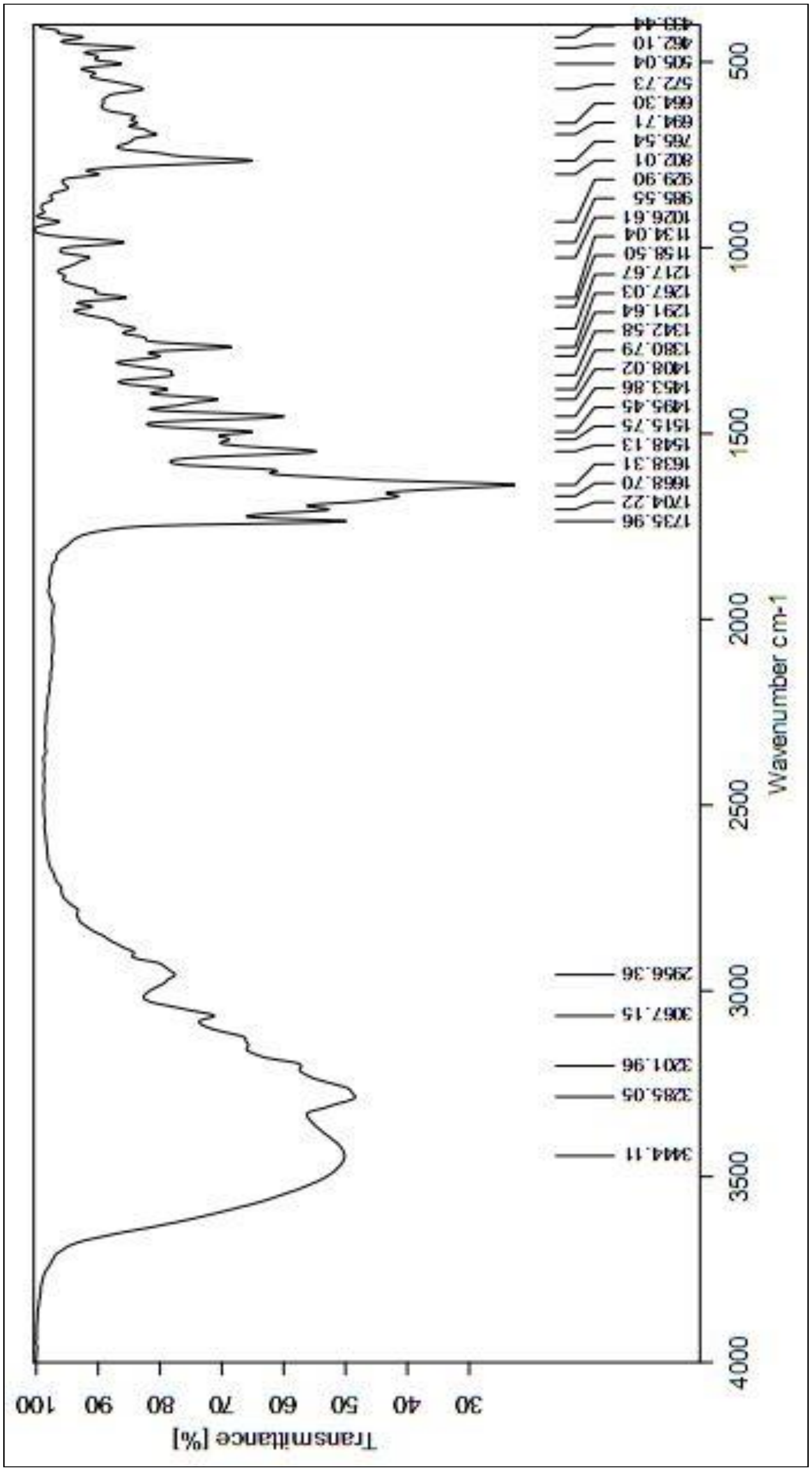

Figure 55: IR spectrum of compound **4i**.

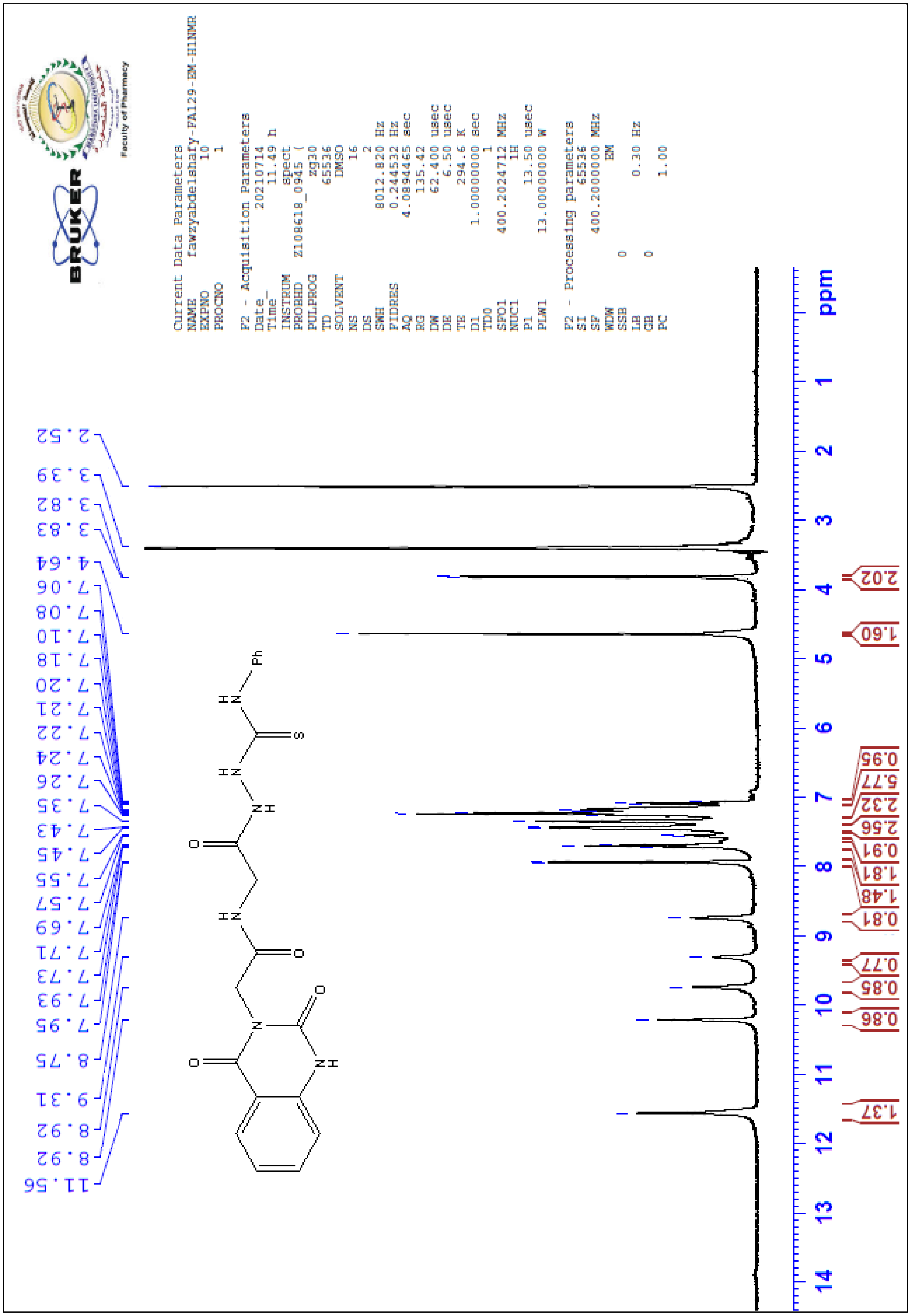

Figure 56: ^{1}H NMR spectrum of compound **4i**.

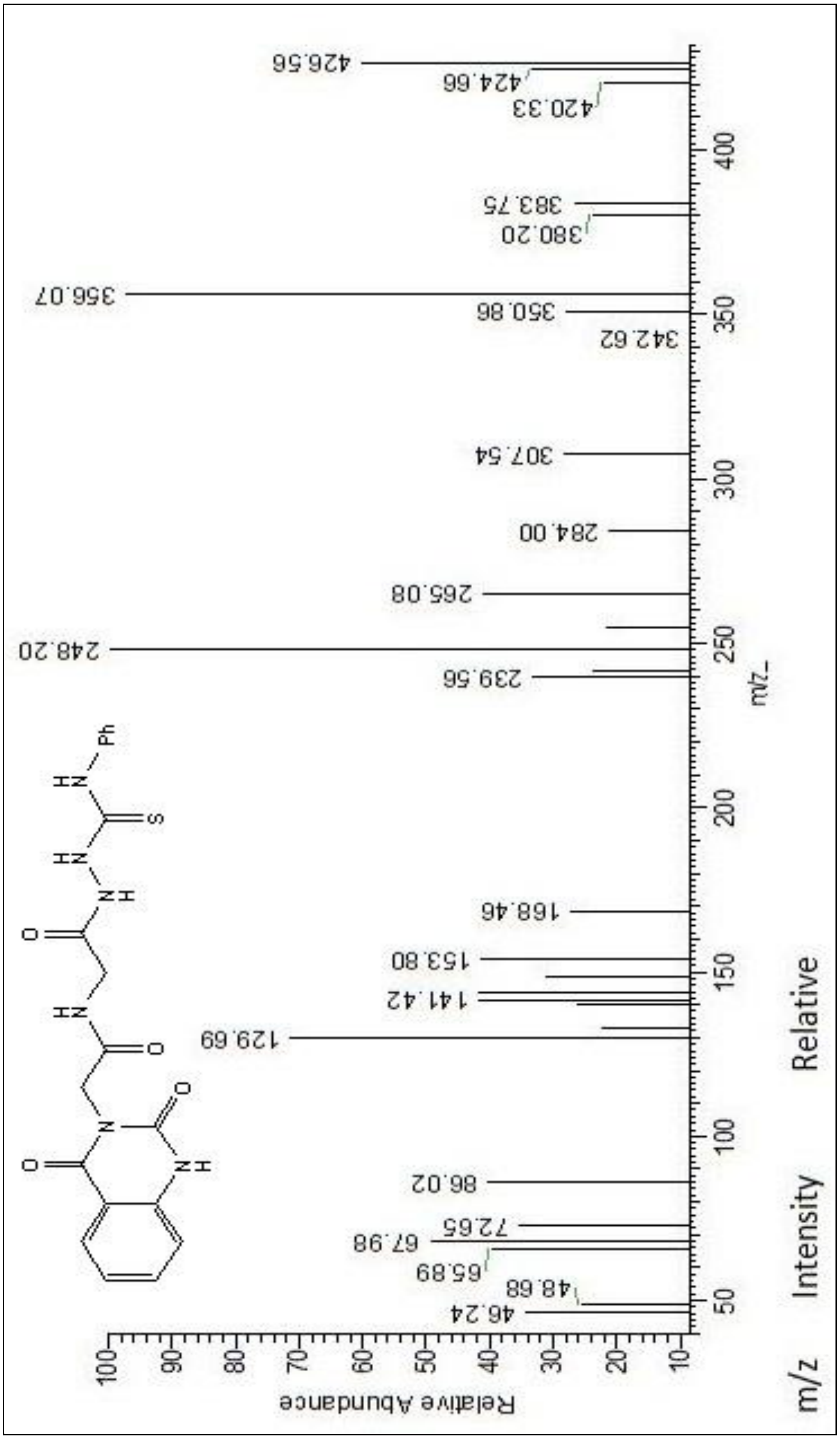

Figure 57: Mass spectrum of compound **4i**.

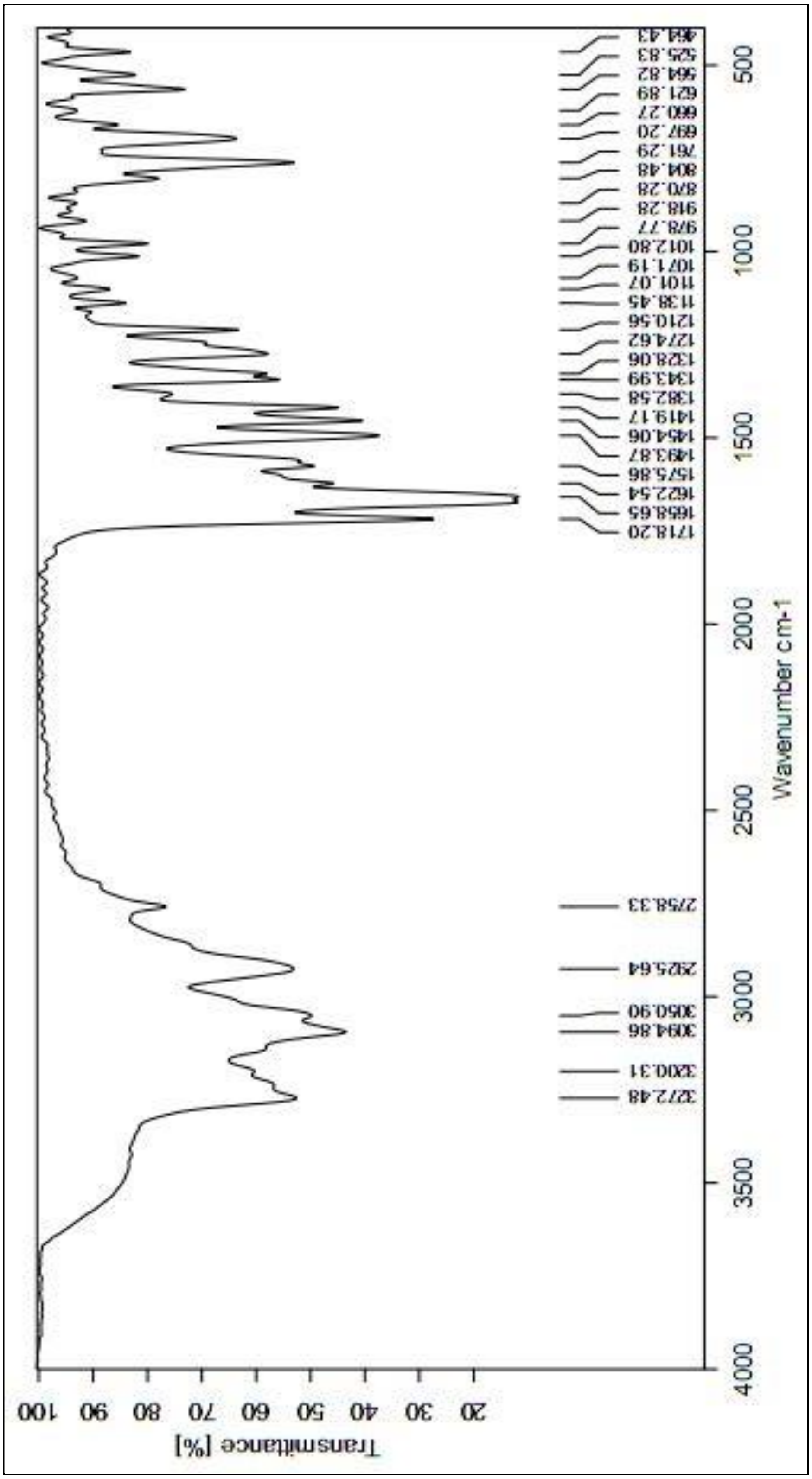

Figure 58: IR spectrum of compound **4j**.

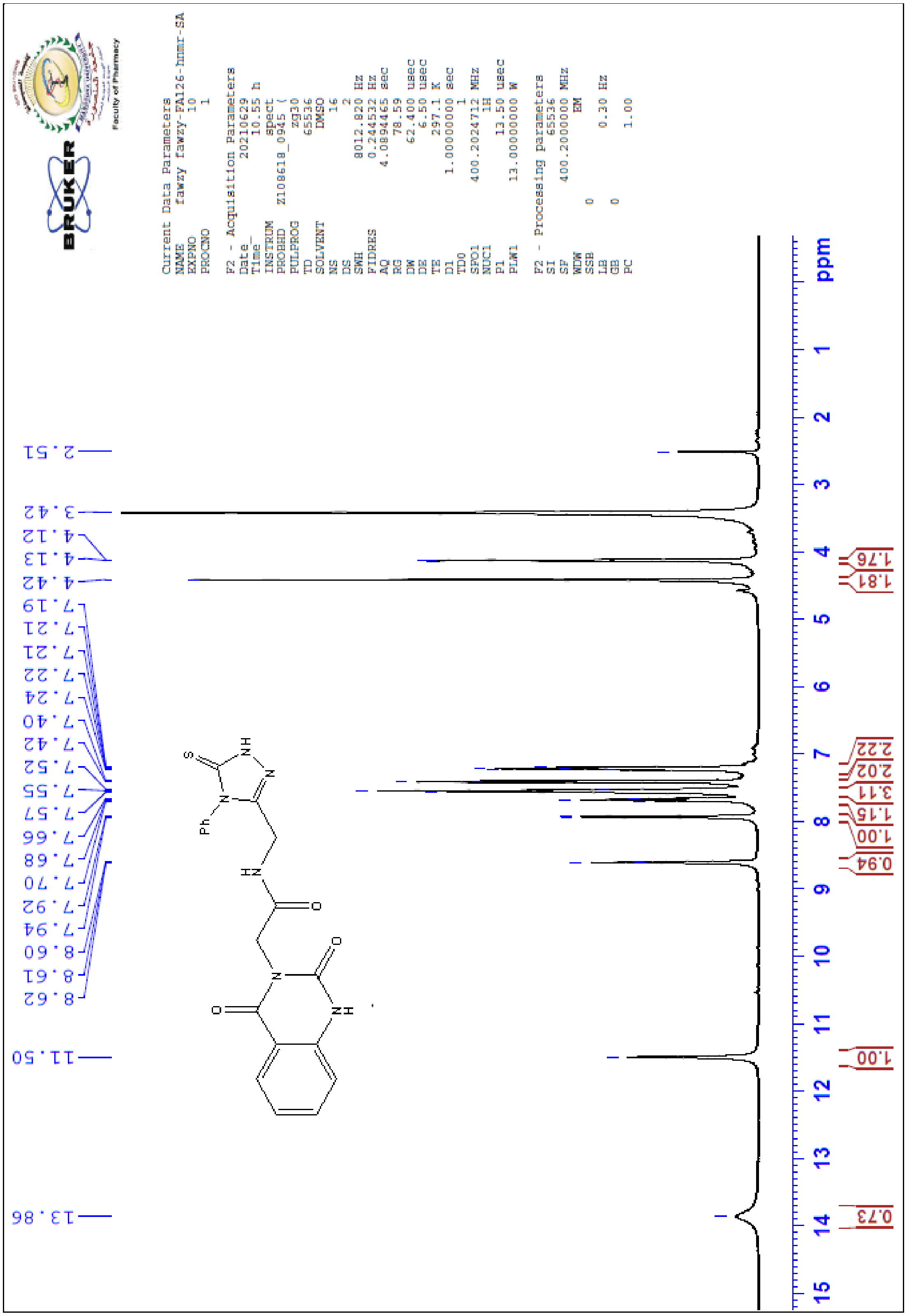

13.86
11.50
8.62 8.61 8.60 7.94 7.92 7.70 7.68 7.66 7.57 7.55 7.52 7.42 7.40 7.24 7.22 7.21 7.21 7.19
4.42 4.13 4.12
3.42
2.51
BRUKER
Faculty of Pharmacy
Current Data Parameters
NAME fawzy fawzy-FA126-hnmr-SA
EXPNO 10
PROCNO 1
F2 - Acquisition Parameters
Date_ 20210629
Time_ 10.55 h
INSTRUM spect
PROBHD Z108618_0945 (
PULPROG zg30
TD 65536
SOLVENT DMSO
NS 16
DS 2
SWH 8012.820 Hz
FIDRES 0.244532 Hz
AQ 4.0894465 sec
RG 78.59
DW 62.400 usec
DE 6.50 usec
TE 297.1 K
D1 1.00000000 sec
TD0 1
SFO1 400.2024712 MHz
NUC1 1H
P1 13.50 usec
PLW1 13.00000000 W
F2 - Processing parameters
SI 65536
SF 400.2000000 MHz
WDW EM
SSB 0
LB 0.30 Hz
GB 0
PC 1.00
Ph
S
NH
N
H
O
15 14 13 12 11 10 9 8 7 6 5 4 3 2 1 ppm
0.73 1.00 0.94 1.00 1.15 3.11 2.02 2.22 1.81 1.76

Figure 59: ^{1}H NMR spectrum of compound **4j**.

Table 1: ^{1}H NMR chemical shifts and ratio of *cis*/*trans* *N*-acylhydrazones **4a-d**.

**compound**	**Chemical Shift δ (ppm)**								
	α-CH_2		N=CH		$NCH_2C(O)$<u>NH</u>		C(O)-NH-N		Ratio of *cis*:*trans*
	cis	*trans*	*cis*	*trans*	*cis*	*trans*	*cis*	*trans*	
**4a**	4.30	3.86	7.99	8.22	8.45	8.63	11.52	11.39	5:3
**4b**	4.34	3.91	8.06	8.29	8.50	8.67	11.80	11.73	2:1
**4c**	4.27	3.84	7.94	8.16	8.41	8.60	11.38	11.22	7:4
**4d**	4.23	3.85	7.88	8.11	8.40	8.61	11.46	11.32	7:5

Table 2: c-Met IC_{50} calculations of compounds **2c, 2f, 4b, 4e, 4g, 4h** and cabozantinib.

**2c**	IC_{50}	conc	log	%inh	T2	T1	ΔT	RFU2	RFU1	ΔRFU	slope	K.Activity
		10	1	92.28	30	0	30	7.72	0	7.72	3.3333	9.26409
		1	0	73.28	30	0	30	26.72	0	26.72	3.3333	32.0643
		0.1	-1	55.94	30	0	30	44.06	0	44.06	3.3333	52.8725
		0.01	-2	27.36	30	0	30	72.64	0	72.64	3.3333	87.1689
EC				0	30	0	30	100	0	100	3.3333	120
**2f**	IC_{50}	conc	log	%inh	T2	T1	ΔT	RFU2	RFU1	ΔRFU	slope	K.Activity
		10	1	88.98	30	0	30	11.02	0	11.02	3.3333	13.2241
		1	0	70.59	30	0	30	29.41	0	29.41	3.3333	35.2924
		0.1	-1	44.08	30	0	30	55.92	0	55.92	3.3333	67.1047
		0.01	-2	22.84	30	0	30	77.16	0	77.16	3.3333	92.5929
EC				0	30	0	30	100	0	100	3.3333	120

**4b**	IC_{50}	conc	log	%inh	T2	T1	ΔT	RFU2	RFU1	ΔRFU	slope	K.Activity
		10	1	93.15	30	0	30	6.85	0	6.85	3.3333	8.22008
		1	0	82.51	30	0	30	17.49	0	17.49	3.3333	20.9882
		0.1	-1	58.65	30	0	30	41.35	0	41.35	3.3333	49.6205
		0.01	-2	27.49	30	0	30	72.51	0	72.51	3.3333	87.0129
EC				0	30	0	30	100	0	100	3.3333	120

**4e**	IC_{50}	conc	log	%inh	T2	T1	ΔT	RFU2	RFU1	ΔRFU	slope	K.Activity
		10	1	92.94	30	0	30	7.06	0	7.06	3.3333	8.47208
		1	0	80.41	30	0	30	19.59	0	19.59	3.3333	23.5082
		0.1	-1	55.05	30	0	30	44.95	0	44.95	3.3333	53.9405
		0.01	-2	28.94	30	0	30	71.06	0	71.06	3.3333	85.2729
EC				0	30	0	30	100	0	100	3.3333	120

**4g**	IC_{50}	conc	log	%inh	T2	T1	ΔT	RFU2	RFU1	ΔRFU	slope	K.Activity
		10	1	85.71	30	0	30	14.29	0	14.29	3.3333	17.1482
		1	0	65.69	30	0	30	34.31	0	34.31	3.3333	41.1724
		0.1	-1	34.11	30	0	30	65.89	0	65.89	3.3333	79.0688
		0.01	-2	20.56	30	0	30	79.44	0	79.44	3.3333	95.329
EC				0	30	0	30	100	0	100	3.3333	120

**4h**	IC_{50}	conc	log	%inh	T2	T1	ΔT	RFU2	RFU1	ΔRFU	slope	K.Activity
		10	1	86.83	30	0	30	13.17	0	13.17	3.3333	15.8042
		1	0	70.09	30	0	30	29.91	0	29.91	3.3333	35.8924

		0.1	-1	35.84	30	0	30	64.16	0	64.16	3.3333	76.9928
		0.01	-2	17.87	30	0	30	82.13	0	82.13	3.3333	98.557
EC				0	30	0	30	100	0	100	3.3333	120
**Cabozantinib**	IC_{50}	conc	log	%inh	T2	T1	ΔT	RFU2	RFU1	ΔRFU	slope	K.Activity
		10	1	93.09	30	0	30	6.91	0	6.91	3.3333	8.29208
		1	0	84.36	30	0	30	15.64	0	15.64	3.3333	18.7682
		0.1	-1	62.41	30	0	30	37.59	0	37.59	3.3333	45.1085
		0.01	-2	37.51	30	0	30	62.49	0	62.49	3.3333	74.9887
EC				0	30	0	30	100	0	100	3.3333	120

Figure 60: c-Met IC_{50} calculations of compounds **2c, 2f, 4b, 4e, 4g, 4h** and cabozantinib.

2c

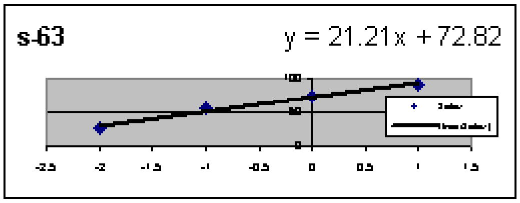

2f

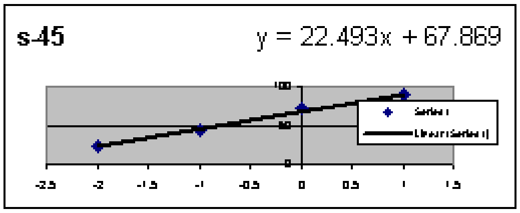

4b

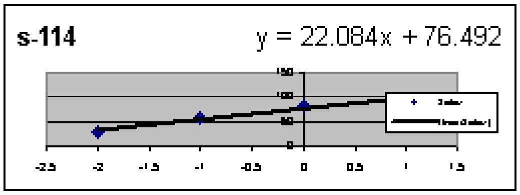

4e

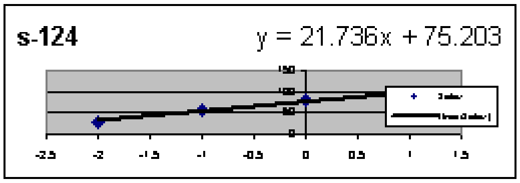

<table>
<tr>
<td>4g
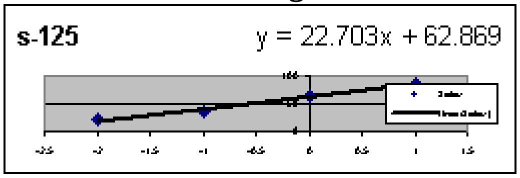
</td>
<td>4h
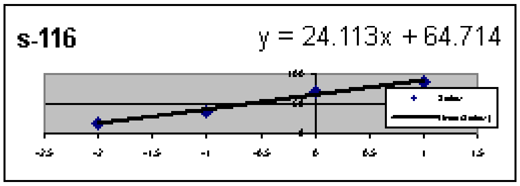
</td>
</tr>
<tr>
<td>Cabozantinib
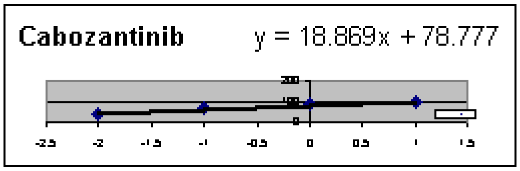
</td>
<td></td>
</tr>
</table>

Table 3: VEGFR-2 IC_{50} calculations of compounds **2c, 2f, 4b, 4e, 4g, 4h** and cabozantinib.

**2c**	IC_{50}	conc	log	%inh	T2	T1	ΔT	RFU2	RFU1	ΔRFU	slope	K.Activity
		10	1	88.96	30	0	30	11.04	0	11.04	3.3333	13.2481
		1	0	76.46	30	0	30	23.54	0	23.54	3.3333	28.2483
		0.1	-1	56.18	30	0	30	43.82	0	43.82	3.3333	52.5845
		0.01	-2	35.32	30	0	30	64.68	0	64.68	3.3333	77.6168
EC				0	30	0	30	100	0	100	3.3333	120

**2f**	IC_{50}	conc	log	%inh	T2	T1	ΔT	RFU2	RFU1	ΔRFU	slope	K.Activity
		10	1	85.96	30	0	30	14.04	0	14.04	3.3333	16.8482
		1	0	68.26	30	0	30	31.74	0	31.74	3.3333	38.0884
		0.1	-1	43.39	30	0	30	56.61	0	56.61	3.3333	67.9327
		0.01	-2	18.54	30	0	30	81.46	0	81.46	3.3333	97.753
EC				0	30	0	30	100	0	100	3.3333	120

**4b**	IC_{50}	conc	log	%inh	T2	T1	ΔT	RFU2	RFU1	ΔRFU	slope	K.Activity
		10	1	90.09	30	0	30	9.91	0	9.91	3.3333	11.8921
		1	0	78.48	30	0	30	21.52	0	21.52	3.3333	25.8243
		0.1	-1	60.12	30	0	30	39.88	0	39.88	3.3333	47.8565
		0.01	-2	38.24	30	0	30	61.76	0	61.76	3.3333	74.1127
EC				0	30	0	30	100	0	100	3.3333	120

**4e**	IC_{50}	conc	log	%inh	T2	T1	ΔT	RFU2	RFU1	ΔRFU	slope	K.Activity
		10	1	86.83	30	0	30	13.17	0	13.17	3.3333	15.8042
		1	0	73.09	30	0	30	26.91	0	26.91	3.3333	32.2923
		0.1	-1	50.29	30	0	30	49.71	0	49.71	3.3333	59.6526
		0.01	-2	33.05	30	0	30	66.95	0	66.95	3.3333	80.3408

EC				0	30	0	30	100	0	100	3.3333	120
**4g**	IC_{50}	conc	log	%inh	T2	T1	ΔT	RFU2	RFU1	ΔRFU	slope	K.Activity
		10	1	88.15	30	0	30	11.85	0	11.85	3.3333	14.2201
		1	0	70.58	30	0	30	29.42	0	29.42	3.3333	35.3044
		0.1	-1	52.07	30	0	30	47.93	0	47.93	3.3333	57.5166
		0.01	-2	27.96	30	0	30	72.04	0	72.04	3.3333	86.4489
EC				0	30	0	30	100	0	100	3.3333	120
**4h**	IC_{50}	conc	log	%inh	T2	T1	ΔT	RFU2	RFU1	ΔRFU	slope	K.Activity
		10	1	82.49	30	0	30	17.51	0	17.51	3.3333	21.0122
		1	0	60.74	30	0	30	39.26	0	39.26	3.3333	47.1125
		0.1	-1	40.45	30	0	30	59.55	0	59.55	3.3333	71.4607
		0.01	-2	18.58	30	0	30	81.42	0	81.42	3.3333	97.705
EC				0	30	0	30	100	0	100	3.3333	120
**Cabozantinib**	IC_{50}	conc	log	%inh	T2	T1	ΔT	RFU2	RFU1	ΔRFU	slope	K.Activity
		10	1	91.78	30	0	30	8.22	0	8.22	3.3333	9.8641
		1	0	78.54	30	0	30	21.46	0	21.46	3.3333	25.7523
		0.1	-1	58.06	30	0	30	41.94	0	41.94	3.3333	50.3285
		0.01	-2	30.75	30	0	30	69.25	0	69.25	3.3333	83.1008
EC				0	30	0	30	100	0	100	3.3333	120

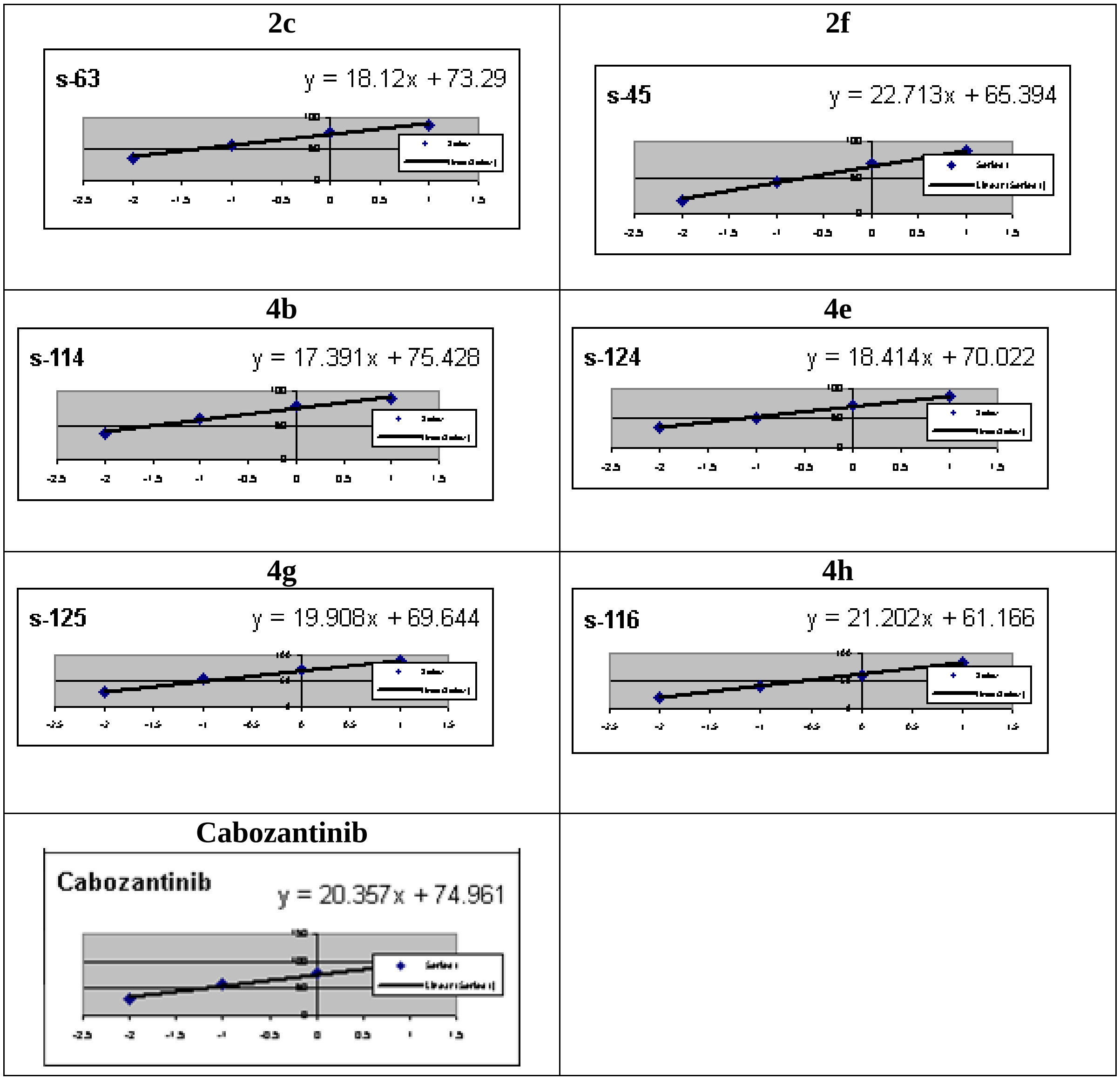

Figure 61: VEGFR-2 IC_{50} calculations of compounds **2c, 2f, 4b, 4e, 4g, 4h** and cabozantinib.

Table 4: Physicochemical parameters and druglikeness of target compounds and cabozantinib.

#	MW	RB	HBA	HBD	MR	TPSA	iLOGP	Number of violations					Bioavailability
								Lipinski	Ghose	Veber	Egan	Muegge	Score
**2a**	295.29	4	3	2	83.92	83.96	1.97	0	0	0	0	0	0.55
**2b**	309.32	5	3	2	87.19	83.96	2.02	0	0	0	0	0	0.55

**2c**	301.34	4	3	2	84.63	83.96	2.26	0	0	0	0	0	0.55
**2d**	247.25	3	3	1	67.61	75.17	1.85	0	0	0	0	0	0.55
**2e**	287.31	3	3	1	83.83	75.17	2.27	0	0	0	0	0	0.55
**2f**	289.29	3	4	1	80.11	84.4	2.03	0	1	0	0	0	0.55
**2g**	305.29	7	5	2	78.41	110.26	1.85	0	1	0	0	0	0.55
**3**	291.26	6	5	4	73.22	139.08	0.33	0	1	0	1	0	0.55
**4a**	379.37	8	5	3	103.49	125.42	2.01	0	0	0	0	0	0.55
**4b**	424.37	9	7	3	112.32	171.24	1.85	1	0	1	1	1	0.55
**4c**	409.4	9	6	3	109.99	134.65	1.95	0	0	0	1	0	0.55
**4d**	369.33	8	6	3	95.76	138.56	1.65	0	1	0	1	0	0.55
**4e**	371.39	7	5	3	100.93	125.42	2.47	0	0	0	0	0	0.55
**4f**	420.38	7	6	4	113.82	154.52	2.25	1	1	1	1	1	0.55
**4g**	355.35	6	5	2	94.43	118.85	1.51	0	0	0	0	0	0.55
**4h**	333.32	5	5	3	82.44	157.87	1.47	0	0	1	1	1	0.55
**4i**	426.45	10	4	5	114.74	169.21	2.49	0	0	1	1	1	0.55
**4j**	408.43	6	4	3	109.50	149.66	2.06	0	0	1	1	0	0.55
**CBZ**	501.51	10	7	2	136.59	98.78	3.6	1	2	0	0	1	0.55

CBZ: cabozantinib; RB: rotatable bonds; MR: Molar Refractivity; TPSA: Topological polar surface area

Table 5: Solubility and pharmacokinetics of target compounds and cabozantinib.

#	ESOL Log S	ESOL Class	GI absorption	BBB permeant	Pgp substrate	CYP1A2 inhibitor	CYP2C19 inhibitor	CYP2C9 inhibitor	CYP2D6 inhibitor	CYP3A4 inhibitor
**2a**	-2.91	Soluble	High	No	No	No	No	No	No	No
**2b**	-2.87	Soluble	High	No	No	No	No	No	No	No
**2c**	-2.9	Soluble	High	No	No	No	No	No	No	No
**2d**	-1.69	Very soluble	High	No	No	No	No	No	No	No
**2e**	-2.41	Soluble	High	No	No	No	No	No	No	No
**2f**	-1.65	Very soluble	High	No	No	No	No	No	No	No
**2g**	-1.77	Very soluble	High	No	No	No	No	No	No	No
**3**	-0.7	Very soluble	Low	No	No	No	No	No	No	No
**4a**	-2.69	Soluble	High	No	No	No	No	No	No	No
**4b**	-2.75	Soluble	Low	No	No	No	No	No	No	No
**4c**	-2.76	Soluble	High	No	Yes	No	No	No	No	No
**4d**	-2.23	Soluble	Low	No	No	No	No	No	No	No
**4e**	-2.27	Soluble	High	No	Yes	No	No	No	No	No
**4f**	-2.89	Soluble	Low	No	Yes	No	No	No	No	No
**4g**	-2.51	Soluble	High	No	No	No	No	No	No	No
**4h**	-2.15	Soluble	Low	No	No	No	No	No	No	No

**4i**	-2.73	Soluble	Low	No	Yes	No	No	No	No	No
**4j**	-3.34	Soluble	Low	No	No	No	No	No	No	No
**CBZ**	-6.13	Poorly soluble	High	No	Yes	No	Yes	Yes	Yes	Yes
